# Supplementary material for: The Genetic Legacy of Zoroastrianism in Iran and India: Insights into Population Structure, Gene Flow, and Selection
Source: Am J Hum Genet. 2017 Aug 24;101(3):353–68. doi: 10.1016/j.ajhg.2017.07.013 (PMC5590844; doi:10.1016/j.ajhg.2017.07.013)
Supplement: Document S1. Figures S1–S13 and Tables S1 and S4–S12 [file mmc1.pdf]

The American Journal of Human Genetics, Volume *101*

Supplemental Data

The Genetic Legacy of Zoroastrianism in Iran and India: Insights into Population Structure, Gene Flow, and Selection

Saioa López, Mark G. Thomas, Lucy van Dorp, Naser Ansari-Pour, Sarah Stewart, Abigail L. Jones, Erik Jelinek, Lounès Chikhi, Tudor Parfitt, Neil Bradman, Michael E. Weale, and Garrett Hellenthal

Supplemental Figures

Figure S1. FineSTRUCTURE heatmap and tree. Inferred proportion of genome-wide DNA that each of the clusters inferred by fineSTRUCTURE (columns) copy from each of these clusters (rows), displayed as a heatmap. The tree at top shows hierarchical merging of the clusters inferred by fineSTRUCTURE. The green lines show how the 207 clusters at the finest level of the tree were classified into 50 groups (a separate cluster we generated that contains only the Neolithic Iranian farmer sample "WC1" is not shown in this heatmap). The populations analysed here include the novel samples generated in this work (Indian_Zoroastrian, Indian Hindu, Iranian_Zoroastrian, Iranian_Fars) plus the populations originally labelled in the Lazaridis dataset as: Onge, Mala, Tiwari, Kharia, Lodhi, Vishwabrahmin, GujaratiD_GIH, GujaratiB_GIH, GujaratiA_GIH, GujaratiC_GIH, Cochin_Jew, Iranian, Iranian_Bandari, Iranian_GM, Iranian_Shi, Iranian_Lor, Iranian_Jew, Brahui, Balochi, Hazara, Makrani, Sindhi, Pathan, Kalash, Burusho, Punjabi_Lahore_PJL, Druze, BedouinB, BedouinA, Palestinian, Syrian, Lebanese, Jordanian, Yemen, Georgian_Megrels, Abkhasian, Armenian, Lebanese_Christian, Lebanese_Muslim, Assyrian, Yemenite_Jew, Turkish_Jew, Turkish_Kayseri, Turkish_Balikesir, Turkish, Turkish_Istanbul, Turkish_Adana, Turkish_Trabzon, Turkish_Aydin, Iraqi_Jew, Georgian_Jew, AltaiNea, DenisovaPinky, and additional ancient DNA (aDNA) samples we label here as UstIshim, Mota, KK1, LBK, Loschbour, NE1, Bar8, WC1).

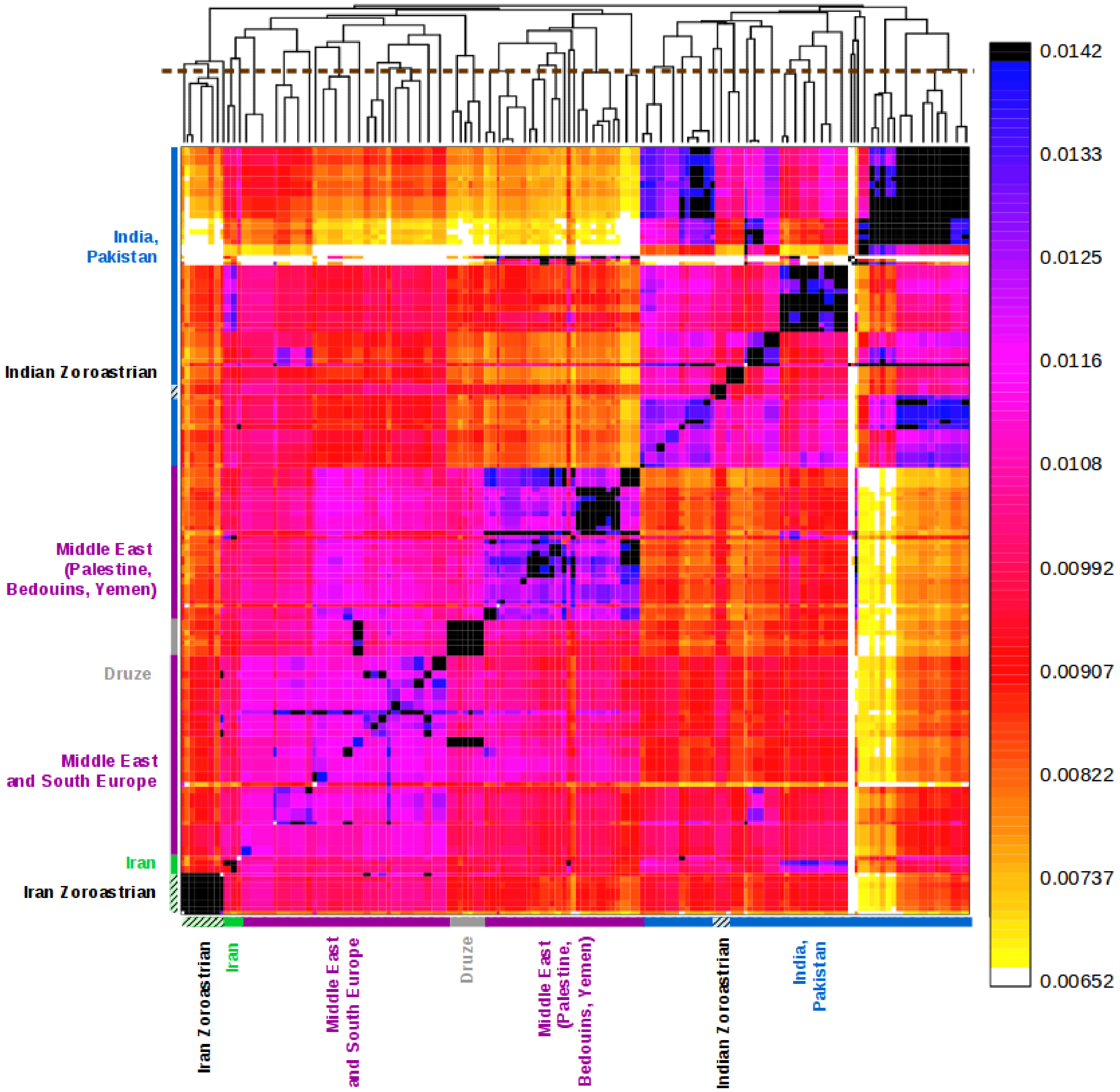
India, Pakistan
Indian Zoroastrian
Middle East (Palestine, Bedouins, Yemen)
Druze
Middle East and South Europe
Iran
Iran Zoroastrian
Iran Zoroastrian
Iran
Middle East and South Europe
Druze
Middle East (Palestine, Bedouins, Yemen)
Indian Zoroastrian
India, Pakistan
0.0142
0.0133
0.0125
0.0116
0.0108
0.00992
0.00907
0.00822
0.00737
0.00652

Figure S2: Comparison of the pairwise F_{XY} based on the "all donors painting" (upper left triangle) and F_{XY} based on the "non Iranian/Parsi donors painting" (lower right triangle) for the Iranian groups. Note that Iranian Zoroastrians are not very strongly differentiated from other Iranian groups in the bottom triangle, indicating isolation effects rather than admixture from outside groups are likely driving differences in the top left triangle.

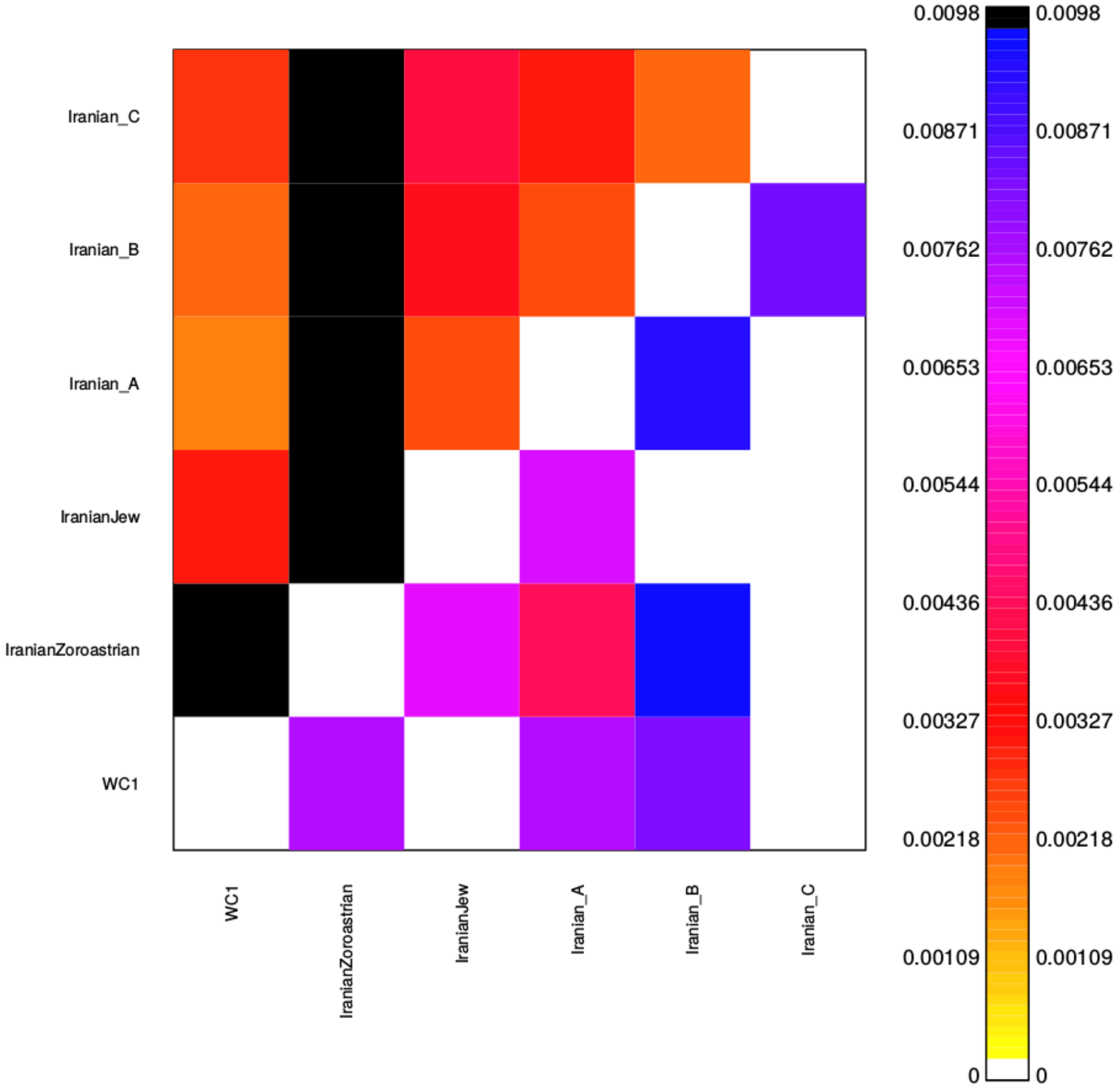

Figure S3: Comparison of the pairwise F_{XY} based on the "all donors painting" (upper left triangle) and F_{XY} based on the "non Indian donors painting" (lower right triangle) for the Indian groups.

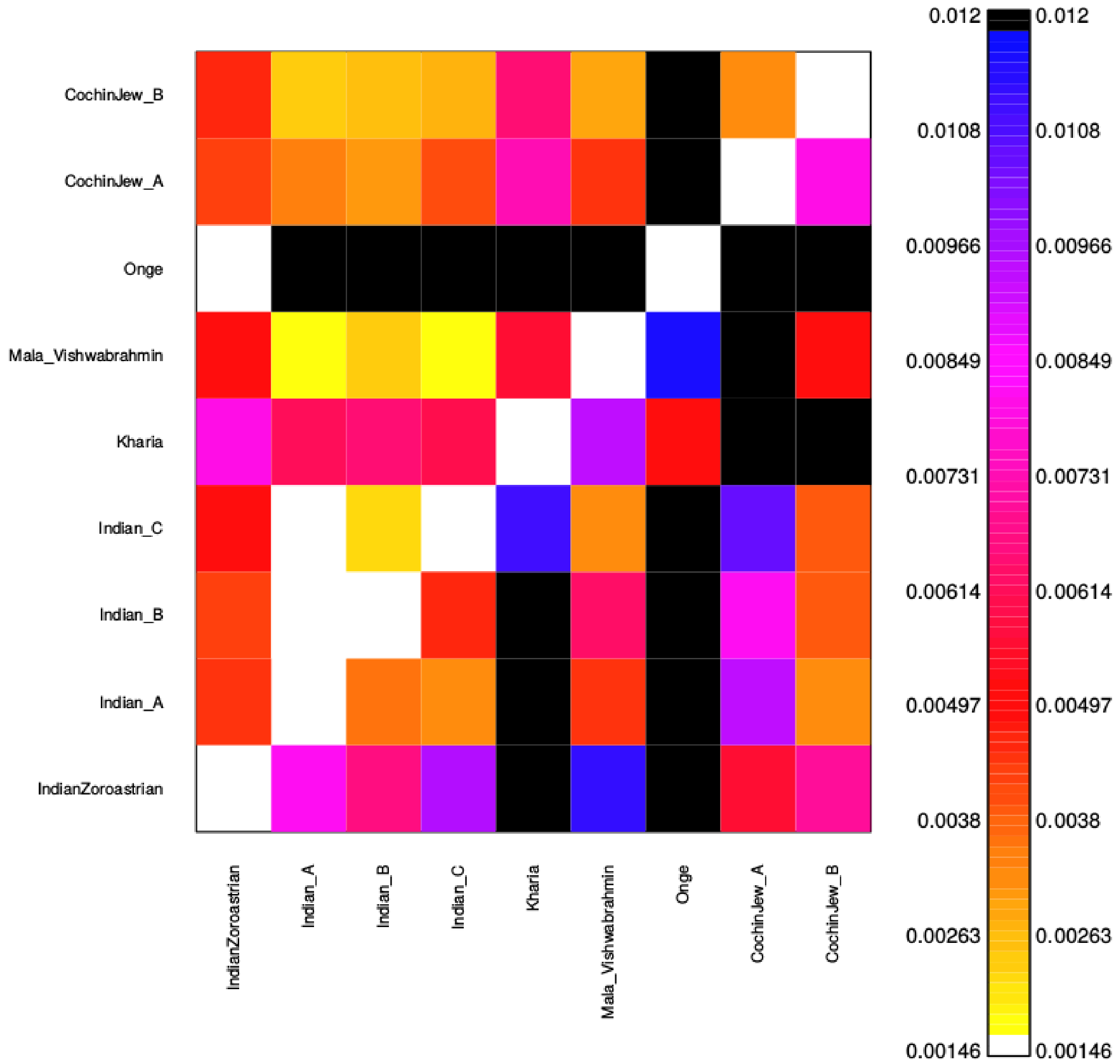

Figure S4. Principal Component Analysis (PCA) of the South-West Eurasian populations included in the merge. Iranian and Indian group labels are highlighted in green and blue, respectively.

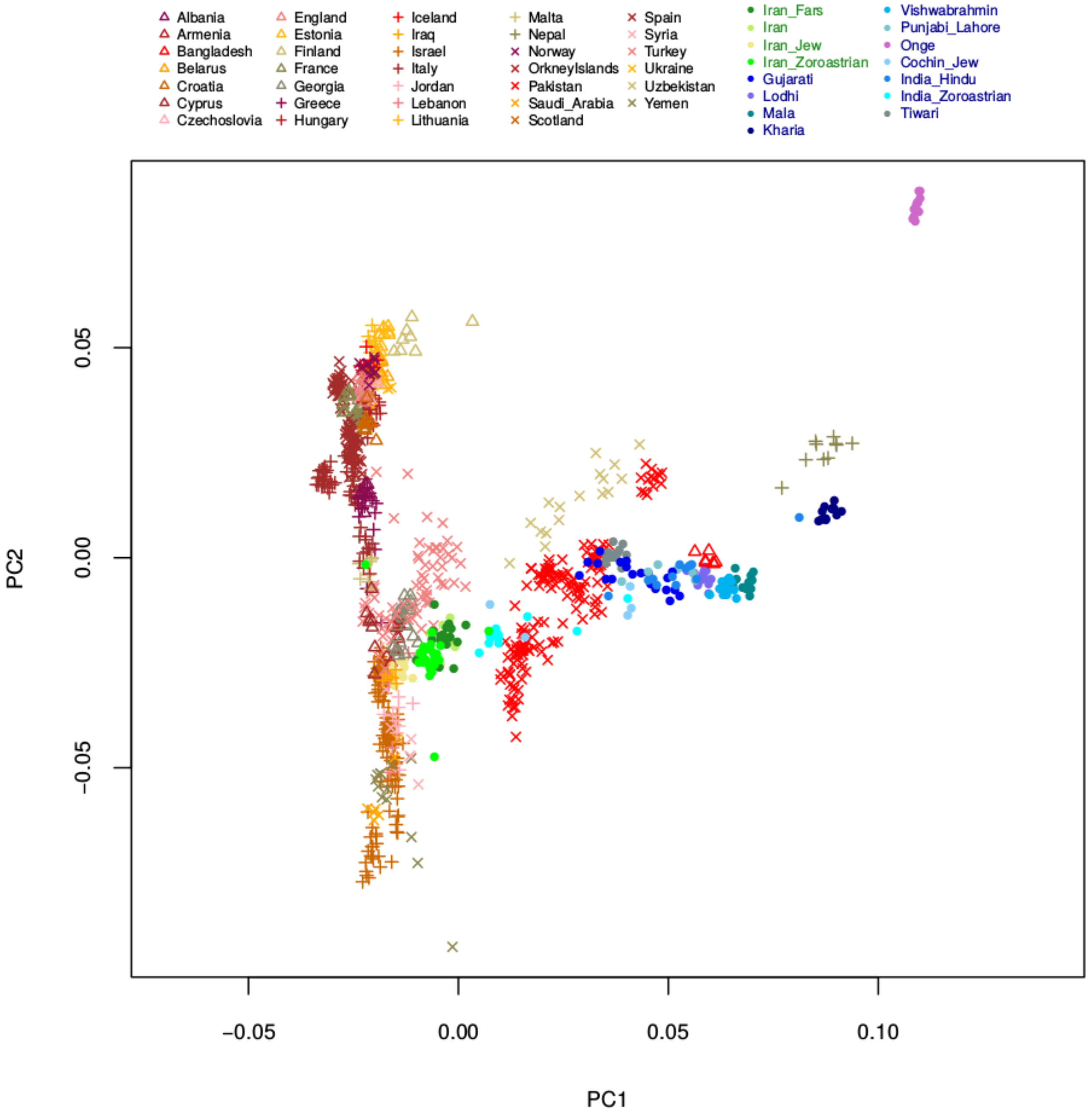

Figure S5. Proportion of haplotypes each Iranian, Indian, Pakistani and Armenian individual (columns) shares with modern groups (rows) from different geographic regions (y-axis labels). The right heatmap shows these proportions for (left to right): the ancient Iranian farmer WC1, a labeled Iranian Zoroastrian (YZ020) that clusters with non-Iranian Zoroastrians, averaged across all sampled Bandari, averaged across all sampled Iranian Fars excluding the outliers, averaged across all sampled Iranian Zoroastrians excluding the outliers, a labeled Iranian Fars individual (IREJ-T053) that clusters with the Iranian Zoroastrians, averaged across all sampled Iranian Jews and a labeled Iranian Zoroastrian (YZ024) that clusters with other non-Iranian individuals (most strongly with Sephardi Jews sampled from Turkey). Green rectangles enclose samples that cluster together using fineSTRUCTURE.

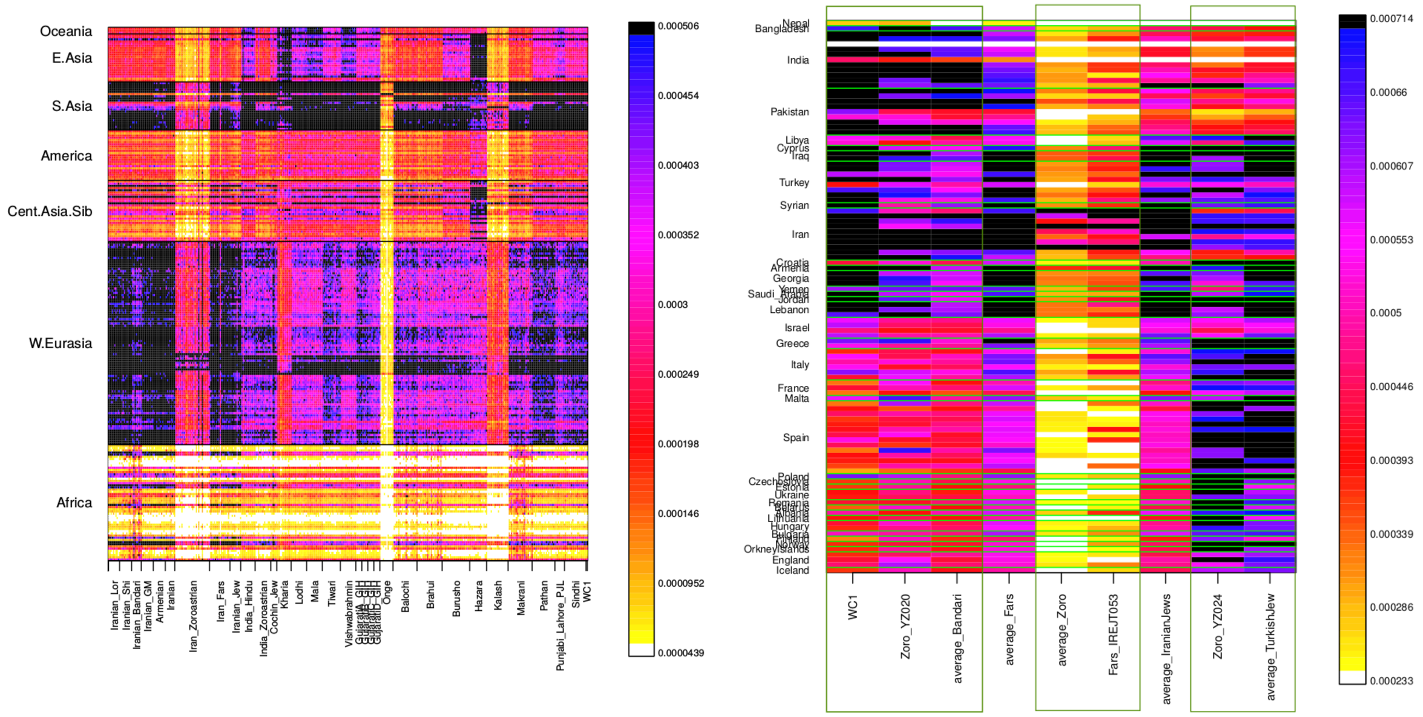

Figure S6: Pairwise comparison of the three methods used to assess genetic homogeneity: CHROMOPAINTER's inferred average haplotype segment sizes (in cM) versus PI_HAT values inferred by PLINK v1.9 and fastIBD inferred IBD coefficient (FIBD). Median and 95% empirical quantile values across all individuals (segment size) or across all pairwise comparisons of individuals (PI_HAT and FIBD estimates) are shown.

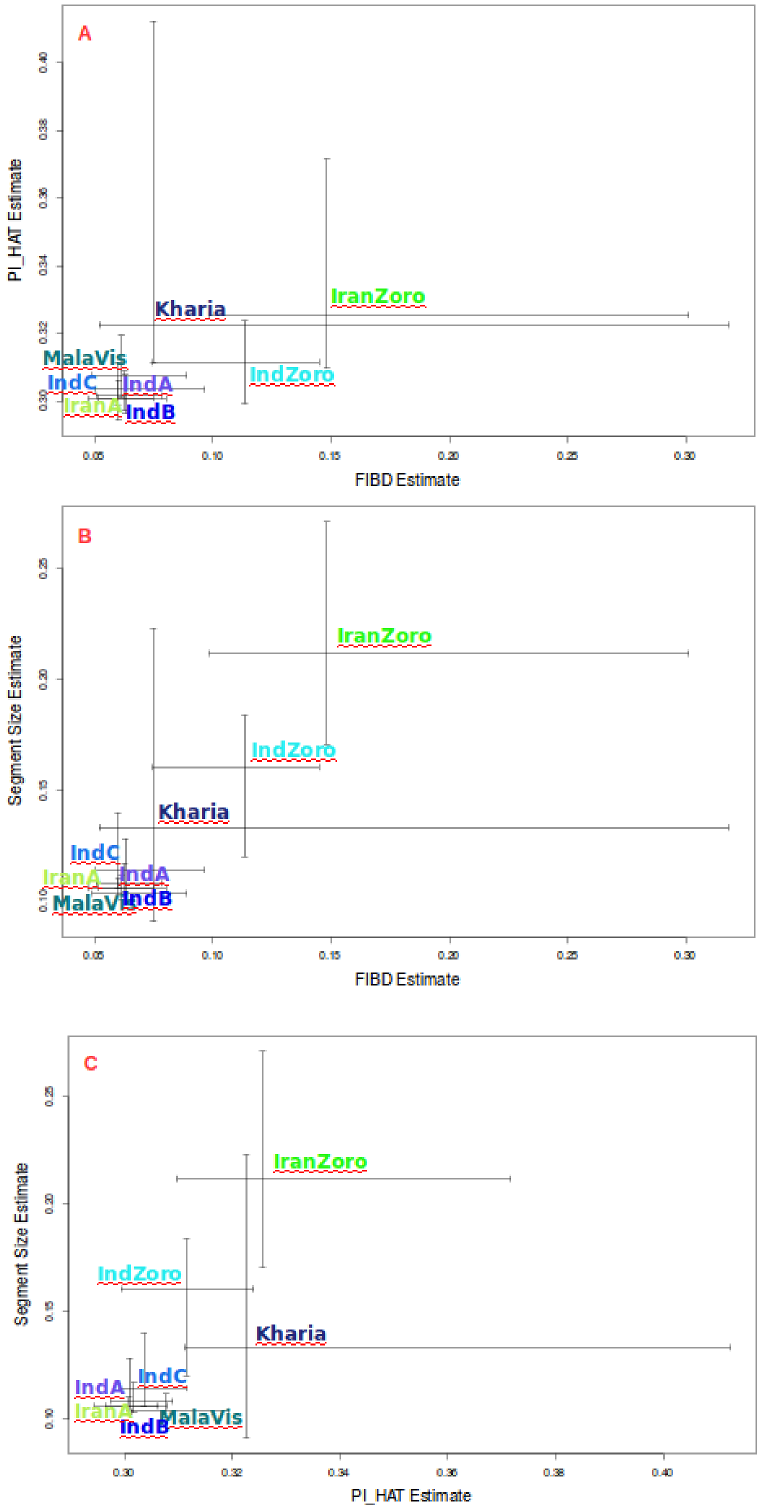

Figure S7: Inferred r**ecent admixture in India and Iran when excluding Iranian and Indian populations as donors in the painting profiles (i.e. "non-Indian/Iranian donors painting"),** using surrogates from Europe (brown), Middle East (orange; Yemen in dark orange), Africa (light green), Pakistan (red), Bangladesh (pink), Cambodia (cyan), Iran (dark green) and India (blue) and of Jewish heritage (purple), plus the ancient samples WC1 (yellow), Ust'Ishim (dark grey) and Bar8 (grey). Proportions of ancestry inferred from each surrogate group are represented in the pie graphs, with all contributing groups highlighted in non-grey in the map in the left bottom corner. Dates of admixture and 95% confidence intervals inferred by GLOBETROTTER are shown on the top right, colored according to the surrogate that best reflects the minor contributing admixture source. GLOBETROTTER coancestry curves, illustrating the weighted probability that DNA segments separated by distance x (in cM) match to the two admixture surrogates given in the title, for Parsis (Iranian Zoroastrians vs Indian_C) and Iranian Zoroastrians (Indian Zoroastrians vs Greek) in the bottom right.

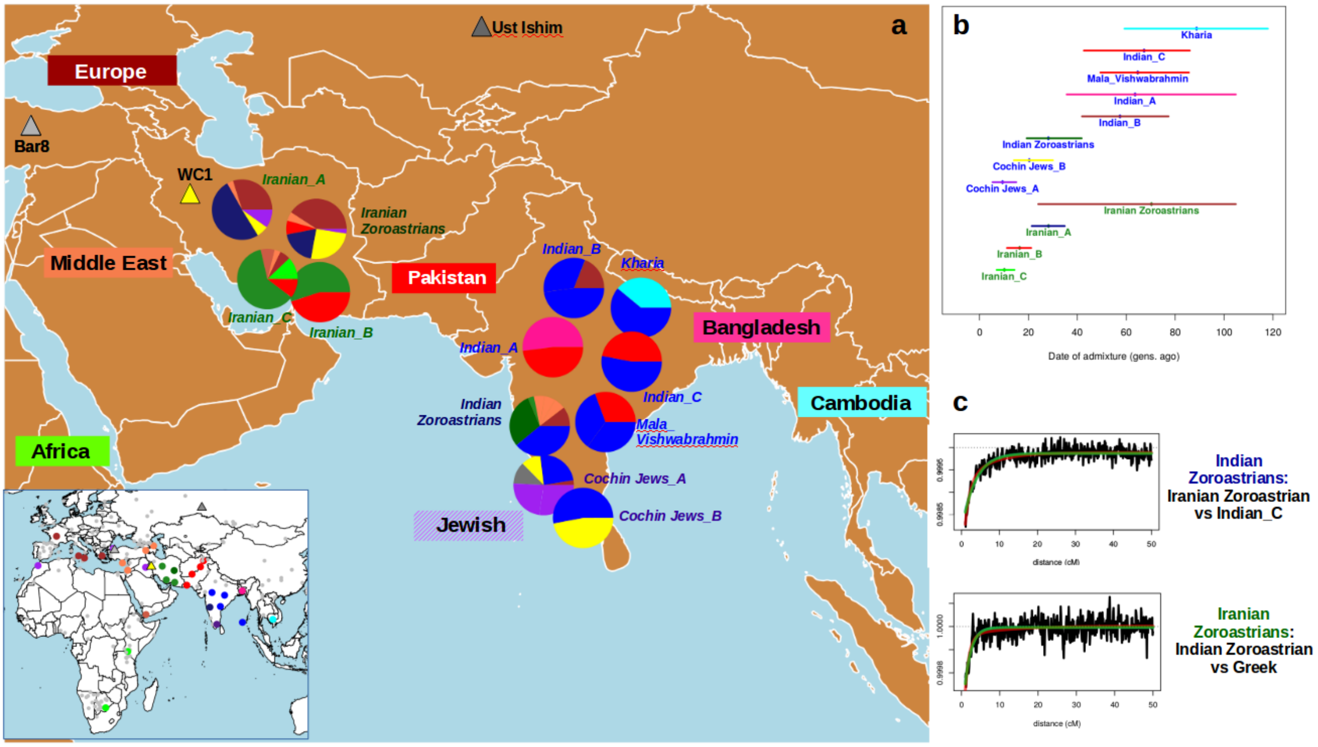

Figure S8: Maximum likelihood trees constructed with TreeMix between Iranian and Indian clusters (Indian_C, Indian Zoroastrians, Iranian_A and Iranian Zoroastrians) using Yoruba as an outgroup, for 0-1 (a-b) migration events. Edges show the direction of gene flow between populations.

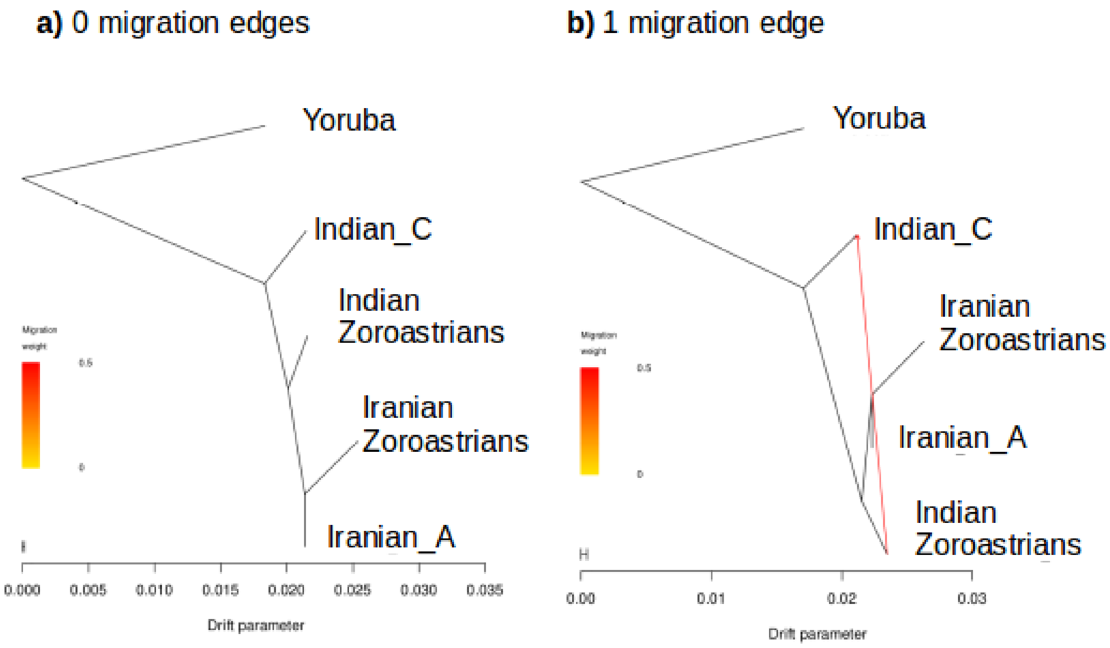

Figure S9: Residuals for the maximum likelihood trees constructed with TreeMix between Iranian and Indian clusters (Indian_C, Indian Zoroastrians, Iranian_A and Iranian Zoroastrians) using Yoruba as an outgroup, for 0-1 (a-b) migration events. Positive residuals indicate candidate populations for admixture events, as they are more closely related to each other in the data than predicted by the best-fitting tree.

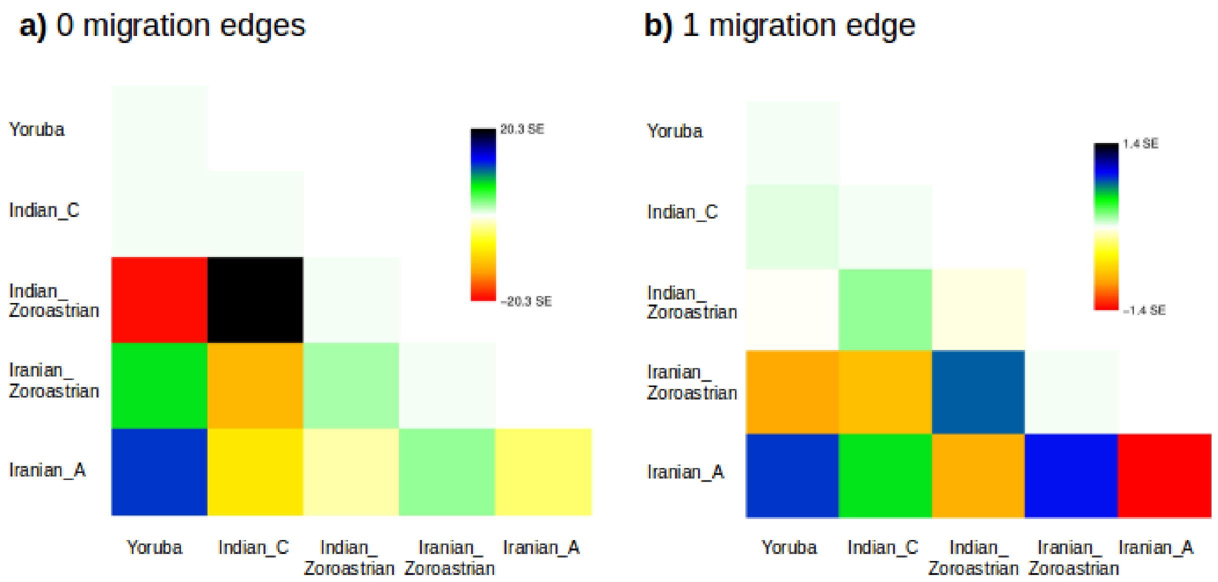

Figure S10: PCO plots of the 6 populations based on pairwise F_{ST} values of Yhg (above) and iMhg (below) frequencies, which summarise 88.5% and 93.2% of the variation, respectively.

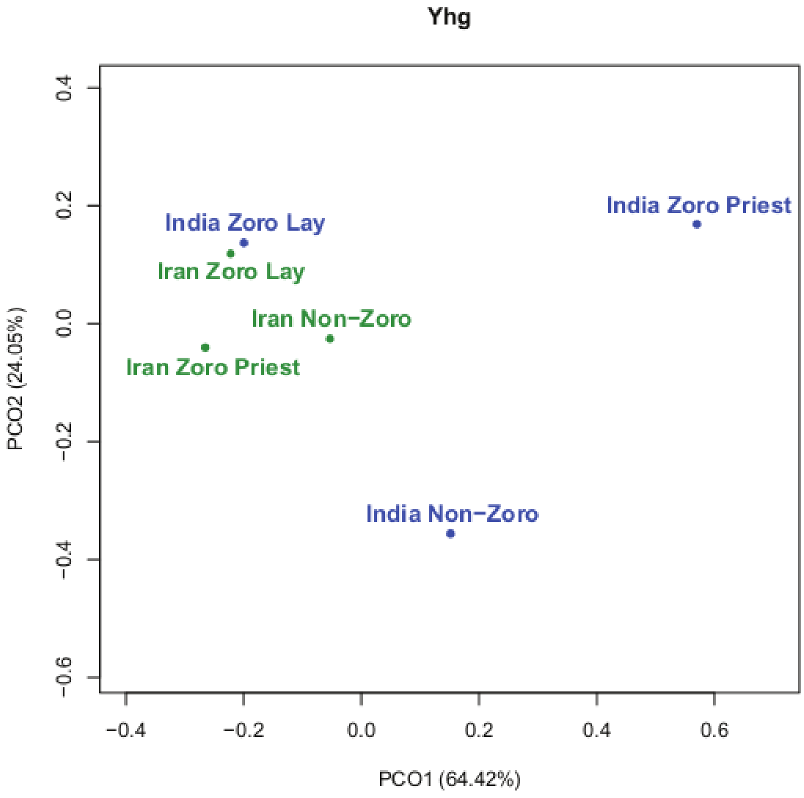

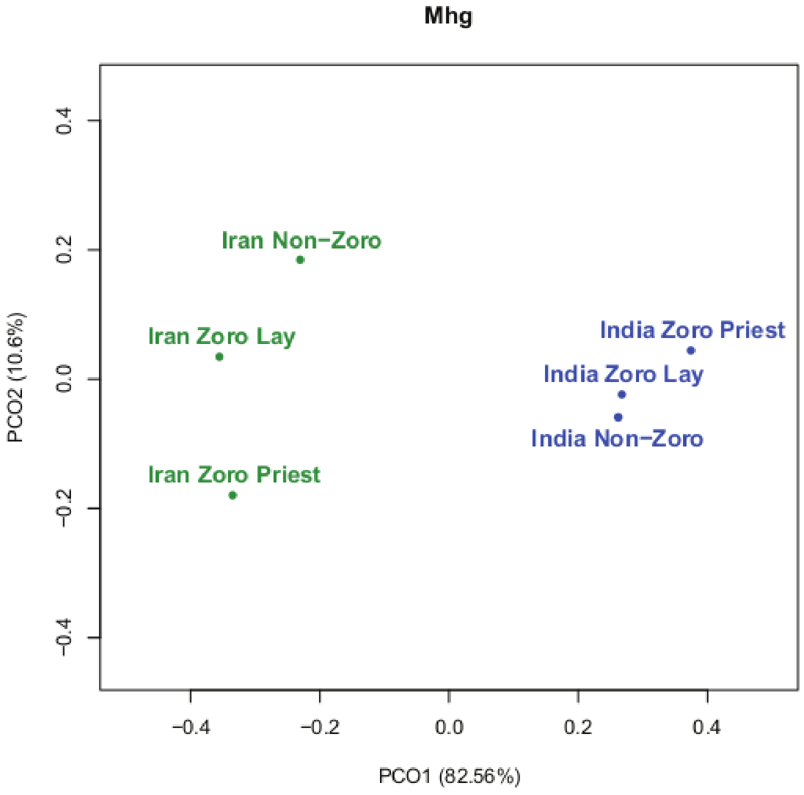

Figure S11: Y-chromosome haplogroups (Yhg), denoted according to the Y-chromosome consortium and the most recent nomenclature of ISOGG defined by the 12 UEP biallelic loci. Each node represents a Yhg; text on each branch indicates the mutation that defines the given nodes.

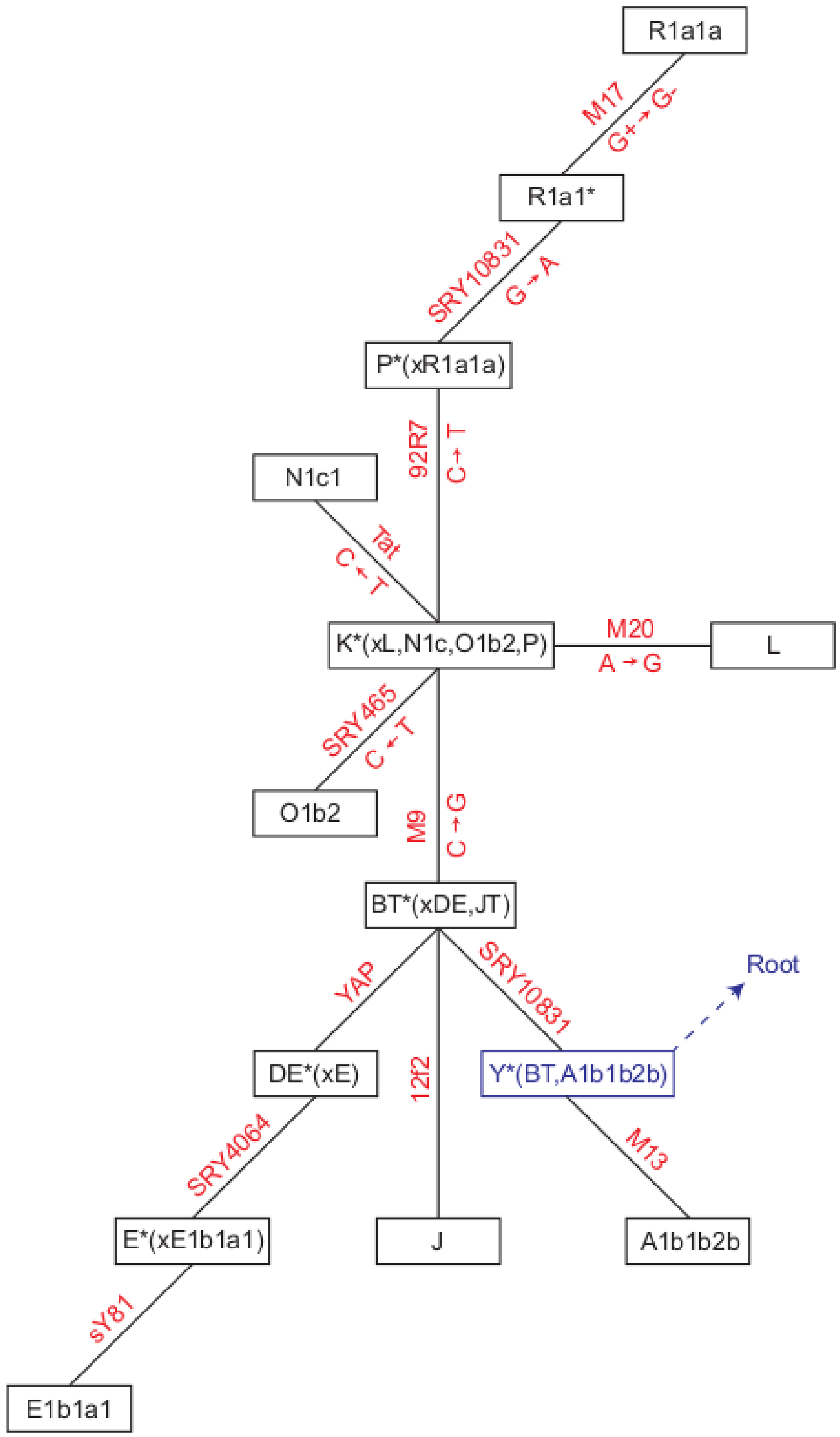

Figure S12. XP-EHH scores in Parsis (top) and Iranian Zoroastrians (bottom). Non-Zoroastrian Indian and Iranian populations were used as reference populations, respectively, for the XP-EHH test. The blue lines show the significance threshold values of 0.01% estimated from an empirical distribution of XP-EHH values (see Methods).

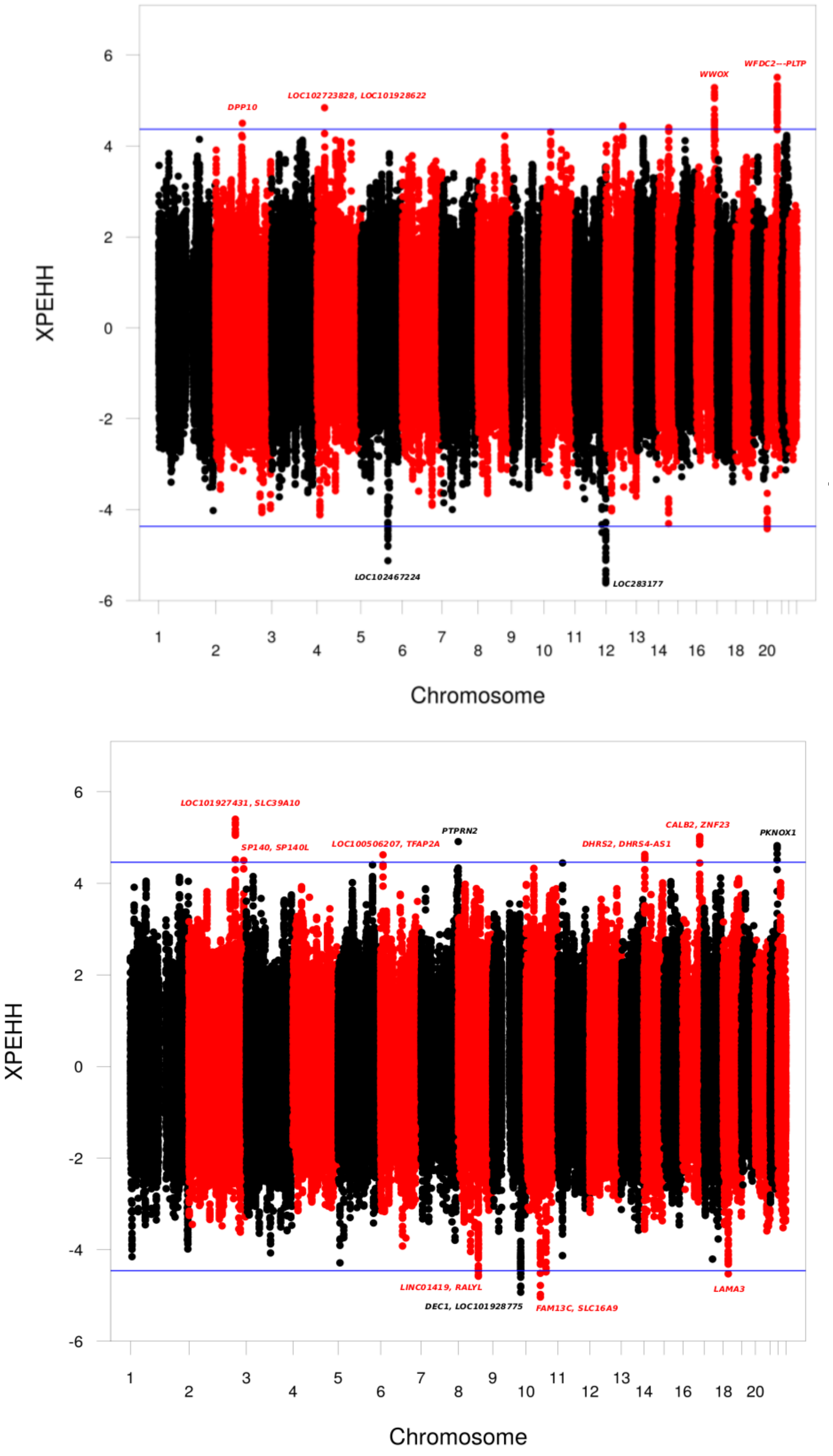

Figure S13: Empirical qqplots based on 100 permutations (x-axis), showing the mean values across permutations (x-axis) versus the real values (y-axis) observed for the Indian and Iranian populations.

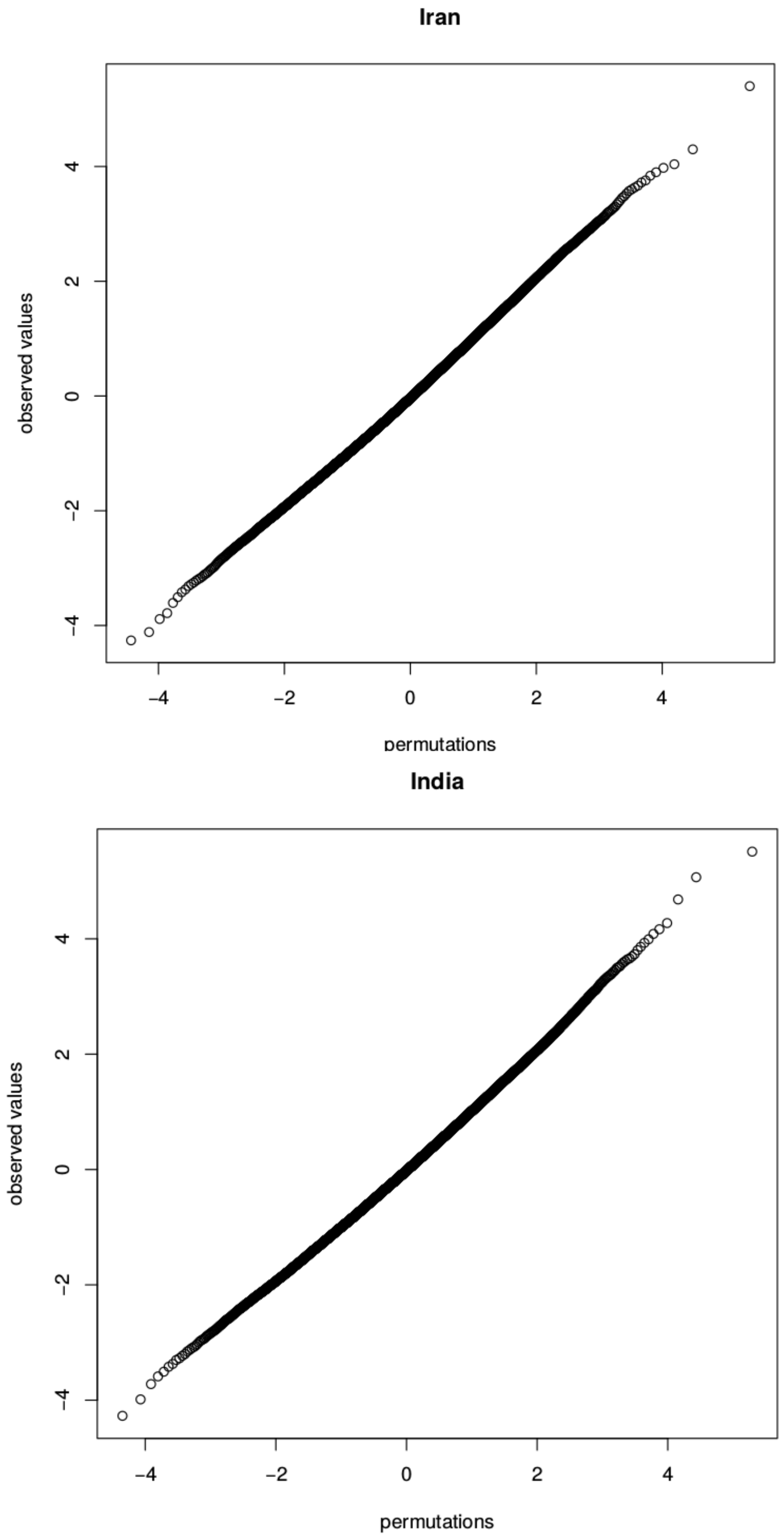

Supplemental Tables

Table S1. Description of the samples collected for this work and number of individuals included in each analysis.

Population	Collection place	Total samples	ChrY	mtDNA	Human Origins autosomal data
**Non-Zoroastrian from India**	-	49	41	46	12
**Non-Zoroastrian from Iran**	-	193	172	193	17
**Lay-Zoroastrian from India**	Bombay	56	54	53	7
	Dubai	8	8	8	0
	Navsari	37	37	37	4
	Surat	11	11	11	2
	UK	8	8	8	0
	Udwada	4	4	4	0
	Total	124	122	121	13
**Zoroastrian priests from India**	Bombay	13	12	12	0
	Dubai	10	10	10	0
	Navsari	20	20	20	0
	Surat	9	9	9	0
	UK	4	4	4	0
	Udwada	16	16	16	0
	Total	72	71	71	0
**Lay-Zoroastrian from Iran**	Isfahan	2	2	2	2
	Shiraz	8	7	8	6
	Tehran	19	18	18	9
	UK	1	0	1	0
	Yazd	50	49	50	12
	Total	80	76	79	29
**Zoroastrian priests from Iran**	Shiraz	1	1	1	0
	Tehran	3	3	3	0
	Yazd	4	4	4	0
	Total	8	8	8	0
**TOTAL**		526	490	518	71

Table S2. Number of individuals from each population label included in all the clusters inferred by fineSTRUCTURE.

Separate file: Table_S2.xls

Table S3. F_{ST}, TVD and F_{XY} between the Iranian, Indian, Pakistani and Armenian clusters

Separate file: Table_S3.xls

Table S4. f3 statistics estimated with ADMIXTOOLS, when using Parsis as a target group. Only |Z| scores >2 are shown.

**Source1**	**Source2**	**Target**	**F_3**	**std. Err**	**Z**
LBK	Vishwabrahmin	India_Zoroastrian	-0.002923	0.000733	-3.987
LBK	Mala	India_Zoroastrian	-0.002549	0.000761	-3.35
Lebanese_Christian	Kharia	India_Zoroastrian	-0.001434	0.000454	-3.161
Armenian	Kharia	India_Zoroastrian	-0.001397	0.000455	-3.073
NE1	Mala	India_Zoroastrian	-0.002263	0.00074	-3.06
Georgian_Megrels	Kharia	India_Zoroastrian	-0.00131	0.000455	-2.877
Bar8	Mala	India_Zoroastrian	-0.001909	0.000705	-2.708
NE1	Vishwabrahmin	India_Zoroastrian	-0.002017	0.000754	-2.675
LBK	Kharia	India_Zoroastrian	-0.002253	0.000914	-2.466
Turkish_Trabzon	Kharia	India_Zoroastrian	-0.001073	0.00046	-2.331
Assyrian	Kharia	India_Zoroastrian	-0.001037	0.000445	-2.33
Kharia	Druze	India_Zoroastrian	-0.00093	0.000403	-2.306
Iraqi_Jew	Kharia	India_Zoroastrian	-0.001088	0.000511	-2.131

Table S5. Admixture events inferred by GLOBETROTTER using modern populations (fineSTRUCTURE clusters) as surrogates under the "all donors painting". AdmDate=number of generations (date in years in brackets, using the formula 1950-28*(g+1) that assumes 28 years per generation g of admixture) since the admixture event occurred; S=source number; "prop" the proportion contributed by each source; "Source composition" indicates the single sampled group that best matches the inferred genetic make-up for the given source, plus in curly brackets a more detailed inference of the genetic make-up of the source (excluding groups inferred to contribute <=5% to this make-up).

Target	AdmDate	AdmDate (min-max)	S	prop	Source composition
Iranian_A	25 (1222 CE)	20-32 (1026 CE-1362 CE)	S1	0.49	Indian_Pakistani *{Turkish:0.1201 Kumyk:0.1226 Indian_Pakistani:0.2473}*
			S2	0.51	Lebanese_TurkishJew *{Armenian_Assyrian_Turkish:0.0565 Lebanese_TurkishJew:0.4535}*
Iranian_B	18 (1418 CE)	13-24 (1250 CE-558 CE)	S1	0.38	Pakistani *{Iranian_A:0.0656 Pakistani:0.3144}*
			S2	0.62	Iranian_C *{Iranian_C:0.62}*
Iranian_C	10 (1642 CE)	6-13 (1558 CE-1754 CE)	S1	0.14	Kikuyu *{Kikuyu:0.0908 Pathan:0.0838 Lebanese_TurkishJew:0.0996}*
			S2	0.86	Iranian_B *{Iranian_B:0.6468}*
Indian_Zoroastrian	27 (1166 CE)	17-38 (858 CE-1446 CE)	S1	0.36	Indian_A *{Indian_C:0.134 Indian_A:0.2054}*
			S2	0.64	Iranian_A *{Iranian_A:0.64}*
Indian_A	63 (158 CE)	40-107 (1074 BCE-802 CE)	S1	0.46	Mala_Vishwabrahmin *{Mala_Vishwabrahmin:0.46}*
			S2	0.54	Pathan*{Pathan:0.54}*
Indian_B	56 (354 CE)	40-72 (94 BCE-802 CE)	S1	0.28	Italian_Bergamo *{Tajik_Pomiri:0.0581 Italian_Bergamo:0.2219}*
			S2	0.72	Bengali_Bangladesh *{Indian_Pakistani:0.0898 Mala_Vishwabrahmin:0.1321 Indian_A:0.1566 Bengali_Bangladesh:0.3415}*
Indian_C	52 (466 CE)	30-82 (374 BCE-1082 CE)	S1	0.48	Mala_Vishwabrahmin *{Mala_Vishwabrahmin:0.48}*
			S2	0.52	Indian_Pakistani *{Indian_Pakistani:0.52}*
Kharia	84 (430 BCE)	60-107 (1074 BCE-242 CE)	S1	0.48	Cambodian *{Mala_Vishwabrahmin:0.0681 Cambodian:0.4104}*
			S2	0.52	Mala_Vishwabrahmin *{Indian_C:0.1818 Mala_Vishwabrahmin:0.3382}*
Mala_Vishwabrahmin	57 (326 CE)	42-80 (318 BCE-746 CE)	S1	0.45	Indian_Pakistani *{Indian_Pakistani:0.45}*
			S2	0.55	Bengali_Bangladesh *{Bengali_Bangladesh:0.55}*
CochinJew_A	26 (1194 CE)	10-39 (830 CE-1642 CE)	S1	0.47	Moroccan_Jew *{GeorgianJew_IraqiJew:0.1386 Moroccan_Jew:0.2063}*
			S2	0.53	CochinJew_B *{Moroccan_Jew:0.0532 GeorgianJew_IraqiJew:0.0619 CochinJew_B:0.354}*

CochinJew_B	12 (1586 CE)	6-22 (1306 CE-1754 CE)	S1	0.46	CochinJew_A *{CochinJew_A:0.46}*
			S2	0.54	Mala_Vishwabrahmin *{Bengali_Bangladesh:0.2617 Mala_Vishwabrahmin:0.2783}*
Indian_Pakistani	51 (494 CE)	33-64 (130 CE-998 CE)	S1	0.46	Mala_Vishwabrahmin *{Indian_A:0.0861 Mala_Vishwabrahmin:0.3297}*
			S2	0.54	Iranian_A *{Balochi:0.0818 Iranian_A:0.4582}*
Pathan	37 (886 CE)	24-53 (438 CE-1250 CE)	S1	0.46	Iranian_A *{Hungarian_Coriell:0.1124 Iranian_A:0.3476}*
			S2	0.54	Bengali_Bangladesh *{Indian_Pakistani:0.0859 Bengali_Bangladesh:0.4437}*
Kalash	64 (130 CE)	29-101 (906 BCE-1110 CE)	S1	0.34	Bengali_Bangladesh *{Bengali_Bangladesh:0.34}*
			S2	0.66	Tajik_Pomiri *{Pathan:0.1513 Tajik_Pomiri:0.5045}*
Hazara	22 (1306 CE)	20-25 (1222 CE-1362 CE)	S1	0.46	Pathan *{Pathan:0.46}*
			S2	0.54	Kalmyk *{Kalmyk:0.54}*
Burusho	47 (606 CE)	36-61 (214 CE-914 CE)	S1	0.49	Bengali_Bangladesh *{Bengali_Bangladesh:0.49}*
			S2	0.51	Tajik_Pomiri *{Tajik_Pomiri:0.51}*
Makrani	17 (1446 CE)	14-19 (1390 CE-1530 CE)	S1	0.05	Wambo
			S2	0.95	Pakistani *{Iranian_B:0.2814 Pakistani:0.6686}*
Pakistani	24 (1250 CE)	14-34 (970 CE-1530 CE)	S1	0.47	Makrani *{Makrani:0.47}*
			S2	0.53	Balochi *{Balochi:0.53}*
Balochi	14 (1530 CE)	5-20 (1362 CE-1782 CE)	S1	0.46	Pakistani *{Brahui:0.0818 Pakistani:0.3782}*
			S2	0.54	Indian_Pakistani *{Brahui:0.0574 Pathan:0.1773 Indian_Pakistani:0.2725}*
Brahui	18 (1418 CE)	11-24 (1250 CE-1614 CE)	S1	0.48	Balochi *{Balochi:0.48}*
			S2	0.52	Pakistani *{Pakistani:0.52}*
Armenian_Assyrian_ Turkish	29 (1110 CE)	20-39 (830 CE-1362 CE)	S1	0.47	Lebanese_TurkishJew *{Jordan_Palestinian_Syrian:0.0868 Lebanese_TurkishJew:0.3832}*
			S2	0.53	Turkish *{Turkish:0.4905}*

Table S6. Admixture events inferred by GLOBETROTTER using modern populations (fineSTRUCTURE clusters) and ancient samples as surrogates under the "all donors painting". AdmDate=number of generations (date in years in brackets, using the formula 1950-28*(g+1) that assumes 28 years per generation g of admixture) since the admixture event occurred; S=source number (E1 and E2 indicate two inferred admixture events occurring over approximately the same date interval); "prop" the proportion contributed by each source; "Source composition" indicates the single sampled group that best matches the inferred genetic make-up for the given source, plus in curly brackets a more detailed inference of the genetic make-up of the source (excluding groups inferred to contribute <=5% to this make-up).

Target	AdmDate	AdmDate (min-max)	S	prop	Source composition
Iranian_Zoroastrian	66 (740 CE)	42-89 (570 BCE-746 CE)	S1	0.33	Cypriot *{WC1:0.0542 Cypriot:0.1286 Croatian:0.1373}*
			S2	0.67	WC1 *{WC1:0.6338}*
Iranian_B	18 (1418 CE)	13-26 (1194 CE-1558 CE)	S1	0.39	Pakistani *{Czech:0.0695 Pakistani:0.3205}*
			S2	0.61	Iranian_C *{Iranian_C:0.5884}*
Iranian_C	10 (1642 CE)	6-12 (1586 CE-1754 CE)	S1	0.11	Luhya_Kenya *{Luhya_Kenya:0.0697}*
			S2	0.89	Iranian_B *{Yemeni_B:0.062 Bar8:0.0695 Pathan:0.095 Iranian_B:0.654}*
Indian_Zoroastrian	32 (1026 CE)	19-44 (690 CE-1390 CE)	S1	0.24	Indian_C *{Indian_C:0.24}*
			S2	0.76	WC1 *{Pathan:0.0658 WC1:0.6942}*
Indian_A	49 (550 CE)	29-77 (234 BCE-1110 CE)	S1	0.44	Mala_Vishwabrahmin *{Mala_Vishwabrahmin:0.44}*
			S2	0.56	Indian_Pakistani *{Indian_Pakistani:0.56}*
Indian_B	58 (298 CE)	41-72 (94 BCE-774 CE)	S1	0.24	Bulgarian *{Bulgarian:0.24}*
			S2	0.76	Bengali_Bangladesh *{Indian_C:0.0515 Indian_Pakistani:0.0957 Indian_A:0.1551 Bengali_Bangladesh:0.4577}*
Indian_C	44 (690 CE)	23-68 (18 CE-1278 CE)	S1	0.47	Mala_Vishwabrahmin *{Mala_Vishwabrahmin:0.47}*
			S2	0.53	Indian_B *{Indian_B:0.53}*
Kharia	84 (430 BCE)	60-108 (1102 BCE-242 CE)	S1	0.48	Cambodian *{Mala_Vishwabrahmin:0.0584 Cambodian:0.4216}*
			S2	0.52	Mala_Vishwabrahmin *{Mala_Vishwabrahmin:0.5177}*
Mala_Vishwabrahmin	70 (38 BCE)	43-104 (990 BCE-718 CE)	S1	0.31	UstIshim *{UstIshim:0.264}*
			S2	0.69	Indian_A *{Indian_C:0.1261 Indian_A:0.5639}*
CochinJew_A	29 (1110 CE)	7-40 (802 CE-1726 CE)	S1	0.47	CochinJew_B *{GeorgianJew_IraqiJew:0.0552 CochinJew_B:0.3585}*
			S2	0.53	Iranian_A *{Iranian_A:0.0942 GeorgianJew_IraqiJew:0.1612 Moroccan_Jew:0.2158}*
CochinJew_B	12 (1586CE)	6-21 (1334 CE-1754 CE)	S1	0.46	CochinJew_A *{CochinJew_A:0.46}*
			S2	0.54	Mala_Vishwabrahmin *{Bengali_Bangladesh:0.2617 Mala_Vishwabrahmin:0.2783}*

Indian_Pakistani	23 (1278 CE)	6-40 (802 CE-1754 CE)	S1	0.46	Bengali_Bangladesh *{Indian_B:0.0523 Indian_A:0.0752 Bengali_Bangladesh:0.3269}*
			S2	0.54	Iranian_B *{Balochi:0.1344 Iranian_B:0.1502 Pathan:0.2126}*
Pathan	47 (606 CE)	30-65 (102 CE-1082 CE)	S1	0.43	Turkish *{Turkish_36:0.4299}*
			S2	0.57	Indian_A *{Indian_Pakistani:0.0888 Indian_A:0.4026}*
Hazara	22 (1306 CE)	20-26 (1194 CE-1362 CE)	S1	0.46	Pathan *{Pathan:0.46}*
			S2	0.54	Kalmyk *{Kalmyk:0.54}*
Burusho	47 (606 CE)	35-60 (242 CE-942 CE)	S1	0.49	Bengali_Bangladesh *{Bengali_Bangladesh:0.49}*
			S2	0.51	Tajik_Pomiri *{Tajik_Pomiri:0.51}*
Makrani	16 (1474 CE)	13-20 (1362 CE-1558 CE)	S1	0.06	Luhya_Kenya *{Luhya_Kenya:0.06}*
			S2	0.94	Pakistani *{Iranian_C:0.1993 Pakistani:0.7407}*
Balochi	14 (1530 CE)	7-21 (1334 CE-1726 CE)	S1	0.46	Pakistani *{Brahui:0.0791 Pakistani:0.3807}*
			S2	0.54	Indian_Pakistani *{Brahui:0.0659 Pathan:0.1866 Indian_Pakistani:0.2874}*
Brahui	20 (1362 CE)	13-30 (1082 CE-1558 CE)	S1	0.45	Makrani *{Makrani:0.45}*
			S2	0.55	Balochi *{Balochi:0.55}*
Armenian_Assyrian_ Turkish	61 (214 CE)	36-87 (514 BCE-914 CE)	S1	0.42	Iranian_A *{WC1:0.152 Iranian_A:0.2449}*
			S2	0.58	Lebanese_TurkishJew *{KK1:0.0608 Lebanese_TurkishJew:0.2265 Bar8:0.2639}*
Iranian_A	29 (1110 CE)	23-38 (858 CE-1278 CE)	E1.S1	0.48	WC1 *{Lebanese_TurkishJew:0.0692 WC1:0.379}*
			E1.S2	0.52	Turkish *{Lebanese_TurkishJew:0.0562 Turkish_36:0.4168}*
			E2.S1	0.48	Lebanese_TurkishJew *{Lebanese_TurkishJew:0.48}*
			E2.S2	0.52	Turkish *{WC1:0.094 Turkish:0.426}*
Pakistani	20 (1362 CE)	15-26 (1194 CE-1502 CE)	E1.S1	0.33	Indian_Pakistani *{Indian_Pakistani:0.315}*
			E1.S2	0.67	Makrani *{Brahui_19:0.2274 Makrani:0.4426}*
			E2.S1	0.43	Brahui *{Indian_Pakistani:0.0916 Brahui:0.3384}*
			E2.S2	0.57	Makrani *{Makrani:0.5335}*

Table S7. Admixture events for Indian and Iranian populations inferred by GLOBETROTTER using modern populations (fineSTRUCTURE clusters) and ancient samples as surrogates, under the "non Indian/Iranian donors painting". AdmDate=number of generations (date in years in brackets, using the formula 1950-28*(g+1) that assumes 28 years per generation g of admixture) since the admixture event occurred; S=source number; "prop" the proportion contributed by each source; "Source composition" indicates the single sampled group that best matches the inferred genetic make-up for the given source, plus in curly brackets a more detailed inference of the genetic make-up of the source (excluding groups inferred to contribute <=5% to this make-up).

Target	AdmDate	AdmDate (min-max)	S	Prop	Source composition
IranianZoroastrian	56 (354 CE)	16-94 (710 BCE-1474 CE)	S1	0.48	Greek_Coriell *{Greek_Coriell:0.4078}*
			S2	0.52	WC1 *{Pathan:0.0748 IndianZoroastrian:0.1909 WC1:0.254}*
Iranian_A	25 (1222 CE)	21-32 (1026 CE-1334 CE)	S1	0.43	Italian_EastSicilian *{Lebanese_TurkishJew:0.0984 Italian_EastSicilian:0.2987}*
			S2	0.57	IndianZoroastrian *{WC1:0.0634 IndianZoroastrian:0.5066}*
Iranian_B	18 (1418 CE)	13-25 (1222 CE-1558 CE)	S1	0.45	Balochi *{Balochi:0.45}*
			S2	0.55	Iranian_C *{Iranian_C:0.55}*
Iranian_C	10 (1642 CE)	7-13 (1558 CE-1726 CE)	S1	0.09	Tswana *{Tswana:0.0894}*
			S2	0.91	Iranian_B *{Italian_WestSicilian:0.0578 Yemeni:0.0746 Pathan:0.0972 Iranian_B:0.6112}*
IndianZoroastrian	30 (1082 CE)	21-45 (662 CE-1334 CE)	S1	0.39	Indian_C *{Indian_C:0.39}*
			S2	0.61	IranianZoroastrian *{Jordan_Palestinian_Syrian:0.0574 Cypriot:0.0589 Armenian_Assyrian_Turkish:0.0613 Greek_Coriell:0.104 IranianZoroastrian:0.2962}*
Indian_A	63 (158 CE)	38-89 (570 BCE-858 CE)	S1	0.48	Pathan *{Pathan:0.48}*
			S2	0.52	Bengali_Bangladesh_BEB *{Bengali_Bangladesh_BEB:0.52}*
Indian_B	58 (298 CE)	43-81 (346 BCE-718 CE)	S1	0.19	French *{French:0.1885}*
			S2	0.81	Indian_C *{Indian_A:0.3323 Indian_C:0.4777}*
Indian_C	69 (10 BCE)	51-89 (570 BCE-494 CE)	S1	0.47	Pathan *{Pathan:0.47}*
			S2	0.53	Mala_Vishwabrahmin *{Mala_Vishwabrahmin:0.53}*
CochinJew_A	9 (1670 CE)	5-15 (1502 CE-1782	S1	0.37	KuchinJew_B *{KuchinJew_B:0.0547 UstIshim:0.1107 Indian_A:0.1577}*

		CE)	S2	0.63	GeorgianJew_IraqiJew *{Indian_A:0.0712 WC1:0.0995 Moroccan_Jew:0.2227 GeorgianJew_IraqiJew_16:0.2234}*
CochinJew_B	19 (1390 CE)	12-33 (998 CE -1586 CE)	S1	0.47	WC1 *{WC1:0.47}*
			S2	0.53	Mala_Vishwabrahmin *{Mala_Vishwabrahmin:0.53}*
Mala_Vishwabrahmin	66 (74 CE)	43-82 (374 BCE-718 CE)	S1	0.32	Onge *{Onge_11:0.32}*
			S2	0.68	Indian_C *{KuchinJew_B:0.0864 Pathan:0.2798 Indian_C:0.3138}*
Kharia	89 (570 BCE)	67-122 (1484 BCE -46 CE)	S1	0.39	Cambodian *{Cambodian:0.39}*
			S2	0.61	Mala_Vishwabrahmin *{Mala_Vishwabrahmin:0.61}*

Table S8. Admixture events inferred by GLOBETROTTER for single outlier Iranian Zoroastrians using all modern populations (fineSTRUCTURE clusters) as surrogates under the "all donors painting", also including results for the groups they cluster with (i.e. Iranian_B, which includes YZ020 and Lebanese_TurkishJew, which includes YZ024). Only results for null.ind 1 analyses are shown. AdmDate=number of generations (date in years in brackets, using the formula 1950-28*(g+1) that assumes 28 years per generation g of admixture) since the admixture event occurred; S=source number; "prop" the proportion contributed by each source; "Source composition" indicates the single sampled group that best matches the inferred genetic make-up for the given source, plus in curly brackets a more detailed inference of the genetic make-up of the source (excluding groups inferred to contribute <=5% to this make-up).

Target	AdmDate	S	prop	Source composition
**Iranian_Zoroastrian_YZ020**	18 (1418 CE)	S1	0.23	Makrani *{Makrani:0.1117 Pakistani:0.1057}*
		S2	0.77	Iranian_A *{Iranian_A: 0.3157 Lebanese_TurkishJew: 0.1266 Brahui: 0.0540}*
**Iranian_B**	18	S1	0.38	Pakistani {*Iranian_A:0.0656 Pakistani:0.3144*}
	(1418 CE)		0.62	Iranian_C {*Iranian_C:0.62*}
**Iranian_Zoroastrian_YZ024**	88 (542 BCE)	S1	0.01	Mozabite
		S2	0.99	Ashkenazi_Jew *{Ashkenazi_Jew:0.989}*
**Lebanese_TurkishJew**	Date1: 13 (1558 CE)	S1	0.03	Yemeni_B_2
		S2	0.97	Turkish {*Ashkenazi_Jew: 0.065 Greek_Coriell: 0.078 Cypriot: 0.092 Jordan_Palestinian_Syrian: 0.103 Italian_EastSicilian: 0.115 Iranian_A:0.206*}
	Date2: 57 (326 CE)	S1	0.06	Masai_Ayodo
		S2	0.94	Turkish{*Armenian_Assyrian_Turkish:0.057435134674136 Cypriot:0.056 Italian_Bergamo: 0.056 Greek_Coriell: 0.069 Jordan_Palestinian_Syrian: 0.074 Turkish: 0.089 Iranian_A:0.248*}

Table S9. Y-chromosome haplogroup frequencies and gene diversities.

Haplogroup	Iranian Non Zoroastrian	Indian Non Zoroastrian	Zoroastrian - India		Zoroastrian - Iran	
			Lay	Priest	Lay	Priest
**P*(xR1a1a)**	0.169	0.122	0.238	0.310	0.171	0.125
**N1c1**	0.012	0.000	0.000	0.000	0.000	0.000
**BT*(xDE,JT)**	0.140	0.268	0.057	0.085	0.053	0.000
**E*(xE1b1a1)**	0.110	0.000	0.057	0.014	0.118	0.000
**K*(xL,N1c,O1b2,P)**	0.041	0.049	0.016	0.000	0.039	0.250
**L**	0.047	0.098	0.025	0.549	0.013	0.000
**R1a1a**	0.105	0.317	0.057	0.000	0.053	0.125
**J**	0.378	0.146	0.549	0.042	0.553	0.500
**Total N**	172	41	122	71	76	8
**H** (SE)	0.787 (0.020)	0.799 (0.032)	0.636 (0.038)	0.602 (0.042)	0.653 (0.051)	0.750 (0.139)

Table S10. MtDNA macrohaplogroup frequencies and gene diversities.

Macro-haplogroups	Iranian Non Zoroastrian	Indian Non Zoroastrian	Zoroastrian – India		Zoroastrian – Iran	
			Lay	Priest	Lay	Priest
**A**	0.005	0.000	0.000	0.000	0.000	0.000
**B**	0.026	0.000	0.000	0.000	0.000	0.000
**C**	0.005	0.000	0.000	0.000	0.000	0.000
**D**	0.005	0.022	0.008	0.028	0.000	0.000
**H**	0.290	0.196	0.223	0.197	0.291	0.250
**I**	0.031	0.000	0.000	0.000	0.000	0.000
**J**	0.130	0.022	0.008	0.000	0.000	0.000
**K**	0.078	0.000	0.000	0.000	0.000	0.000
**L**	0.000	0.043	0.083	0.056	0.013	0.000
**M**	0.026	0.391	0.430	0.549	0.013	0.000
**N**	0.021	0.087	0.008	0.000	0.051	0.125
**P**	0.010	0.000	0.000	0.000	0.000	0.000
**R**	0.026	0.065	0.066	0.042	0.000	0.000
**S**	0.005	0.000	0.000	0.000	0.000	0.000
**T**	0.073	0.000	0.050	0.014	0.532	0.625
**U**	0.202	0.109	0.107	0.113	0.051	0.000
**W**	0.036	0.065	0.000	0.000	0.000	0.000
**X**	0.026	0.000	0.017	0.000	0.051	0.000
**Y**	0.005	0.000	0.000	0.000	0.000	0.000
**Total N**	193	46	121	71	79	8
**H (SE)**	0.676 (0.059)	0.819 (0.022)	0.661 (0.036)	0.581 (0.054)	0.674 (0.044)	0.821 (0.1)

Table S11. XPEHH results for Indian Zoroastrians vs Indian non-Zoroastrians. SNPs below and above quantiles 0.0001 and 0.9999 of the empirical distribution respectively (see methods), including the genes within those regions, or the flanking genes in the case of intergenic SNPs.

SNP	Chr	BP	Location	Gene	XPEHH
rs4559034	chr5	117451628	ncRNA_intronic	*LOC102467224*	-4.41826
rs972264	chr5	117527579	ncRNA_intronic	*LOC102467224*	-4.41874
rs17432160	chr5	117528933	ncRNA_intronic	*LOC102467224*	-4.65247
rs2061883	chr5	117530755	ncRNA_intronic	*LOC102467224*	-4.59379
rs2061882	chr5	117531223	ncRNA_intronic	*LOC102467224*	-4.59379
rs28566849	chr5	117533566	ncRNA_intronic	*LOC102467224*	-4.59379
rs1382704	chr5	117534903	ncRNA_intronic	*LOC102467224*	-4.59415
rs1479180	chr5	117538740	ncRNA_intronic	*LOC102467224*	-4.59415
rs34206135	chr5	117549966	ncRNA_intronic	*LOC102467224*	-4.59174
rs6883098	chr5	117560263	ncRNA_intronic	*LOC102467224*	-4.81066
rs11955483	chr5	117561687	ncRNA_intronic	*LOC102467224*	-5.12559
rs11744859	chr5	117569374	ncRNA_intronic	*LOC102467224*	-4.79995
rs11748941	chr5	117570688	ncRNA_intronic	*LOC102467224*	-4.54282
rs61250898	chr5	117575717	ncRNA_intronic	*LOC102467224*	-4.46385
rs77543824	chr5	117579383	ncRNA_intronic	*LOC102467224*	-4.46385
rs11216547	chr11	117666413	intronic	*DSCAML1*	-4.50502
rs10894845	chr11	134448470	intergenic	*LOC283177*	-4.63119
rs9919607	chr11	134449247	intergenic	*LOC283177*	-4.60421
rs7926027	chr11	134466656	intergenic	*LOC283177*	-4.68149
rs3019685	chr11	134485352	intergenic	*LOC283177*	-5.61552
rs2000858	chr11	134485458	intergenic	*LOC283177*	-5.41945
rs2097112	chr11	134486859	intergenic	*LOC283177*	-5.34052
rs2187463	chr11	134493110	intergenic	*LOC283177*	-5.52537
rs3017983	chr11	134493977	intergenic	*LOC283177*	-5.57282
rs3019668	chr11	134495874	intergenic	*LOC283177*	-4.95463
rs1944878	chr11	134498120	intergenic	*LOC283177*	-4.8481
rs3017965	chr11	134506955	intergenic	*LOC283177*	-5.04921
rs7939984	chr11	134507055	intergenic	*LOC283177*	-5.11849
rs3017963	chr11	134508438	intergenic	*LOC283177*	-5.07565
rs3019652	chr11	134508966	intergenic	*LOC283177*	-4.83669
rs113043921	chr11	134512512	intergenic	*LOC283177*	-4.83669
rs3019659	chr11	134513448	intergenic	*LOC283177*	-4.64051
rs949107	chr11	134536000	intergenic	*LOC283177*	-4.47438
rs11601492	chr11	134539450	intergenic	*LOC283177*	-4.54736
rs3017995	chr11	134539716	intergenic	*LOC283177*	-4.56322
rs1939728	chr11	134547554	intergenic	*LOC283177*	-4.538
rs6086704	chr20	947150	intronic	*RSPO4*	-4.42287

rs2223962	chr20	952218	intronic	*RSPO4*	-4.38887
rs4297887	chr2	115887977	intronic	*DPP10*	4.5017
rs35003803	chr4	32955931	intergenic	*LOC102723828,LOC101928622*	4.84255
rs1400145	chr12	73306993	intergenic	*TRHDE,LOC101928137*	4.44321
rs11179461	chr12	73317554	intergenic	*TRHDE,LOC101928137*	4.41985
rs12306579	chr12	73317695	intergenic	*TRHDE,LOC101928137*	4.41985
rs113062692	chr12	73321006	intergenic	*TRHDE,LOC101928137*	4.41985
rs2057130	chr14	64961479	intronic	*ZBTB25*	4.40508
rs9939675	chr16	78398705	intronic	*WWOX*	4.81195
rs4888787	chr16	78399140	intronic	*WWOX*	4.68308
rs112927307	chr16	78399322	intronic	*WWOX*	4.68308
rs72796072	chr16	78416119	intronic	*WWOX*	4.46482
rs72796083	chr16	78419515	intronic	*WWOX*	4.46482
rs2667545	chr16	78502389	intronic	*WWOX*	4.51242
rs3115955	chr16	78503595	intronic	*WWOX*	4.51242
rs12598729	chr16	78504236	intronic	*WWOX*	4.54594
rs2738680	chr16	78505408	intronic	*WWOX*	4.58225
rs2738681	chr16	78505709	intronic	*WWOX*	4.55812
rs73574998	chr16	78506089	intronic	*WWOX*	4.55812
rs8051225	chr16	78512313	intronic	*WWOX*	5.28383
rs11643648	chr16	78514583	intronic	*WWOX*	5.28383
rs2667562	chr16	78515158	intronic	*WWOX*	5.28383
rs2667569	chr16	78517377	intronic	*WWOX*	5.28383
rs2667570	chr16	78517684	intronic	*WWOX*	5.28383
rs2345998	chr16	78519416	intronic	*WWOX*	5.28383
rs1540757	chr16	78519689	intronic	*WWOX*	5.12212
rs2667579	chr16	78521339	intronic	*WWOX*	5.12212
rs2738700	chr16	78521500	intronic	*WWOX*	5.12212
rs2738701	chr16	78521997	intronic	*WWOX*	5.12212
rs2738704	chr16	78522184	intronic	*WWOX*	5.12212
rs28505640	chr16	78525030	intronic	*WWOX*	5.12212
rs2738714	chr16	78525954	intronic	*WWOX*	5.12212
rs62036391	chr16	78526959	intronic	*WWOX*	5.12212
rs2738721	chr16	78528446	intronic	*WWOX*	5.05292
rs2738727	chr16	78530734	intronic	*WWOX*	5.05292
rs2667589	chr16	78530885	intronic	*WWOX*	5.05292
rs17639042	chr16	78533357	intronic	*WWOX*	4.52346
rs7205635	chr16	78536121	intronic	*WWOX*	4.52346
rs12443611	chr16	78537146	intronic	*WWOX*	5.18486
rs1877281	chr16	78539415	intronic	*WWOX*	4.57852
rs1882958	chr16	78541355	intronic	*WWOX*	4.57852
rs58211226	chr16	78542655	intronic	*WWOX*	4.57852

rs11645676	chr16	78563832	intronic	*WWOX*	4.45776
rs2738498	chr16	78568270	intronic	*WWOX*	4.38329
rs9635580	chr16	78572354	intronic	*WWOX*	4.39638
rs1883519	chr20	44130596	intergenic	*WFDC2,SPINT3*	4.67317
rs6032249	chr20	44131517	intergenic	*WFDC2,SPINT3*	4.6697
rs6032250	chr20	44131736	intergenic	*WFDC2,SPINT3*	4.6697
rs1546889	chr20	44132906	intergenic	*WFDC2,SPINT3*	4.6697
rs909878	chr20	44135521	intergenic	*WFDC2,SPINT3*	4.59175
rs8124864	chr20	44144478	upstream	*SPINT3*	4.67909
rs6017595	chr20	44154532	intergenic	*SPINT3,WFDC6*	4.69295
rs146198026	chr20	44160213	intergenic	*SPINT3,WFDC6*	4.69295
rs3746593	chr20	44162849	UTR3	*WFDC6*	4.69295
rs6032274	chr20	44163988	intronic	*WFDC6*	4.75832
rs6094159	chr20	44164091	intronic	*WFDC6*	4.5579
rs6032331	chr20	44196310	intronic	*WFDC8*	4.66286
rs6104229	chr20	44197259	intronic	*WFDC8*	4.39378
rs6017628	chr20	44204536	intronic	*WFDC8*	4.39378
rs3091718	chr20	44216791	intergenic	*WFDC8,WFDC9*	4.39378
rs3091929	chr20	44216911	intergenic	*WFDC8,WFDC9*	4.70231
rs4812922	chr20	44217726	intergenic	*WFDC8,WFDC9*	4.70231
rs75511654	chr20	44218160	intergenic	*WFDC8,WFDC9*	4.70231
rs2425707	chr20	44224978	intergenic	*WFDC8,WFDC9*	5.04173
rs6032368	chr20	44225010	intergenic	*WFDC8,WFDC9*	5.04173
rs2425708	chr20	44226454	intergenic	*WFDC8,WFDC9*	5.20786
rs2425710	chr20	44227493	intergenic	*WFDC8,WFDC9*	5.03559
rs112947150	chr20	44227993	intergenic	*WFDC8,WFDC9*	5.03559
rs73131022	chr20	44228451	intergenic	*WFDC8,WFDC9*	5.03559
rs2235600	chr20	44238299	intronic	*WFDC9*	4.84654
rs978778	chr20	44243376	intronic	*WFDC9*	4.84654
rs1487327	chr20	44247986	intronic	*WFDC9*	5.01467
rs76995892	chr20	44251340	intronic	*WFDC9*	5.01467
rs3091694	chr20	44251629	intronic	*WFDC9*	5.17645
rs1157672	chr20	44258743	intronic	*WFDC10A,WFDC9*	5.17645
rs2272961	chr20	44259549	exonic	*WFDC10A*	4.9251
rs78065887	chr20	44278320	intronic	*WFDC11*	4.9251
rs4810461	chr20	44278445	intronic	*WFDC11*	4.93388
rs1487318	chr20	44295811	intronic	*WFDC11*	4.96159
rs6073854	chr20	44309605	intergenic	*WFDC11,WFDC10B*	4.89063
rs1013562	chr20	44310744	intergenic	*WFDC11,WFDC10B*	4.85908
rs6065861	chr20	44311056	intergenic	*WFDC11,WFDC10B*	4.85908
rs4810465	chr20	44311354	intergenic	*WFDC11,WFDC10B*	4.85908
rs2281211	chr20	44312858	downstream	*WFDC10B*	4.85908

rs2072974	chr20	44313401	UTR3	*WFDC10B*	4.85908
rs67538107	chr20	44327802	intronic	*WFDC10B*	5.01164
rs6073862	chr20	44331705	intronic	*WFDC10B,WFDC13*	4.62491
rs386196	chr20	44332873	intronic	*WFDC10B,WFDC13*	4.62491
rs6032449	chr20	44336716	UTR3	*WFDC13*	4.73825
rs6017653	chr20	44336924	UTR3	*WFDC13*	4.92872
rs232263	chr20	44350728	upstream	*SPINT4*	4.87487
rs11697000	chr20	44350763	upstream	*SPINT4*	4.87487
rs1386505	chr20	44351421	intronic	*SPINT4*	5.31282
rs1386504	chr20	44351515	intronic	*SPINT4*	5.21524
rs2200586	chr20	44351554	intronic	*SPINT4*	5.51207
rs6017666	chr20	44352307	intronic	*SPINT4*	5.18131
rs73908195	chr20	44353900	intronic	*SPINT4*	5.18131
rs761810	chr20	44355180	downstream	*SPINT4*	5.18131
rs232258	chr20	44356194	intergenic	*SPINT4,WFDC3*	5.20808
rs462038	chr20	44360467	intergenic	*SPINT4,WFDC3*	5.07953
rs459548	chr20	44366500	intergenic	*SPINT4,WFDC3*	5.00341
rs463793	chr20	44368325	intergenic	*SPINT4,WFDC3*	5.00341
rs455451	chr20	44373893	intergenic	*SPINT4,WFDC3*	5.00341
rs382515	chr20	44380316	intergenic	*SPINT4,WFDC3*	5.00341
rs405247	chr20	44380347	intergenic	*SPINT4,WFDC3*	5.00341
rs454874	chr20	44380471	intergenic	*SPINT4,WFDC3*	5.00341
rs17365711	chr20	44395664	intergenic	*SPINT4,WFDC3*	5.00341
rs2664529	chr20	44402869	UTR3	*WFDC3*	5.06458
rs6130930	chr20	44410837	intronic	*WFDC3*	5.24862
rs7263437	chr20	44410887	intronic	*WFDC3*	5.24862
rs567348	chr20	44410933	intronic	*WFDC3*	5.23038
rs3746493	chr20	44418564	exonic	*WFDC3*	5.21809
rs12480813	chr20	44425776	intronic	*DNTTIP1*	5.21809
rs4812964	chr20	44435334	intronic	*DNTTIP1*	5.21809
rs399672	chr20	44438299	intronic	*DNTTIP1*	5.25915
rs6104355	chr20	44445676	downstream	*UBE2C*	5.23641
rs145338460	chr20	44446276	downstream	*UBE2C*	5.23641
rs80177750	chr20	44448315	intergenic	*UBE2C,TNNC2*	5.23641
rs4629	chr20	44452697	exonic	*TNNC2*	5.09931
rs437122	chr20	44454978	intronic	*TNNC2*	5.32993
rs116367119	chr20	44455890	UTR5	*TNNC2*	5.32993
rs58751125	chr20	44466211	intronic	*SNX21*	5.32993
rs58334571	chr20	44468894	intronic	*SNX21*	5.32993
rs1057275	chr20	44471276	UTR3	*SNX21*	5.32993
rs73291275	chr20	44477528	intronic	*ACOT8*	5.32993
rs3746495	chr20	44479794	intronic	*ACOT8*	5.32993

rs6124749	chr20	44481871	intronic	*ACOT8*	5.10242
rs1967656	chr20	44488199	intronic	*ZSWIM3*	5.10242
rs6104374	chr20	44498391	intronic	*ZSWIM3*	5.08311
rs78044163	chr20	44503454	intronic	*ZSWIM3*	5.08311
rs2903808	chr20	44505973	exonic	*ZSWIM3*	5.06791
rs3746525	chr20	44507385	UTR3	*ZSWIM3*	5.06791
rs3746524	chr20	44507502	UTR3	*ZSWIM3*	5.06791
rs742034	chr20	44522005	intronic	*CTSA*	5.09269
rs4810476	chr20	44522594	intronic	*CTSA*	5.01164
rs115172363	chr20	44527765	intronic	*PLTP*	5.01164
rs394643	chr20	44540178	intronic	*PLTP*	4.52057
rs4810479	chr20	44545048	intergenic	*PLTP,PCIF1*	4.96894

Table S12. XPEHH results for Iranian Zoroastrians vs Iranian non-Zoroastrians. SNPs below and above quantiles 0.0001 and 0.9999 of the empirical distribution respectively (see methods), including the genes within those regions, or the flanking genes in the case of intergenic SNPs.

SNP	Chr	BP	Location	Gene	XPEHH
rs3857976	chr8	84341097	intergenic	*LINC01419,RALYL*	-4.57514
rs2053744	chr8	84374321	intergenic	*LINC01419,RALYL*	-4.47158
rs73282932	chr8	84382174	intergenic	*LINC01419,RALYL*	-4.55186
rs13253167	chr8	84425132	intergenic	*LINC01419,RALYL*	-4.58144
rs10817815	chr9	118451020	intergenic	*DEC1,LOC101928775*	-4.93139
rs72764742	chr9	118452085	intergenic	*DEC1,LOC101928775*	-4.79559
rs73654588	chr9	118453824	intergenic	*DEC1,LOC101928775*	-4.81785
rs7863684	chr9	118455827	intergenic	*DEC1,LOC101928775*	-4.6041
rs10982901	chr9	118499474	intergenic	*DEC1,LOC101928775*	-4.52498
rs4978653	chr9	118500105	intergenic	*DEC1,LOC101928775*	-4.52498
rs384626	chr10	61136215	intergenic	*FAM13C,SLC16A9*	-4.51628
rs78451775	chr10	61136963	intergenic	*FAM13C,SLC16A9*	-4.51628
rs11006458	chr10	61139869	intergenic	*FAM13C,SLC16A9*	-5.03629
rs513817	chr10	61140376	intergenic	*FAM13C,SLC16A9*	-4.97856
rs397014	chr10	61142485	intergenic	*FAM13C,SLC16A9*	-4.78198
rs7095696	chr10	85076518	intergenic	*NRG3,GHITM*	-4.47023
rs10886214	chr10	85127739	intergenic	*NRG3,GHITM*	-4.4687
rs10749261	chr10	85131723	intergenic	*NRG3,GHITM*	-4.48617
rs76706594	chr10	85134722	intergenic	*NRG3,GHITM*	-4.48617
rs141563527	chr10	85135411	intergenic	*NRG3,GHITM*	-4.48617
rs4800511	chr18	21360740	intronic	*LAMA3*	-4.52881
rs1112378	chr18	21384324	intronic	*LAMA3*	-4.52881
rs10497759	chr2	196277506	intergenic	*LOC101927431,SLC39A10*	4.51925
rs11692599	chr2	196278883	intergenic	*LOC101927431,SLC39A10*	4.51925
rs7559362	chr2	196319300	intergenic	*LOC101927431,SLC39A10*	5.04777
rs4850618	chr2	196321390	intergenic	*LOC101927431,SLC39A10*	5.06592
rs12693770	chr2	196326146	intergenic	*LOC101927431,SLC39A10*	5.32369
rs10497775	chr2	196326799	intergenic	*LOC101927431,SLC39A10*	5.27907
rs1500604	chr2	196330222	intergenic	*LOC101927431,SLC39A10*	5.40105
rs74532088	chr2	196332769	intergenic	*LOC101927431,SLC39A10*	5.17855
rs4850619	chr2	196333358	intergenic	*LOC101927431,SLC39A10*	5.11385
rs6434780	chr2	196335282	intergenic	*LOC101927431,SLC39A10*	5.08859
rs2720162	chr2	231629360	intronic	*CAB39*	4.49913
rs192998154	chr2	231631709	intronic	*CAB39*	4.49913
rs1322826	chr6	10082402	intergenic	*LOC100506207,TFAP2A*	4.62267

rs10277232	chr7	157959620	intronic	*PTPRN2*	4.91094
rs8020506	chr14	24345479	intergenic	*DHRS2,DHRS4-AS1*	4.63184
rs10139869	chr14	24346429	intergenic	*DHRS2,DHRS4-AS1*	4.54983
rs111706690	chr14	24352813	intergenic	*DHRS2,DHRS4-AS1*	4.54983
rs74036713	chr14	24354181	intergenic	*DHRS2,DHRS4-AS1*	4.54983
rs11158422	chr14	24370428	intergenic	*DHRS2,DHRS4-AS1*	4.58538
rs1957684	chr14	24375437	intergenic	*DHRS2,DHRS4-AS1*	4.52319
rs12444419	chr16	71408475	intronic	*CALB2*	4.97967
rs8046337	chr16	71408527	intronic	*CALB2*	4.97967
rs6499515	chr16	71408940	intronic	*CALB2*	4.98128
rs80317611	chr16	71412682	intronic	*CALB2*	4.85094
rs9940982	chr16	71415585	intronic	*CALB2*	4.9219
rs7200566	chr16	71426563	intergenic	*CALB2,ZNF23*	4.9219
rs35326792	chr16	71427061	intergenic	*CALB2,ZNF23*	5.01915
rs12445377	chr16	71428601	intergenic	*CALB2,ZNF23*	5.01915
rs891131	chr16	71432417	intergenic	*CALB2,ZNF23*	5.01915
rs2839619	chr21	44436177	intronic	*PKNOX1*	4.50955
rs3737434	chr21	44437142	intronic	*PKNOX1*	4.64333
rs2839624	chr21	44446030	intronic	*PKNOX1*	4.75923
rs2839625	chr21	44447347	intronic	*PKNOX1*	4.82339
rs2839626	chr21	44448384	intronic	*PKNOX1*	4.80037
rs2839627	chr21	44448718	intronic	*PKNOX1*	4.77703
rs234729	chr21	44449095	intronic	*PKNOX1*	4.77703
