# Supplementary material for: The Genetic Legacy of Zoroastrianism in Iran and India: Insights into Population Structure, Gene Flow, and Selection
Source: Am J Hum Genet. 2017 Aug 24;101(3):353–68. doi: 10.1016/j.ajhg.2017.07.013 (PMC5590844; doi:10.1016/j.ajhg.2017.07.013)
Supplement: Document S2. Article plus Supplemental Data [file mmc4.pdf]

The Genetic Legacy of Zoroastrianism in Iran and India: Insights into Population Structure, Gene Flow, and Selection

Saioa López,[1,11,12,*] Mark G. Thomas,[1,11] Lucy van Dorp,[1,2] Naser Ansari-Pour,[3] Sarah Stewart,[4] Abigail L. Jones,[5] Erik Jelinek,[1] Lounès Chikhi,[6,7] Tudor Parfitt,[8] Neil Bradman,[9] Michael E. Weale,[10] and Garrett Hellenthal[1,*]

Zoroastrianism is one of the oldest extant religions in the world, originating in Persia (present-day Iran) during the second millennium BCE. Historical records indicate that migrants from Persia brought Zoroastrianism to India, but there is debate over the timing of these migrations. Here we present genome-wide autosomal, Y chromosome, and mitochondrial DNA data from Iranian and Indian Zoroastrians and neighboring modern-day Indian and Iranian populations and conduct a comprehensive genome-wide genetic analysis in these groups. Using powerful haplotype-based techniques, we find that Zoroastrians in Iran and India have increased genetic homogeneity relative to other sampled groups in their respective countries, consistent with their current practices of endogamy. Despite this, we infer that Indian Zoroastrians (Parsis) intermixed with local groups sometime after their arrival in India, dating this mixture to 690–1390 CE and providing strong evidence that Iranian Zoroastrian ancestry was maintained primarily through the male line. By making use of the rich information in DNA from ancient human remains, we also highlight admixture in the ancestors of Iranian Zoroastrians dated to 570 BCE–746 CE, older than admixture seen in any other sampled Iranian group, consistent with a long-standing isolation of Zoroastrians from outside groups. Finally, we report results, and challenges, from a genome-wide scan to identify genomic regions showing signatures of positive selection in present-day Zoroastrians that might correlate to the prevalence of particular diseases among these communities.

Introduction

Zoroastrianism developed from an ancient religion that was once shared by the ancestors of tribes that settled in Iran and northern India and is thought to have been founded by the prophet priest Zarathustra (Zoroaster in Greek). Since there is no context or documentation for the life of Zarathustra, his very existence is a matter for debate. Although some scholars have proposed that he lived in the 6th century BCE, i.e., during the Achaemenic period, most scholars now believe he lived around 1200 BCE, at a time when the ancient Iranians inhabited the areas of the Inner Asian Steppes (also a subject of great controversy[1,2]) prior to the great migrations south to modern Iran, Afghanistan, Northern Iraq, and parts of Central Asia.[3] Zoroastrianism became the state religion of three great Iranian empires: Achaemenid (559–330 BCE) founded by King Cyrus the Great and ended by the conquest of Alexander the Great, Parthian (c. 247 BCE–224 CE), and Sasanian (224–651 CE), during which time the religion as an imperial faith is best known. Zoroastrianism ceased to be the state religion of Iran after the Arab conquests (633–654 CE), although it is thought that widespread conversion to Islam did not begin until about 767 CE.[4]

According to Parsi (i.e., Indian Zoroastrians) tradition, a group of Zoroastrians set sail from Iran to escape persecution by the Muslim majority. They landed on the coast of Gujarat (India) where they were permitted to stay and practice their religion. The date of the arrival has been the cause of speculation and varies between 785 CE[5] and 936 CE.[6] These dates, among others, are based on the *Qisseh-ye Sanjan*, a legendary account of the journey by sea from Iran and settlement in India.[7] However, maritime trade is known to have taken place between ethnic groups from Iran, including Zoroastrians, and peoples in India long before the arrival of Islam.[8] Down the subsequent centuries, the Indian Zoroastrians maintained contact with the Zoroastrians of Iran and later became an influential minority under British Colonial rule.

Zoroastrian communities today (2011 census) are concentrated in India (61,000 people), Southern Pakistan (1,675), and Iran—mainly in Tehran, Yazd, and Kerman—(14,000). In the last 200 years Zoroastrians, both Parsi and Irani, have formed diaspora communities in North America (14,306), Canada (6,422), Britain (5,000), Australasia (3,808), and the Middle East (2,030).

[1]Department Genetics, Evolution & Environment, University College London, London WC1E 6BT, UK; [2]Centre for Mathematics and Physics in the Life Sciences and Experimental Biology (CoMPLEX), University College London, London WC1E 6BT, UK; [3]Faculty of New Sciences and Technology, University of Tehran, Tehran 14395-1561, Iran; [4]SOAS, University of London, London WC1H 0XG, UK; [5]Liverpool Women's Hospital, Liverpool L8 7SS, UK; [6]CNRS, Université Paul Sabatier, Toulouse 31062, France; [7]Instituto Gulbenkian de Ciência, Oeiras 2780-156, Portugal; [8]Florida International University, Miami, FL 33199, USA; [9]Henry Stewart Group, London WC1A 2HN, UK; [10]Department Medical & Molecular Genetics, King's College London, London SE1 9RT, UK
[11]These authors contributed equally to this work
[12]Twitter: @Saioa_l
*Correspondence: saioa.lopez@ucl.ac.uk (S.L.), g.hellenthal@ucl.ac.uk (G.H.)
http://dx.doi.org/10.1016/j.ajhg.2017.07.013.
© 2017 The Authors. This is an open access article under the CC BY license (http://creativecommons.org/licenses/by/4.0/).

Zoroastrianism is a non-proselytizing religion, with a hereditary male priesthood of uncertain origins.[9] Among the Parsis, priestly families are distinguished from the laity. Priestly status is patrilineal, although there is also a strong matrilineal component with the daughters of priests encouraged to marry into priestly families. Remarkably, many priests preserve family genealogies that can be traced back to the purported time of arrival of Iranian Zoroastrians in India and beyond to an Iranian homeland.

Genetic data provide a means of examining the biological relationships of different populations and testing claims of common ancestry. Previous studies of Iranian Zoroastrians have suggested that they are genetically differentiated from their neighboring populations. For example, Farjadian et al.[10] analyzed mitochondrial DNA (mtDNA) variation in 14 different ethnic groups from Iran and observed that Zoroastrians and Jews were genetically distinct from other groups. In the same vein, Lashgary et al.[11] analyzed 14 bi-allelic loci from the non-recombining region of the Y chromosome (NRY) and observed a notable reduction in haplogroup diversity in Iranian Zoroastrians compared with all other groups. Furthermore, a recent study using genome-wide autosomal DNA found that haplotype patterns in Iranian Zoroastrians matched more than other modern Iranian groups to a high-coverage early Neolithic farmer genome from Iran.[12]

Less is known about the genetic landscape and the origins of Zoroastrianism in India, despite Parsis representing more than 80% of present-day Zoroastrians worldwide.[13] A study of four restriction fragment length polymorphisms (RFLPs) suggested a closer genetic affinity of Parsis to Southern Europeans than to non-Parsis from Bombay.[14] Furthermore, NRY haplotype analysis[15] and patterns of variation at the HLA locus[16] in the Parsis of Pakistan support a predominately Iranian origin.

Prompted by these observations, we explored the genetic legacy of Zoroastrianism in more detail by generating genome-wide autosomal and Y/mtDNA genotype data for Iranian and Indian Zoroastrian individuals and comparing them to other publicly available genetic data. With this study we aimed to address the following questions.

1. Which demographic processes (e.g., admixture, isolation) have contributed most to present-day genetic differences between Zoroastrian and non-Zoroastrian groups sampled from the same country?
2. Is there evidence of increased genetic homogeneity in Zoroastrian groups relative to non-Zoroastrian groups?
3. Is there genetic evidence for admixture events in Zoroastrian groups? Does this support historical records tracing the origin of Indian Zoroastrians (a.k.a. Parsis) to migrants from Persia?
4. Is there evidence that any inferred admixture events were sex biased?
5. Is there genetic evidence for the patrilineal descent of Parsi priests from a small number of founding individuals?
6. Using a genome-wide scan, can we reliably detect genomic signatures of positive selection in the Zoroastrian populations that may relate to the prevalence of diseases or other phenotypic traits in the community?

Subjects and Methods

Samples

Buccal swabs were collected from a total of 526 men from India, Iran, the United Arab Emirates, and the United Kingdom (see Table S1). Individuals sampled in the United Arab Emirates are mainly first-generation Parsis who left Aden following the communist coup in 1970, after which Asians were expelled (Aden was part of the Bombay Presidency until 1947 and the British left Aden in 1967–1968). Individuals sampled from the United Kingdom Zoroastrian population are mainly descendants of 19th century immigrants; the Zoroastrian Association was formed in 1861, at which time there were around 50 Zoroastrians living in the UK.[17] Swabs were stored in a DNA preservative solution containing 0.5% sodium doecyl sulfate and 0.05 M EDTA for transport purposes and DNA was purified by phenol-chloroform extraction/isopropanol precipitation. Work on the genetics of these samples is covered by the UK ethics committee London Bentham REC (formally the Joint UCL/UCLH Committees on the Ethics of Human Research: Committee A and Alpha, REC reference number 99/0196, Chief Investigator M.G.T.). The procedures followed were in accordance with the ethical standards of London Bentham REC and Zoroastrian Studies (Bombay). Informed consent was obtained from all individuals before samples were taken.

Genome-wide Genotyping with the Human Origins Array

71 of these individuals (29 Iranian Zoroastrians, 17 Iranian Farsi, 13 Indian Zoroastrians, and 12 Indian Hindu), all belonging to the lay (i.e., non-priest) population, were genotyped using the Affymetrix Human Origins array. This array targets 627,421 SNPs with well-documented ascertainment, although we note that our inference techniques use haplotype information that has been shown to be less affected by ascertainment bias.[18,19] SNPs and individuals were pruned to have genotyping rate greater than 0.95 using PLINK v.1.9.[20] Genotypes for the Indian Zoroastrian and Indian Hindu individuals are available in plink format (see Web Resources). Genotypes for the Iranian Zoroastrians and the Iranian Farsi were made publicly available by Broushaki et al.[12]

The above dataset was merged with modern populations in the Human Origins dataset of Lazaridis et al.,[21] which includes 17 labeled populations from India and Iran (here we use their same labels throughout). We also included other high-coverage ancient samples: an Early Neolithic individual from Iran (WC1),[12] a Mesolithic hunter-gatherer from Luxembourg (Loschbour), Neolithic individuals from Germany (LBK), Anatolia (Bar8[22]), Georgia (KK1[23]), and Hungary (NE1[24]), a 4,500-year-old genome from Ethiopia (Mota[25]), and a 45,000-year-old genome from western Siberia Ust-Ishim.[26] In total, the merge contained 2,553 individuals and 525,796 overlapping SNPs.

Principal Component Analysis (PCA)

We performed PCA on all the South Asian and West European populations included in the merge using PLINK 1.9

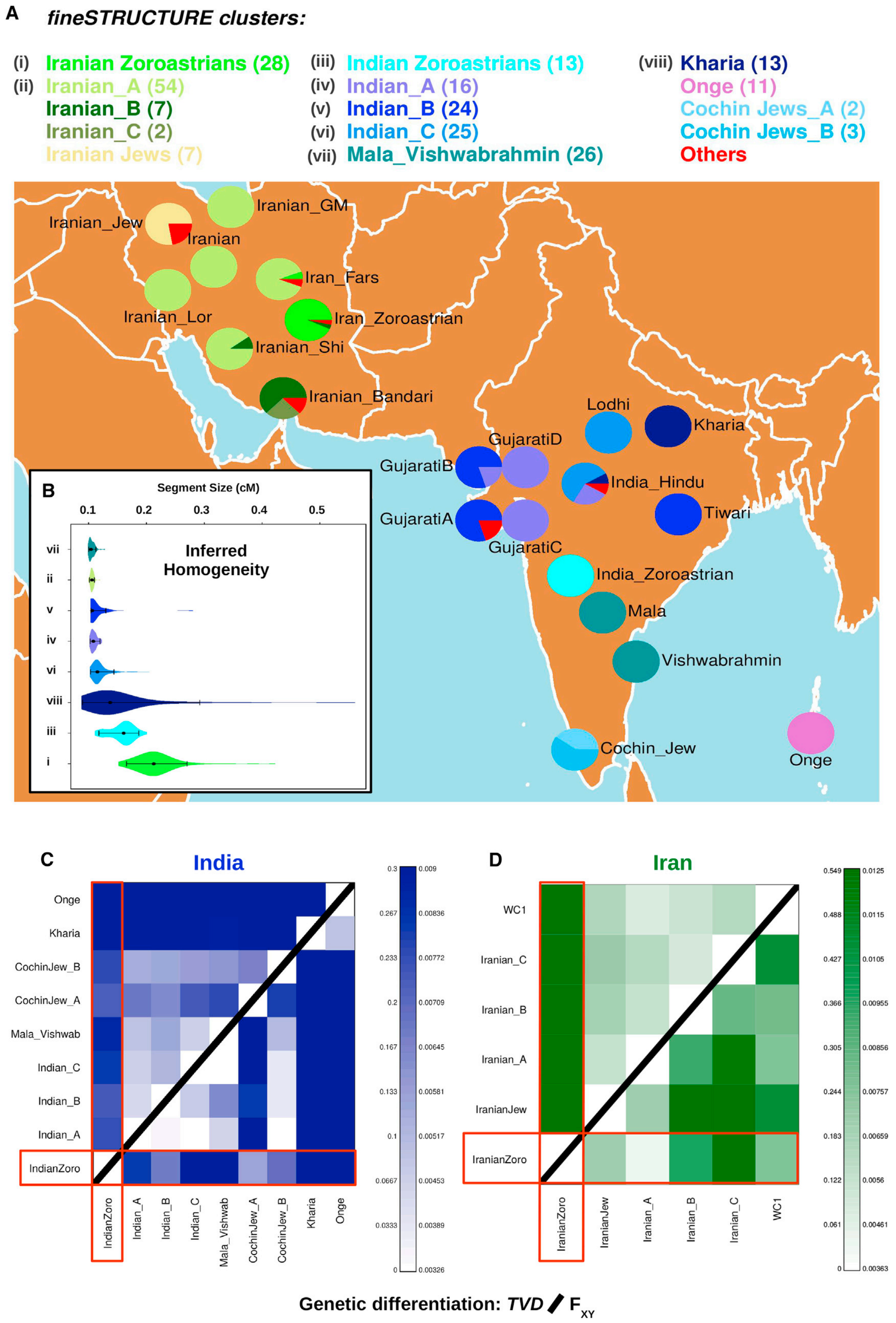

Figure 1. Clustering, Homogeneity, and Genetic Differentiation of the Iranian and Indian Populations
(A) Each color inside the pies represents the proportion of individuals from each population label that is assigned to each fineSTRUCTURE cluster ("Others" include all clusters containing primarily individuals outside Iran and India), with the total number of individuals included in each cluster shown inside brackets in the legend.

(legend continued on next page)

after linkage disequilibrum (LD) pruning using–indep-pairwise 50 5 0.5.

Phasing

We jointly phased the autosomal chromosomes for all individuals in the merge using SHAPEIT[27] with default parameters and the linkage disequilibrium-based genetic map build 37.

Chromosome Painting and fineSTRUCTURE

We classified our 2,545 modern individuals into 230 groups, with the majority of these groups based on population labels.[21] The exceptions to this are the individuals from Iran and India and neighboring populations (see Figure S1) who were re-classified into new, genetics-based groups using results from the clustering algorithm fineSTRUCTURE that groups individuals into genetically homogeneous clusters based entirely on patterns of shared ancestry identified by CHROMOPAINTER.[28] Briefly, CHROMOPAINTER uses a "chromosome painting" approach that compares patterns of haplotype sharing between each recipient chromosome and a set of donor chromosomes.[28] For the CHROMOPAINTER analysis used for our fineSTRUCTURE analysis, which is the first painting protocol described in this paper and referred to throughout as the "fineSTRUCTURE painting," we painted each of the 696 individuals from the populations described above using all other 695 individuals as donors. Here we initially estimated the mutation/emission (Mut, "-M") and switch rate (Ne, "-n") parameters using 10 steps of the Expectation-Maximization (E-M) algorithm, for chromosomes 1, 4, 15, and 22, and for every 10 individuals (following van Dorp et al.[29] and Broushaki et al.[12]), which gave estimated Mut and Ne of 0.00091 and 320.9197, respectively. These values were then fixed before running CHROMOPAINTER across all chromosomes to produce a "painting profile" giving the proportion of genome-wide DNA each individual shares with each other donor individual in this analysis. All chromosomes were then combined to estimate the fineSTRUCTURE normalization parameter "c," which was 0.28. Following Leslie et al.,[30] we then ran fineSTRUCTURE using this c value and performing 2,000,000 iterations of Markov-Chain-Monte-Carlo (MCMC), sampling an inferred clustering every 10,000 iterations. Following the recommended approach described by Lawson et al.,[28] we next used fineSTRUCTURE to find the single MCMC sampled clustering with highest posterior probability and performed 100,000 additional hill-climbing steps to find a nearby state with even higher posterior probability. This hill-climbing approach grouped these 695 individuals into 207 clusters, which we then merged into a tree using fineSTRUCTURE's greedy algorithm that merges pairs of clusters, one step at a time, until only two super-clusters remain.

Based on this tree, exploration of genetic similarity among individuals (see below), and visual inspection of haplotype sharing patterns among our 207 clusters, we classified these clusters into genetically homogeneous groups, choosing a level of the tree where there were 50 final total clusters, among which Iranian Zoroastrians and Parsis each formed their own distinct groups. At this level of the tree, we note that the 10,000-year-old Neolithic Iranian WC1 clustered with other modern Iranians, but nonetheless we re-classified WC1 as its own cluster, so that we ended up with 51 final total clusters we use throughout this paper (see Table S2, Figure S1).

We then painted all 230 modern and 8 ancient samples using all 230 modern groups as donors, following the "leave-one-out" approach, as described by Hellenthal et al.,[31] which is designed to make painting profiles comparable. In particular, if each donor group {1, ..., K} contains {n_1, ..., n_K} individuals, respectively, the set of donors is fixed to contain $n_k - 1$ individuals from each of the K groups. This is to account for the fact that individuals cannot be painted using themselves as a donor, so that individuals within each of these K donor groups can only ever be painted using $n_k - 1$ individuals from their own group label. We refer to this second painting protocol where K = 230 as the "all donors painting" throughout. Note that a primary difference between this painting and the fineSTRUCTURE painting described above is that we now use group labels, based in part on clustering results, which are required for our leave-one-out approach. When using haplotype information for this painting, we initially estimated the mutation/emission and switch rate parameters as described above, giving estimated Mut and Ne of 0.000704 and 223.5674, respectively.

We also performed a slightly different version of this painting where Iranian and Indian populations were excluded as donors, using the leave-one-out approach described above for all 216 remaining groups, a third painting protocol with K = 216 that we refer to throughout as the "non-Indian/Iranian donors painting." We did this to mitigate genetic effects due to recent isolation in our Indian and Iranian groups when inferring the best surrogates to sources of admixture in our groups. Mut and Ne parameters (0.00069 and 225.32, respectively) were re-estimated for this new scenario as described above. To help determine whether admixture from outside groups or independent drift effects due to genetic isolation are driving genetic differences among sampled groups within Iran and India[29,30] (i.e., lower right triangles of Figures 1C, 1D, S2, and S3), we further included all non-Indian groups as donors when painting Indian groups ("non-Indian painting") and all non-Iranian, non-Parsi groups when painting Iranian groups ("non-Iranian/Parsi painting"). This is because in this paper we infer Iranians as important contributors to the DNA of Parsis, making them vital to include when evaluating whether genetic differences among Indian groups are due to admixture.

TVD, F_{XY}, and F_{ST} between Iranian and Indian Groups

We quantified differences in the painting profiles between all Iranian and Indian groups by applying the metric total variation distance (TVD) as described in Leslie et al.[30] using the formula:

$$TVD_{XY} = 0.5\sum_{k=1}^{K}\left(f_k^X - f_k^Y\right).$$

where f_k^X and f_k^Y are the average genome-wide proportion of DNA that individuals from the recipient groups X and Y, respectively, match to donor group $k \in [1, \ldots, K]$ as inferred by CHROMOPAINTER. We also inferred pairwise TVD among

(B) Distribution of CHROMOPAINTER's inferred lengths of haplotype segments (in cM) copied intact from a single donor, when allowing 13 randomly sampled individuals from each group (roman numerals in A) to copy from the other 12 individuals with the same label. (Black dot = median values, bars = 95% empirical quantiles across individuals.)
(C and D) Comparison of pairwise TVD based on the all donors painting (upper triangle) and F_{XY} mitigating recent drift effects (lower triangle) based on the (C) non-Indian donors painting for India and (D) non-Iranian/Parsi donors painting for Iranian groups.

individuals assigned to the same group as defined by our final 51 fineSTRUCTURE-inferred clusters, results of which are discussed in the previous section.

Independent drift effects in groups X and Y since their split can generate genetic differences between them without requiring any outside introgression since this split. To elucidate whether inferred genetic differences are more attributable to ancestry from outside sources, we followed the approach in van Dorp et al.[29] designed to mitigate these drift effects. In particular we calculated:

$$F_{XY} = \frac{TVD_{XY}}{0.5\left(\widetilde{TVD}_X + \widetilde{TVD}_Y\right)}$$

where $\widetilde{TVD}_X$ equals

$$\widetilde{TVD}_X = 0.5\sum_{i=1}^{22}\frac{Li}{L}\left[\sum_{k=1}^{K}|f_{ik}^{X} - f_{k}^{Y}|\right]$$

where L_i is the number of SNPs in chromosome i ϵ [1,...,22], L the total number of SNPs across all the 22 chromosomes, and f_{ik}^{X} is the average proportion of DNA that individuals from X match to donor group k when painting only chromosome i. This approach scales genetic differences between the two groups by differences across chromosomes within each group, exploiting how each chromosome should be subjected to independent drift.[32] For this analysis we used (1) the non-Iranian/Parsi donors painting for Iranian populations and (2) the non-Indian donors painting for Indian populations, which similarly attempt to attenuate drift effects within each Iran and Indian group by matching their DNA to only groups outside of their countries (thus disallowing "self-copying" in Iranian/Indian groups).[29] For comparison purposes, F_{XY} was also calculated for the Iranian and Indian groups using the all donors painting.

The weighted F_{ST} for these groups was also calculated based on independent SNPs using PLINK 1.9, which implements the method introduced by Weir and Cockerham.[33]

Exploring Relative Amounts of Genetic Diversity within Groups

For a comparison of techniques, we used the following three distinct approaches to quantify the relative amounts of genetic diversity within groups.

CHROMOPAINTER Analyses to Infer Relative Amounts of Genetic Diversity within Groups

We performed a fourth analysis using CHROMOPAINTER that is analogous to that in van Dorp et al.[29] to assess the relative genetic diversity within the 8 clusters inferred by fineSTRUCTURE that contained Iranian or Indian individuals and had sample size greater than or equal to 13 (which is the number of Parsi individuals): Indian_A, Indian_B, Indian_C, Parsis, Iranian_A, Iranian_Zoroastrian, Kharia, and Mala_Vishwabrahmin (each defined in Table S2). For each of these 8 clusters, we randomly subsampled 13 individuals and painted each individual using only the other 12 individuals from their respective cluster as donors, using 50 steps of CHROMOPAINTER E-M algorithm inferring the switch and emission rates (i.e., "-i 50 -in -iM"). We refer to this fourth painting protocol throughout as the "within-group-diversity painting." For each individual, we calculated average segment size by dividing the total centimorgan lengths of genome-wide DNA copied from all donors by the total expected number of haplotype segments copied from all donors. This average segment size can be thought of as capturing the relative amount of genome-wide haplotype diversity in each group, with a larger average segment size reflecting relatively less genome-wide diversity.

PLINK IBD Analysis to Infer Relative Amounts of Genetic Diversity within Groups

We also inferred within-group genetic diversity across all pairwise combinations of the same 13 individuals within each of the above genetic clusters using the IBD coefficient PI_HAT implemented in PLINK v.1.9, on a dataset where SNPs were first pruned to remove those in high linkage disequilibrium ($r^2 > 0.2$) in a sliding window of 250 SNPs.

fastIBD Analysis to Infer Relative Amounts of Genetic Diversity within Groups

In order to explore within-group genetic diversity using a third approach, which allows SNPs to be in LD as in our CHROMOPAINTER-based estimates of segment size, we applied fastIBD using the software BEAGLE v.3.3.2.[34] For each cluster, we used fastIBD to infer the pairwise IBD fraction between each pairing of the 13 sub-sampled individuals. For each chromosome of each cluster, fastIBD was run for 10 independent runs and an IBD threshold of 10^{-10} for every pairwise comparison of individuals as recommended by Browning and Browning,[34] though we note results were similar using an IBD threshold of 0.0001.

Inferring Admixture Events via a Mixture Modeling Approach, GLOBETROTTER, f3-Statistics, and TreeMix

As noted previously,[30,31] the inferred CHROMOPAINTER painting profiles are often not an ideal summary of shared ancestry patterns, as for example donor groups with larger sample sizes may be disproportionately represented in these paintings. In order to account for this, we performed additional analyses to "clean" the raw CHROMOPAINTER output. In particular, we applied the Bayesian mixture modeling approach described in Broushaki et al.[12] to infer proportions of ancestry for all recipient groups (which we term "targets") in relation to other included groups that represent potential "surrogates" to sources of ancestry. Here we performed two analyses: (1) including all 229 modern groups excluding the target as potential surrogates and (2) using all 229 modern and 8 ancient groups as potential surrogates (i.e., 237 surrogate groups in total). The aim of this mixture modeling approach is to identify which subset of these 229–237 potential surrogates best reflect the sources of ancestry in the target group. We then use this subset of surrogates in our admixture analysis described below. However, we note that any inferred proportions from this mixture model analysis cannot necessarily be interpreted as reflecting proportions of admixture from distinct source groups. Instead, this mixture modeling step is primarily used to summarize the clearest patterns of shared ancestry between the target and surrogate groups and to restrict the set of surrogates used in our subsequent admixture analysis to help increase power and precision.

We applied GLOBETROTTER,[31] a haplotype-based approach to identify, describe, and date recent admixture events, to test for evidence of admixture separately in each of 24 "target" groups from Iran, India, Pakistan, and Armenia. Roughly speaking, GLOBETROTTER infers admixture in a target group using two (interlocking) steps. The first infers the genetic make-up of the putative admixing source groups, and the second infers the date of admixture. For the first step we used the all donors painting from CHROMOPAINTER for each target group, as this GLOBETROTTER inference step requires each surrogate and target group to be painted using the same (or a very similar) set

of donors.[31] For the second step, we used CHROMOPAINTER to generate ten painting samples per haploid genome for each Iranian, Indian, Pakistani, and Armenian individual, under a different painting where each of these individuals is painted excluding any individuals from their assigned group as donors. We refer to this fifth painting protocol as "GLOBETROTTER painting." We do this GLOBETROTTER painting to follow the suggested protocol in Hellenthal et al.,[31] as including individuals from your own group as donors when painting often substantially masks signals of admixture, particularly when generating the linkage disequilibrium (LD) decay curves critical to dating admixture. This is because individuals (unsurprisingly, but unhelpfully) match large segments of their genome to other individuals from their own group. While we could also use this GLOBETROTTER painting for the first step that infers the genetic make-up of the admixing source groups, for each target group we would then have had to re-paint every surrogate group similarly excluding that target group's individuals as donors. For computational economy we instead used the same all donors painting for each target group, which previous work suggests makes little difference in practice for these sample sizes and which we explore further below.[31] For each target population, we included only the surrogate groups that contributed to our mixture modeling approach described above, separately under the two mixture modeling scenarios using as surrogates (1) modern groups only and (2) modern and ancient groups. We inferred admixture dates using the default LD decay curve range of 1–50 cM and bin size of 0.1 cM when considering the distance between genome segments. An exception to this is cases where the inferred admixture date was >60 generations ago using this default curve range and bin size, in which case we re-estimated dates using a curve range of 1–10 cM and a bin size of 0.05 cM, as using a similar range and bin size has been shown previously to more reliably estimate older dates of admixture.[31] In each analysis we used 5 iterations of GLOBETROTTER's alternating source composition and admixture date inference (num. mixing.iterations: 5) and 100 bootstrap re-samples to infer confidence intervals around the point estimates of the date of admixture. Furthermore, in each case analyses were run twice, once using the option null.ind:0 and once with null.ind:1 to assess the effect of standardizing against a pseudo (null) individual, an approach designed to account for spurious signals of linkage disequilibrium that are not attributable to admixture.[31] Only results for null.ind = 1 are shown, as results for null. ind = 0 were very consistent. For comparison, we also performed an additional GLOBETROTTER analysis using the surrogates inferred under (2) when using CHROMOPAINTER results from the non-Indian/Iranian donors painting, this time using the same CHROMOPAINTER painting for both the first and second steps of GLOBETROTTER described above.

As a very different means of inferring admixture, we also used ADMIXTOOLS[32] to calculate f3 statistics, f3(X; A,B), a commonly used test to detect admixture in a target population X presumed to have received DNA from two ancestral source populations represented by surrogate groups A and B. We inferred admixture separately in the Indian and Iranian Zoroastrians, using all pairwise combinations of the other populations in the dataset, plus the ancient samples, as possible admixture sources A and B.

Additionally, we used TreeMix[35] to infer a bifurcating tree that merges four groups: our Indian and Iranian Zoroastrian groups, and the groups with largest sample size from each of Iran and India. We also included the Yoruba as the outgroup (root) population, assessed results when further including a single migration event among populations in the tree, and accounted for linkage disequilibrium between SNPs in windows of 500 SNPs (-k 500).

Positive Selection Tests

We used the XP-EHH (cross population extended haplotype homozygosity) statistic[36] to detect signatures of recent positive selection by comparing populations with similar demographic histories. In particular we inferred putative regions of positive selection in Zoroastrians of Iran and India, using as reference populations the clusters Iranian_A and Indian_A (for the latter only the individuals labeled as India_Hindu and Gujarati were used due to usage restriction of the other samples for selection tests[21]), respectively. We note that we are applying XP-EHH to populations we infer here to be admixed (see Results). While XP-EHH has been applied to admixed populations before,[37] we caution that this presumably may lead to spurious findings, as proportions of DNA inherited from an introgressing group (which may have more or less linkage disequilibrium than the ancestral group) will vary randomly across genetic regions. This difficulty notwithstanding, normalized XP-EHH scores were calculated using SELSCAN v.1.1.0,[38] with positive XP-EHH values indicating potential selection in the Zoroastrian populations and negative XP-EHH values indicating potential selection in the non-Zoroastrian populations. SNP annotations were obtained using ANNOVAR.[39]

We performed 100 permutation tests to establish the empirical distributions of XP-EHH values across the genome for both the Indian and Iranian populations, both to determine significance thresholds and to assess the reliability of our selection inference. For each permutation, we randomly partitioned our Zoroastrians and non-Zoroastrians into two different groups, and then calculated XP-EHH comparing these two groups. The threshold values at significance level of 0.01% (quantiles 0.0001 and 0.9999) from the empirical distribution combining all 100 permutations were used to determine the significance of the XP-EHH test. These values were −4.46 and 4.46 for the Iran and −4.37 and 4.37 for India.

Y Chromosome Typing and Mitochondrial DNA Sequencing

In order to explore sex-biased admixture and to evaluate claims of patrilineal inheritance among the Parsi priests, all the 526 samples collected for this study were typed for Y chromosome (490 successful samples) and mitochondrial DNA (518 successful sequencing) (see Table S1). Y chromosomes were typed for 6 STRs (DYS19, DYS388, DYS390, DYS391, DYS392, and DYS393) and at 11 biallelic loci (92R7, M9, M13, M17, M20, SRY1465, SRY4064, SRY10831, sY81, Tat, and YAP) as described by Thomas et al.[40] and for the biallelic marker 12f2 as described by Rosser et al.[41] Microsatellite repeat numbers were assigned according to the nomenclature of Kayser et al.[42] For a subset of the samples (Parsi priests), four additional Y chromosome microsatellites (DYS389I, DYS389II, DYS425, and DYS426) were typed as described by Thomas et al.[40] Y chromosome haplogroups (Yhg) were defined by the 12 biallelic markers according to the Y-Chromosome consortium and the most recent nomenclature of ISOGG.

The mitochondrial DNA hyper variable segment 1 (HVS-1) was sequenced as described by Thomas et al.[43] Sequences were obtained for all samples between positions 16,027 and 16,400 according to the numbering scheme of Anderson et al.[44]

mtDNA haplotypes were assigned to haplogroups (iMhg) using HaploGrep.[45]

Unbiased genetic diversity, h, and its standard error were calculated using the formula given by Nei[46] and significant differences in calculated values were found using a standard two-tailed z test. Populations were compared using F_{ST} based on haplotype or haplogroup frequencies, estimated from analysis of molecular variance (AMOVA) $Ø_{ST}$ values,[47,48] and using the exact test for population differentiation.[49] Assessment of the significance of pairwise F_{ST} values was based on 10,000 permutations of the data and 10,000 Markov steps were used in the exact test. Patterns of genetic differentiation were visualized using principal coordinates (PCO) analysis performed on a similarity matrix calculated as $1 - F_{ST}$, based on Yhg and iMhg frequencies. Values along the main diagonal of the similarity matrix, representing the similarity of each population sample to itself, were calculated from the estimated genetic distance between two copies of the same population sample (for $Ø_{ST}$-based F_{ST}, the resulting self-similarity values simplify to $n/(n-1)$, where n is the sample size).

Y chromosome and mtDNA admixture proportions were estimated using the likelihood-based method LEA,[50] based on Yhg and inferred iMhg frequencies. We ran 5,000,000 Monte Carlo iterations of the coalescent simulation and discarded the first 10,000 iterations as burn-in.

The coalescence time of clusters of Y chromosomes belonging to the same UEP-defined haplogroup was estimated by finding the average square difference (ASD) between the inferred ancestral haplotype (in this case the modal haplotype) and all observed chromosomes.[51,52] The 95% confidence interval for this estimate was calculated as described in Thomas et al.[53] using 50,000 iterations. The microsatellite mutation rate was set to 15/7,856, based on data from three published studies.[54–56] This analysis was restricted to haplogroups containing a high-frequency modal haplotype (>50%) where the ancestral state could be inferred with confidence.

Results

Zoroastrians Are Genetically Differentiated from Non-Zoroastrians, with Different Historical Ancestry in Parsis Relative to Non-Zoroastrian Indians

Most of the Iranian Zoroastrians (see Subjects and Methods and Table S1 for a description of the samples used in this work) are positioned within the autosomal genetic variation of other sampled Iranian samples in a PCA of West Eurasian individuals (Figure S4). Interestingly, 2 of the 29 Iranian Zoroastrians (YZ020 and YZ024) look genetically different from the others and were inferred by fineSTRUCTURE to cluster with other non-Zoroastrian Iranians (Figures S1 and S5). In order to study the common ancestry of the genetically homogeneous majority of our sampled Iranian Zoroastrians, these two individuals were excluded from further analysis. The Parsis (i.e., Indian Zoroastrians) form a more wide-ranging cluster along PC1, falling inside Iranian, Pakistani, and Indian groups (Figure S4).

We clustered some of our sampled individuals, including all Indians and Iranians, into 51 genetically homogeneous groups that exhibited good correlation between genetic similarity and population label (Figures 1A and S1, Table S2; see Subjects and Methods for explanation of clustering approach). One of these final 51 clusters contained all 13 Parsis, forming the "Parsis" (or equivalently "Indian Zoroastrians") group we use throughout the remainder of this study. A separate cluster contained 27 of 29 Iranian Zoroastrians plus a single Farsi individual that was very genetically similar to self-identified Zoroastrians (Figure S5, Table S2), and these 28 individuals form the "Iranian Zoroastrian" group we use throughout the remainder of this study. The remaining 2 Iranian Zoroastrians were excluded as outliers as noted above, exhibiting genetic patterns more similar to the Lebanese, Turkish, or Bandari individuals they clustered with than to Zoroastrians (Figure S5). Though there are further splits of our final Iranian and Indian Zoroastrian clusters if we increase the number of clusters beyond 51 (see Figure S1), we note that individuals within each of our final assigned Zoroastrian clusters are very genetically similar to one another. For example, our haplotype-based genetic distance measure TVD[30] (see Subjects and Methods) ranged from 0.0289 to 0.3284 among pairwise comparisons of our 28 Iranian Zoroastrians and from 0.04 to 0.1772 among our 13 Parsis, similar to that within Iranian Jews (0.046–0.308), Kalash (0.02–0.233), Brahui (0.04–0.213), Makrani (0.05–0.2773), and Tiwari (0.0397–0.1605) and hence justifying their merging into a single group to increase power in subsequent analyses (similar to the approach taken by Leslie et al.[30]).

The composition of the remaining genetically homogeneous clusters can be found in Figure 1A and Table S2.

Among our sampled individuals from Armenia, India, Iran, and Pakistan, we measured genetic distance between pairs of groups using two different techniques: (1) the commonly used, allele frequency-based measure F_{ST}[33] and (2) the haplotype-based measure TVD[30] (Table S3, Figures S2 and S3). Genetic distance among groups is not large overall, with e.g., typically $F_{ST} < 0.04$. However, similar to Jewish groups from these regions, the Onge from the isolated Andaman Islands, and the Indian Kharia, an indigenous tribal ethnic group that has been isolated from other groups,[57] Zoroastrians were relatively strongly genetically differentiated from non-Zoroastrians under each of these measures, agreeing with previous work.[10,11,58] For example, the genetic distance between Iranian Zoroastrians and non-Jewish, non-Zoroastrians from Iran ranged from 0.015 to 0.029 for F_{ST} and from 0.544 to 0.551 for TVD, with each distance measure larger than the maximum such measure between any two non-Zoroastrian, non-Jewish Iranian groups (0.011 and 0.164, respectively) (Table S3). Similarly, excluding the Onge and Kharia, the genetic distances between Parsis and non-Zoroastrians from India ranged from 0.014 to 0.028 for F_{ST} and from 0.221 to 0.278 for TVD, with each measure larger than the maximum distance between any two other non-Zoroastrian Indian groups (0.002–0.008 and 0.058–0.122, respectively). Therefore, in both Iran and India, these

Table 1. Measuring within Group Homogeneity: Segment Size (CHROMOPAINTER), FIBD, and PI_HAT

Cluster	Segment (95% CI)	FIBD (95% CI)	PI_HAT (95% CI)
Indian_A	0.108 (0.103–0.117)	0.063 (0.051–0.078)	0.302 (0.297–0.309)
Indian_B	0.106 (0.104–0.128)	0.063 (0.052–0.075)	0.301 (0.296–0.308)
Indian_C	0.115 (0.106–0.14)	0.06 (0.05–0.096)	0.304 (0.299–0.312)
Indian Zoroastrians	0.161 (0.12–0.184)	0.113 (0.074–0.145)	0.312 (0.299–0.324)
Iranian_A	0.106 (0.103–0.11)	0.06 (0.047–0.08)	0.301 (0.294–0.306)
Iranian Zoroastrians	0.212 (0.171–0.271)	0.148 (0.098–0.301)	0.326 (0.31–0.372)
Kharia	0.134 (0.091–0.223)	0.075 (0.052–0.318)	0.323 (0.311–0.412)
Mala_Vishwabrahmin	0.104 (0.101–0.112)	0.061 (0.049–0.089)	0.308 (0.301–0.319)

CHROMOPAINTER's inferred median haplotype segment sizes (in cM) copied intact from a single donor, when allowing 13 randomly sampled individuals from each cluster to copy from the other 12 individuals assigned to the same cluster, using 50 steps of expectation-maximization (E-M). IBD values inferred by fastIBD (FIBD) implemented in BEAGLE v.3.3.2 using the same 13 randomly sampled individuals. PI_HAT values inferred by PLINK v.1.9 across the same 13 randomly sampled individuals after sub-sampling SNPs to remove those in high linkage disequilibrium are also reported. Median and empirical quantile values across the 13 individuals are given for each metric for each cluster.

results indicate a high degree of genetic distance between the Zoroastrians in these countries relative to most other sampled individuals from their respective countries.

Relatively recent isolation may enhance the observed genetic differences between Zoroastrians and other groups, e.g., reflected in how individuals in each Zoroastrian group are inferred to share more recent ancestors with individuals of the same population label than with individuals from other populations under our all donors painting (Figure S1). Therefore, to mitigate genetic differences between Zoroastrians and other groups that are attributable to recent isolation, we performed additional analyses that compared haplotype patterns in our Iranian and Indian groups only to those in groups outside of their respective countries (i.e., the non-Iranian/Parsi donors painting for Iranian populations and non-Indian donors painting for Indian populations; see Subjects and Methods). Relative to our all donors painting, these analyses likely reflect the sharing of ancestors that lived further back in time. Critically, two groups in Iran or India that descend from a similar ancestral source(s) should match to similar outside groups at similar proportions under these analyses, even if those two groups have high F_{ST} between them due to a lack of recent intermixing.[29] For these analyses, we formally compared inferred matching patterns between groups using the haplotype-based genetic distance measure, F_{XY};[29] see Subjects and Methods), which uses independent drift effects across chromosomes to further subtract out genetic differentiation due to recent isolation (see Subjects and Methods).

Under this F_{XY} measure, Iranian Zoroastrians showed a much-reduced genetic distance to other Iranian groups (Figure 1D), e.g., with Zoroastrians and the Iranian_A cluster having the lowest F_{XY} value out of all comparisons of Iranian groups (Table S3). In contrast to results using our F_{ST} and TVD measures, genetic dissimilarities measured by F_{XY} among the non-Zoroastrian Iranian groups are higher. However, the F_{XY} scores are not noticeably lower between the Parsis and non-Zoroastrian groups from India, with in general the Parsis showing a similar relatively high amount of genetic differentiation as the Kharia, Onge, and Indian Cochin Jewish groups to all other Indian groups (Table S3), mimicking our results when comparing these groups using F_{ST} and TVD (Figure 1C).

Genetic Homogeneity Is Higher in Zoroastrian Groups, Consistent with Increased Endogamy Relative to Non-Zoroastrians in Iran and India

Compared to non-Zoroastrian groups, we found that each of Iranian Zoroastrians and Parsis shared relatively longer haplotype segments with members of their own group (Figures 1B and S6, Table 1), reflecting a higher degree of genetic similarity within each Zoroastrian group relative to non-Zoroastrian groups. This is consistent with both Iranian and Indian Zoroastrians being genetically isolated from non-Zoroastrian groups. This is true under two distinct homogeneity estimators that use haplotype information. The first approach, FastIBD,[34] compares the DNA of pairwise combinations of a group's individuals, and here gave median shared haplotype lengths of 0.148 cM and 0.113 cM across pairwise combinations of Iranian Zoroastrians and Parsis, respectively, relative to 0.075 for the third largest value in the Kharia. The second approach, CHROMOPAINTER[28] (under our within-group-diversity painting; see Subjects and Methods), compares the DNA of all of a group's individuals jointly, and here gave median shared haplotype lengths across individuals of 0.212 and 0.161 cM for Iranian Zoroastrians and Parsis, respectively, relative to 0.134 for the third largest value in the Kharia. Conflicting slightly with this, we note that the PI_HAT value from PLINK v.1.9,[20] which is based on an alternative technique that ignores haplotype information when measuring homogeneity, infer the Kharia to have more homogeneity than Parsis, giving median values of 0.323 and 0.312 across pairwise combinations of Kharia and Parsis, respectively. This perhaps results from a

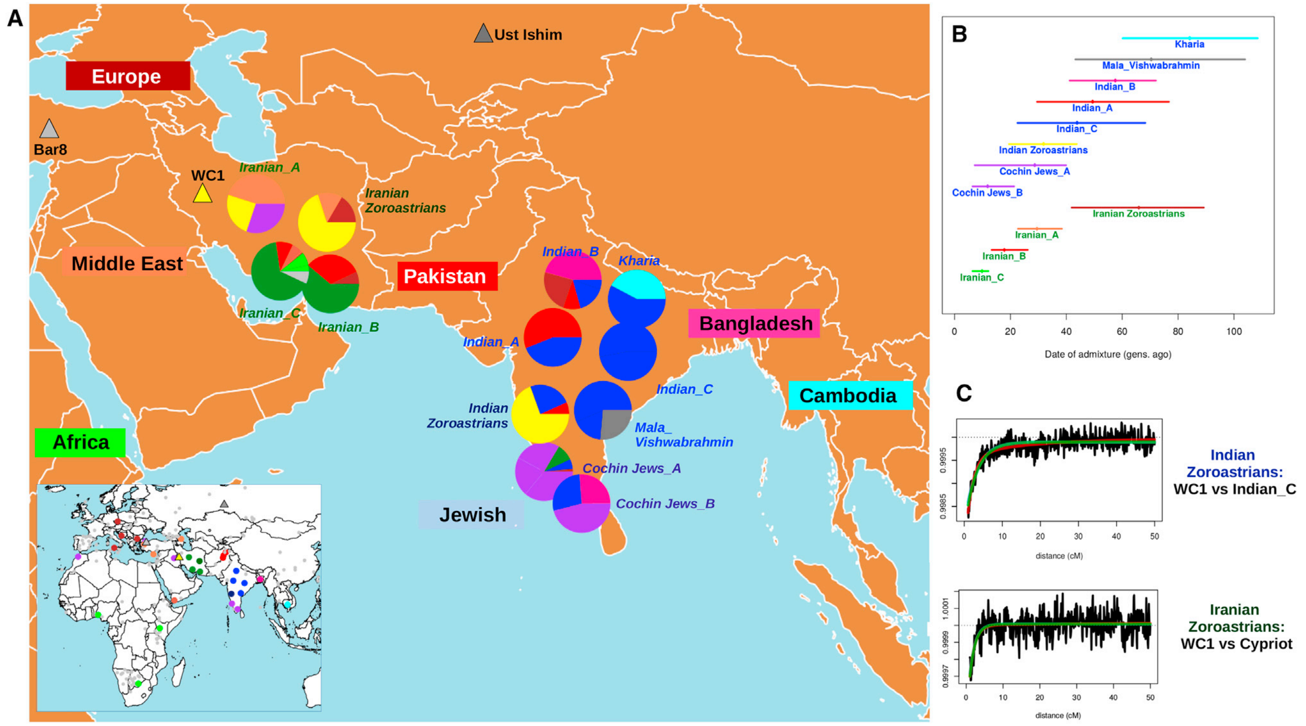

Figure 2. Recent Admixture in India and Iran

(A) Inferred recent admixture in India and Iran under the all donors painting, using admixture surrogates from Europe (brown), Middle East (orange; Yemen in dark orange), Africa (light green), Pakistan (red), Bangladesh (pink), Cambodia (cyan), Iran (dark green), and India (blue) and of Jewish heritage (purple), plus the ancient samples WC1 (yellow), Ust'Ishim (dark gray), and Bar8 (light gray). Inferred proportions of haplotype sharing with each surrogate group are represented in the pie graphs, with all contributing groups highlighted in non-gray in the map in the left bottom corner.

(B) Dates of admixture (dots) and 95% confidence intervals (bars) inferred by GLOBETROTTER, colored according to the surrogate that best reflects the minor contributing admixture source.

(C) GLOBETROTTER coancestry curves, illustrating the weighted probability (black lines) that DNA segments separated by distance x (in cM) match to the two admixture surrogates labeled here, for Parsis (surrogates WC1 versus Indian_C) and Iranian Zoroastrians (surrogates WC1 versus Cypriot), along with the best fitting exponential distributions (green lines) using the inferred date from (B) for each.

decreased resolution when not exploiting linkage disequilibrium information, at least when using ascertained SNPs.[34]

Evidence for Admixture in Zoroastrian Groups with Different Sources and Times using Nuclear Data

We calculated f3 statistics using autosomal DNA from the Iranian Zoroastrians and Parsis as targets and all pairwise combinations of the other modern and ancient groups as sources, reporting all pairwise combinations that gave a negative f3 value with a Z score $> |2|$ for the Parsis in Table S4. In all cases one source of admixture is best represented by a modern-day Indian population. The second source is generally represented by an ancient Neolithic sample from Europe or Anatolia or a modern group close to Iran such as Armenia, Lebanon, or Iraqi_Jews, suggesting an Iranian-like source. In the case of the Iranian Zoroastrians, no admixture events were inferred with any group present in the dataset, consistent with previous reports of f3 statistics sometimes having decreased power to detect admixture in isolated groups with, for example, bottleneck or founder effects.[32]

Additionally, we dated admixture events in both Parsis and Iranian Zoroastrians using the haplotype-based software GLOBETROTTER as described in Subjects and Methods. Analyses using (1) only modern groups and (2) both ancient and modern groups as possible surrogates gave largely corroborating results, e.g., with confidence intervals for dates overlapping when admixture is inferred for the same target group (Figures 2 and S7, Tables S5–S7). However, test (2) was sometimes more sensitive as noted below.

In (1) and (2), we detected admixture in the Parsis dated to 27 (range: 17–38) and 32 (19–44) generations ago, respectively, in each case between one predominantly Indian-like source and one predominantly Iranian-like source. This large contribution from an Iranian-like source (~64%–76%) is not seen in any of our other 7 Indian clusters, though we detect admixture in each of these 7 groups from wide-ranging sources related to modern day individuals from Bangladesh, Cambodia, Europe, Pakistan, or of Jewish heritage (Figures 2 and S7, Tables S5–S7). For Iranian Zoroastrians, we detect admixture only under analysis (2), occurring 66 (42–89) generations ago between a source

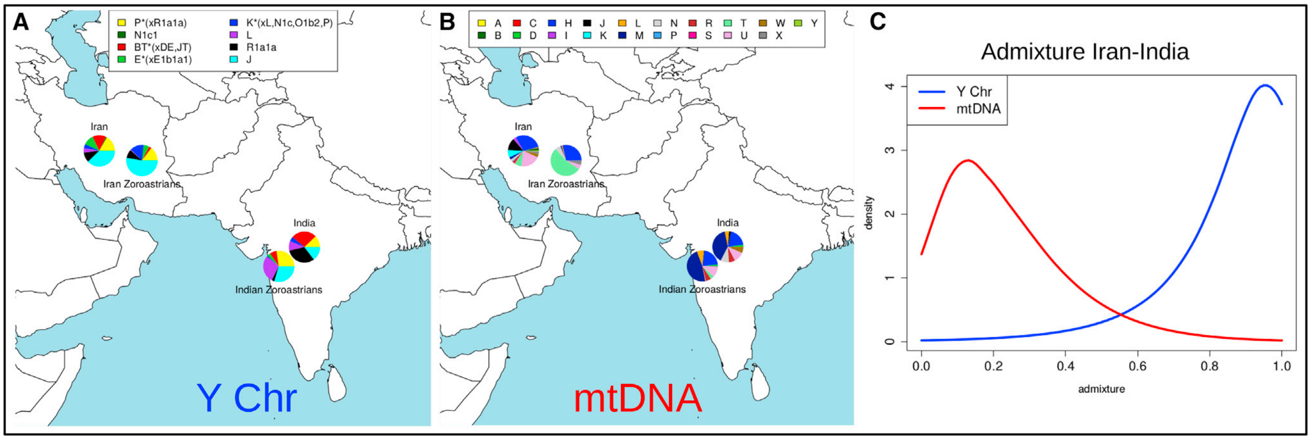

Figure 3. mtDNA and Y Chromosome Variability in Iran and India
(A and B) NRY (A) and mtDNA (B) macrohaplogroup frequencies in India and Iran.
(C) Posterior distribution of Iranian-like admixture proportions in lay Parsis assuming non-Zoroastrian Indian and Iranian lay Zoroastrian surrogate groups, using observed iMhg and Yhg values.

best genetically explained as a mixture of modern-day Croatian and Cypriot samples, and a second source matching to the Neolithic Iranian farmer WC1. We infer admixture in all three other non-Jewish Iranian groups, though consistently more recent (<38 generations ago) with contributions from sources related to modern-day groups from Turkey, Pakistan, and East Africa (Figures 2 and S7, Tables S5–S7). The two Iranian Zoroastrians that had been excluded as outliers exhibited admixture patterns more similar to the Lebanese, Turkish Jews, or Iranian Bandari individuals than to Zoroastrians (Table S8).

We also ran TreeMix in order to infer a bifurcating tree relating five sampled groups: our two Zoroastrian groups, one other Indian group (Indian_C), one other Iranian group (Iranian_A), and Yoruba as an outgroup (Figure S8). This TREEMIX analysis inferred the highest drift value in the Iranian Zoroastrians, in agreement with our analyses described above (Figures 1B and S6, Table S1). Inclusion of a single migration event improved residuals of the fitted tree (Figure S9) but suggested admixture from Parsis into other Indian groups rather than the other way around. This likely reflects the challenge in accurately identifying and describing admixture events in some cases when not directly measuring the decay of linkage disequilibrium that is expected in genuine admixture signals.[31,59]

Evidence for Sex-Biased Admixture in Parsis using Y Chromosome and mtDNA Data

Y chromosome binary polymorphism defined haplogroups (Yhg) and inferred mtDNA haplogroups (iMhg) showed that the Parsi priests sample has the lowest gene diversity values of all population samples studied for both Y and mtDNA (Tables S1, S9, and S10), although we did not have enough data from Iranian Zoroastrian priests to make any analogous observation. The iMhg and Yhg frequency-based pairwise F_{ST} values indicate that through the male line the lay Parsis have a closer relationship to the lay Iranian Zoroastrians, but through the female line they have a closer relationship to the non-Zoroastrians from India (Figure S10). However, no shared Y chromosome STR+biallelic marker or mtDNA control region sequence haplotypes were shared between the Parsi priest and Iranian Zoroastrian priest samples, and all F_{ST} p values and exact tests, whether based on Yhg, Y-haplotype, iMhg, or mtDNA haplotype frequencies, indicated significant differentiation between these two groups.

Using the likelihood-based estimation of admixture (LEA) method of Chikhi et al.[50] as implemented in the LEA software[60] on Yhg and iMhg data, with the non-Zoroastrian Indians and Iranian lay Zoroastrians as surrogates for the two admixing source populations, we infer the most probable Iranian lay Zoroastrian contribution to the lay Parsis Y chromosomes to be 96% (Figure 3C; median = 86%, mean = 82%, 95% CI = 41% to 99%), whereas the most probable Iranian lay Zoroastrian contribution to Parsis mtDNA is 13% (Figure 3C; median = 20%, mean = 24%, 95% CI = 0.05% to 78%). More than 90% of posterior estimates for Y chromosome Iranian lay Zoroastrian contribution to the lay Parsis were higher than the posterior estimates for mtDNA Iranian lay Zoroastrian contribution to the lay Parsis in random samples drawn from each distribution.

Origins of the Parsi Priests

Y/mtDNA data defined 8 Y chromosome haplogroups and 182 total Y chromosome haplotypes when using biallelic and STR loci (Tables S9 and S10) and 240 mtDNA haplotypes that clustered into 14 haplogroups using key HVS-1 mutations. The Parsi priests sample has the lowest gene diversity values of all samples studied for both Y and mtDNA, with the majority of the Parsi priest's Y chromosomes (86%) falling into either Yhg-P*(xR1a1a) or Yhg-L (as defined in Figure S11). The distribution of STR-defined haplotypes within these haplogroups is characterized by

the presence of a high-frequency modal haplotype (>50%), with the remaining haplotypes being only a small number of mutation steps different from the modal haplotype (Figure S11). The exception to this is one "outlier" Yhg-L chromosome that was found to be nine mutation steps different from the nine-microsatellite defined Yhg-L modal haplotype. These data are consistent with the majority of Parsi priests being patrilineal descendants of two male founders in the relatively recent past. Assuming that with the exception of the one Yhg-L outlier, the modals are the ancestral haplotypes[53] to all other chromosomes within each Yhg, we estimate the coalescence dates for these Parsi Yhg-P*(xR1a1a) and Yhg-L chromosomes to be 37 generations (95% CI 19 to 61 generations) and 31 generations (95% CI 18 to 46 generations), respectively. Assuming a generation time of 28 years, this translates to 1,036 years (95% CI 532 to 1,708 years) and 868 years (95% CI 504 to 1,288 years), respectively. Noting that these two coalescence date estimates are not significantly different (only 63% of simulated dates for Yhg-P*(xR1a1a) are older than those for Yhg-L), we re-estimated the coalescence date assuming that both lineages originated at the same time by finding the mean ASD from the respective modal haplotypes for both clusters. This gave a combined within haplogroup coalescence date estimate of 923 years (95% CI 597 to 1,277 years). When uncertainty in the mutation rate estimate is taken into account, the 95% CI widens to 501 to 1,782 years.

Identifying Genetic Regions Showing Evidence of Selection in Zoroastrians Relative to Non-Zoroastrians

We calculated XP-EHH values for Iranian Zoroastrians and Parsis using other Iranians and Indians as reference populations (Figure S12). Tables S11 and S12 provide details for all the SNPs below and above quantiles 0.0001 and 0.9999 of the permutation-based empirical distribution, respectively (see Subjects and Methods), including the genes within those regions, or the flanking genes in the case of intergenic SNPs. However, we note that our permutation procedure shows a very similar distribution of XP-EHH values as those observed in the real data (Figure S13), suggesting that these reported signals should be interpreted with caution.

In the case of the Iranian Zoroastrians, most of the regions with the strongest signals of selection (positive XP-EHH values) are located in intergenic or intronic regions. Among these, some of the most significant SNPs ($p < 0.0001$ based on a permutation procedure; see Subjects and Methods) are located upstream of gene *SLC39A10* (Solute Carrier Family 39 Member 10 [MIM: 608733]) with an important role in humoral immunity[61] or in *CALB2* (Calbindin 2 [MIM: 114051]), which plays a major role in the cerebellar physiology.[62]

With regard to the positive selection tests on Parsis versus India Hindu/Gujarati groups, the most significant SNPs were embedded in *WWOX* (WW domain-containing oxidoreductase [MIM: 605131]), associated with neurological disorders like early epilepsy (MIM: 616211),[63] and in a region in chromosome 20 some core domains from the *WFDC* (acidic protein WAP four-disulfide core domain containing) family and other genes like *SPINT4* (serine peptidase inhibitor, Kunitz type 4), *SNX21* (sorting nexin family member 21), or *TNNC2* (troponin C2, fast skeletal type [MIM: 191039]) (see Table S11 for a complete list). On the other hand, among the SNPs showing signatures of positive selection in the reference Indian population, two highly significant selection signals were identified: *LOC102467224* and *LOC283177*, with unknown functions.

Discussion

Though recent studies have investigated the origins of different Jewish populations from India, like the Cochin Jews or the Bene Israel,[64–66] little is known about the genetic structure of the relatively isolated populations found mainly in India and Iran that practice Zoroastrianism, one of the oldest extant religions in the world. We present genome-scale genetic analyses of Zoroastrians from Iran and India and provide genetic evidence for their historical exodus.[6]

Zoroastrians in both Iran and India are genetically differentiated from other groups in these countries, in Y chromosome, mtDNA, and autosomal patterns of variation (Figures 1, 3, S1–S5, and S10, Tables S2, S3, S9, and S10). However, we found two Iranian Zoroastrian individuals that were genetically distinct from the other Zoroastrians (Figures S4 and S5), suggesting that these individuals were possibly mislabelled or recently converted to Zoroastrianism. The latter would indicate that present-day Zoroastrians in Iran are not as closed a group today as previously reported.[9]

Excluding these two Iranian Zoroastrians, the remaining Zoroastrians in both Iran and India display a high level of genetic homogeneity, greater than any other Iranian and Indian group examined in this study (Figure 1B), despite being sampled from multiple locations. This is likely attributable to founder effects, bottlenecks, and/or some endogamy throughout the last millennium and up to the present day. These factors likely played a major role in the observed differences in autosomal DNA patterns between Iranian Zoroastrians and non-Zoroastrians from Iran, as analyses that attempt to mitigate these genetic isolation effects notably decrease the observed genetic differences between Iranian Zoroastrians and non-Zoroastrian Iranians (Figures 1D and S2, Table S3). In contrast, our analyses to mitigate isolation effects do not drastically affect observed genetic differences between the Indian Zoroastrians (Parsis) and non-Zoroastrian groups from India, suggesting that the different admixture histories of different Indian groups play a major role in shaping observed genetic differences among these Indian groups today (Figures 1C and S3, Table S3).

In particular, we detect an admixture event in the Parsis dated to around 1030 CE (690–1390), between a source genetically similar to modern Indian groups and a second source best represented genetically by a ~9,500-year-old Neolithic farmer from Iran (Figure 2, Table S6). This Iranian source of introgression differs from the sources of admixture inferred in all other sampled Indian groups (Figure 2, Table S6). Our admixture date matches the historical records of a large-scale migration of Zoroastrians to India beginning around 785 CE[5] or 936 CE,[6] providing genetic evidence for this period of migration and suggesting that the migrants mixed with locals soon upon arrival. Our results suggest that these migrations may have resulted in a single "pulse" of admixture occurring around 1030 CE, though our dates are also consistent with multiple episodes of migration from around 690 CE to 1390 CE, which is difficult to disentangle given these sample sizes.[31] However, we see evidence of Iranian-like origins only in Parsis and in no additional sampled non-Zoroastrian groups from India, which strongly indicates our admixture signal is due to the migration of Zoroastrians from Iran rather than being related to historically documented trade between present-day Iran and India[8] that would likely have included mixture among non-Zoroastrian groups.

That our approach inferred the Neolithic Iranian sample WC1 to be a better surrogate for the Iranian admixing source in the Parsis than any modern Iranian groups (including Iranian Zoroastrians) (Figure 2, Table S6) likely results from strong bottleneck effects and/or recent admixture events that have made modern Iranian groups look more genetically differentiated from the Zoroastrian source group that migrated to India ~17–44 generations ago. For example, when performing an alternative analysis that attempts to mitigate genetic isolation effects within each modern Iranian and Indian group by disallowing genetic matching to members from the same assigned cluster (i.e., the non-Indian/Iranian donors painting; see Subjects and Methods), this high aDNA contribution to Parsis is replaced by the modern Iranian Zoroastrians (Table S7, Figure S7). If we instead use the original approach that does not mitigate these isolation effects (i.e., the all donors painting in Figure 2, Table S6) but exclude WC1 as a surrogate, the highest contributing Iranian group to the Parsis is Iranian_A and not the Iranian Zoroastrians (Table S5). The fact that Iranian Zoroastrians are favored as the source of admixture in Parsis only after mitigating isolation effects suggests that at least some of these drift effects in the Iranian Zoroastrians have occurred more recently than the migrations of Parsis to India ~600–1,300 years ago. In contrast, for the Parsis it is difficult to discern the extent to which their relative genetic homogeneity (e.g., Figure 1B) reflects recent isolation since admixture or isolation occurring in their ancestry source from Persia prior to this admixture event.

Patterns of mtDNA and NRY variation also provide clear evidence of contrasting maternal and paternal ancestry in Parsis, consistent with previous studies that suggest that migration of the ancestors of the present-day Parsi population from Iran to India was largely sexually asymmetrical.[67] In particular, it supports Zoroastrianism being brought from Iran to India by a group of males, and/or that gene flow into the Parsi community from the neighboring Indian population was mainly female mediated. Consistent with this, with the genetically estimated and historically attested arrival date of Parsis in India and with the claim of patrilineal descent among Parsi priests, we infer that the majority of Parsi priests are descended from two male founders 923 years (95% CI 597 to 1,277 years) ago. This parallels the Jewish *kohanim* patrilineal priesthood, who claim descent from Moses' brother Aaron and display low Y chromosome diversity, with most Y chromosome STR haplotypes either belonging to or being only a small number of mutation steps away from a modal haplotype.[53]

In Iranian Zoroastrians, we inferred a relatively old admixture event between sources best represented genetically by the Neolithic Iranian WC1 and modern-day Cypriots occurring around 70 CE (range: 570 BCE–750 CE). While we infer admixture in each of the three other non-Jewish Iranian groups (Figures 2 and S7, Tables S5–S7), this admixture date in the Zoroastrians is significantly older, consistent with their long-standing isolation. The date uncertainty and ancient nature of this event prevents interpretation in a clear historical context, but one intriguing possibility is that it reflects mixture among groups joined via the allegiance of the Cypriots with Alexander the Great to help conquer the Persian Empire in 332 BCE. At any rate, interestingly our date range corresponds closely to that spanning the three major Persian empires (Achaemenid, Parthian, Sasanian) for which Zoroastrianism acted as official state religion (559 BCE–651 CE). Ancient DNA from these regions related to these ancient groups and others will greatly enhance our understanding of this older signal.

Notably, when using only modern groups as surrogates and excluding WC1, GLOBETROTTER was not able to detect this older admixture event (Table S5). In this latter analysis, our model considered the Iranian Zoroastrians to be sufficiently genetically matched to a single modern group (Iranian_A) without requiring any other ancestry sources. Presumably this is because Iranian_A has similar genetic patterns to the Iranian Zoroastrians, with GLOBETROTTER inferring similar (but more recent) admixture 20–38 generations ago in Iranian_A between sources best represented by WC1 and modern-day Turkish groups. Our results here suggest that this similarity masks the older DNA contributions to the Zoroastrians. However, the combination of WC1 and other modern groups provides a better match to an ancestral source of the Iranian Zoroastrians than using only Iranian_A, enabling a clear signal of admixture (Figures 2 and S7, Tables S6 and S7). This reveals how adding even small numbers of ancient samples, particularly those less affected by recent admixture, can increase power and insights in population genetic

history inference, even if those ancient samples are substantially older than the time period under study, as is the case here with WC1 living more than 7,000 years earlier.

Our analyses suggest that present-day Iranian Zoroastrians ceased intermixing with other groups shortly after the Arab conquest of Persia during 633–654 CE.[68] In contrast, we infer more recent events in each of our three other non-Jewish Iranian groups (Figures 2 and S7, Tables S5–S7). For example, in Iranian_A we infer an admixture event with an ancestry contribution related to a Turkish-like source group, dated to 1222 CE (range of 95% CI across all 3 GLOBETROTTER analyses: 1026–1362 CE), and hence overlapping with the period of the Seljuq Empire (1037–1194 CE) that spanned parts of present-day Turkey,[69] while in Iranian_B we infer admixture dated to 1418 CE (1194–1558 CE) from a Pakistan-like source that falls into the period of the Timurid Empire (1370–1507 CE) that extended into present-day Pakistan.[70] Finally, we infer our most recent Iranian admixture event in our two Iranian_C individuals, with contributions from an East Africa-like source and an inferred date of 1642 CE (1558–1754 CE) that overlaps with the Safavid Empire (1501–1736 CE[71]) and could be related to the Arab slave trade, as previously observed.[31] More work is required to infer the precise historical nature of these admixture events.

Genetic isolation and endogamous practices can be associated with higher frequencies of disease prevalence. For example, there are reports claiming a high recurrence of diseases such as diabetes[72] among the Iranian Zoroastrians, and Parkinson,[73] colon cancer,[74] or the deficiency of G6PD,[75] an enzyme that triggers the sudden reduction of red blood cells, among the Parsis. Researchers have argued that in addition to these demographic effects, selection can also play a role in the incidence of rare disorders or other phenotypes, as has been previously reported for Ashkenazi Jews.[76,77] Therefore, identifying regions under positive selection in the Zoroastrian populations may be helpful to understand the prevalence of diseases or distinct phenotypic traits in the community. Using XP-EHH[36] comparing Zoroastrians to non-Zoroastrians, we have identified some regions that might have been under selection specifically in the Zoroastrians ($p < 0.0001$ based on a permutation procedure; see Subjects and Methods), as well as in the non-Zoroastrian reference groups (Tables S11 and S12). Some of these regions contain genes that have been associated with different diseases (see Results). However, a permutation study that re-assigned Zoroastrians and non-Zoroastrians randomly to two groups and then tested for selection between these groups gave very similar magnitudes of XP-EHH scores to that seen in our non-permuted data (Figure S13), warranting caution in interpreting these findings and illustrating the challenges in identifying selection signals using genome-wide scans. A larger cohort would be needed to corroborate their significance, coupled with exhaustive epidemiological studies. Nonetheless, they represent a first insight into understanding genetic predisposition and/or resistance to disease in these groups and could form the basis for targeted medical approaches in these isolated groups.

In summary, we explore the genetic landscape and structure of Indian and Iranian Zoroastrians and provide genome-wide genetic evidence that the Parsis descend from an admixture event between ancestral groups consisting predominantly of males with Iranian-related ancestry and females with Indian-related ancestry. We date this event in ancestral Parsis to around 1030 CE, in agreement with historical records. We also provide evidence of a much older admixture event in Iranian Zoroastrians dated to around 74 CE with an unknown historical explanation but overlapping the period where Zoroastrianism acted as state religion in the region. We also demonstrate that Zoroastrians in both countries are genetically homogeneous populations differentiated from other population living locally, likely in part due to religious rules that discourage intermixing with non-Zoroastrians. Further work is required to help understand whether the genetic differences attributable to this isolation correlate with observed differences in disease phenotypes between these communities and other local groups.

Accession Numbers

Genotypes for the Indian Zoroastrian and Indian Hindu individuals are available in plink format at https://doi.org/10.6084/m9.figshare.5223583.v1.

Supplemental Data

Supplemental Data include 13 figures and 12 tables and can be found with this article online at http://dx.doi.org/10.1016/j.ajhg.2017.07.013.

Acknowledgments

S.L. is supported by BBSRC (grant number BB/L009382/1). G.H. is supported by a Sir Henry Dale Fellowship jointly funded by the Wellcome Trust and the Royal Society (grant number 098386/Z/12/Z) and supported by the National Institute for Health Research University College London Hospitals Biomedical Research Centre. M.G.T. was supported by Wellcome Trust Senior Investigator Award (grant number 100719/Z/12/Z). L.v.D. is supported by CoMPLEX via EPSRC (grant number EP/F500351/1). We are grateful to Zoroastrian Studies (Bombay) and in particular to Mr. Khojeste P. Mistree for the facilitation of the project in India and the introduction to priests in all the locations where samples were obtained. We also thank Mrs. Firoza Punthakey Mistree, Ms. Tashan Mistree, and the late Mrs. Shehnaz Neville Munshi for their involvement in the samples collection in India and Iran. We also thank the Children's Hospital of Philadelphia for genotyping the samples on the Affymetrix Human Origins array. G.H. is a founder and director of GENSCI and consultant to LivingDNA. M.E.W. is an employee of Genomics plc, a company providing genomic analysis services to the pharmaceutical and healthcare sectors. His involvement in the conduct of this research was solely in his

capacity as a Reader in Statistical Genetics at King's College London.

Received: April 17, 2017
Accepted: July 24, 2017
Published: August 24, 2017

Web Resources

Genotype data for this paper in plink format, https://doi.org/10.6084/m9.figshare.5223583.v1
ISOGG, https://isogg.org/
OMIM, http://www.omim.org/

References

1. Lamberg-Karlovsky, C.C. (2005). Archaeology and language: the case of the Bronze Age Indo-Iranians. In The Indo-Aryan Controversy. Evidence and Inference in Indian History, E.F. Bryant and L.L. Patton, eds. (London, New York: Taylor & Francis Group), pp. 142–178.
2. Mallory, J.P. (2002). Archaeological models and Asian Indo-Europeans. In Indo-Iranian Languages and Peoples, N. Sims-Williams, ed. (British Academy), pp. 19–42.
3. Skjærvø, P.O. (1996). Zarathustra in the Avesta and in Manicheism. Irano-Manichaica III. In La Persia e l'Asia centrale da Alessandro al X secolo (Accaemia Nazionale dei Lincei), pp. 597–628.
4. Stewart, S. (2013). The Everlasting Flame: Zoroastrianism in History and Imagination (I.B. Tauris).
5. Modi, J.J. (1905). A Few Events in the Early History of the Parsis and Their Dates (Bombay: K.R. Cama Oriental Institute).
6. Hodivala, S.H. (1920). Studies in Parsi History (Bombay).
7. Williams, A. (2009). The Zoroastrian Myth of Migration from Iran and Settlement in the India Diaspora (Brill Academic Publishers).
8. Wink, A. (1990). Al-Hind: The Making of the Islamic World, *Volume 1* (Brill Academic Publishers).
9. Boyce, M. (2001). Zoroastrians: Their Religious Beliefs and Practices (Routledge).
10. Farjadian, S., Sazzini, M., Tofanelli, S., Castrì, L., Taglioli, L., Pettener, D., Ghaderi, A., Romeo, G., and Luiselli, D. (2011). Discordant patterns of mtDNA and ethno-linguistic variation in 14 Iranian ethnic groups. Hum. Hered. *72*, 73–84.
11. Lashgary, Z., Khodadadi, A., Singh, Y., Houshmand, S.M., Mahjoubi, F., Sharma, P., Singh, S., Seyedin, M., Srivastava, A., Ataee, M., et al. (2011). Y chromosome diversity among the Iranian religious groups: a reservoir of genetic variation. Ann. Hum. Biol. *38*, 364–371.
12. Broushaki, F., Thomas, M.G., Link, V., López, S., van Dorp, L., Kirsanow, K., Hofmanová, Z., Diekmann, Y., Cassidy, L.M., Díez-Del-Molino, D., et al. (2016). Early Neolithic genomes from the eastern fertile crescent. Science *353*, 499–503.
13. Stepaniants, M. (2002). The Encounter of Zoroastrianism with Islam (University of Hawai'i Press).
14. al-Maghtheh, M., Ray, V., Mastana, S.S., Garralda, M.D., Bhattacharya, S.S., and Papiha, S.S. (1993). Variation in DNA polymorphisms of the short arm of the human X chromosome: genetic affinity of Parsi from western India. Hum. Hered. *43*, 239–243.
15. Qamar, R., Ayub, Q., Mohyuddin, A., Helgason, A., Mazhar, K., Mansoor, A., Zerjal, T., Tyler-Smith, C., and Mehdi, S.Q. (2002). Y-chromosomal DNA variation in Pakistan. Am. J. Hum. Genet. *70*, 1107–1124.
16. Mohyuddin, A., and Mehdi, S.Q. (2005). HLA analysis of the Parsi (Zoroastrian) population in Pakistan. Tissue Antigens *66*, 691–695.
17. Hinnells, J. (1996). Zoroastrians in Britain (Clarendon Press Oxford).
18. Conrad, D.F., Jakobsson, M., Coop, G., Wen, X., Wall, J.D., Rosenberg, N.A., and Pritchard, J.K. (2006). A worldwide survey of haplotype variation and linkage disequilibrium in the human genome. Nat. Genet. *38*, 1251–1260.
19. Hellenthal, G., Auton, A., and Falush, D. (2008). Inferring human colonization history using a copying model. PLoS Genet. *4*, e1000078.
20. Chang, C.C., Chow, C.C., Tellier, L.C., Vattikuti, S., Purcell, S.M., and Lee, J.J. (2015). Second-generation PLINK: rising to the challenge of larger and richer datasets. Gigascience *4*, 7.
21. Lazaridis, I., Nadel, D., Rollefson, G., Merrett, D.C., Rohland, N., Mallick, S., Fernandes, D., Novak, M., Gamarra, B., Sirak, K., et al. (2016). Genomic insights into the origin of farming in the ancient Near East. Nature *536*, 419–424.
22. Hofmanová, Z., Kreutzer, S., Hellenthal, G., Sell, C., Diekmann, Y., Díez-Del-Molino, D., van Dorp, L., López, S., Kousathanas, A., Link, V., et al. (2016). Early farmers from across Europe directly descended from Neolithic Aegeans. Proc. Natl. Acad. Sci. USA *113*, 6886–6891.
23. Jones, E.R., Gonzalez-Fortes, G., Connell, S., Siska, V., Eriksson, A., Martiniano, R., McLaughlin, R.L., Gallego Llorente, M., Cassidy, L.M., Gamba, C., et al. (2015). Upper Palaeolithic genomes reveal deep roots of modern Eurasians. Nat. Commun. *6*, 8912.
24. Gamba, C., Jones, E.R., Teasdale, M.D., McLaughlin, R.L., Gonzalez-Fortes, G., Mattiangeli, V., Domboróczki, L., Kővári, I., Pap, I., Anders, A., et al. (2014). Genome flux and stasis in a five millennium transect of European prehistory. Nat. Commun. *5*, 5257.
25. Gallego Llorente, M., Jones, E.R., Eriksson, A., Siska, V., Arthur, K.W., Arthur, J.W., Curtis, M.C., Stock, J.T., Coltorti, M., Pieruccini, P., et al. (2015). Ancient Ethiopian genome reveals extensive Eurasian admixture throughout the African continent. Science *350*, 820–822.
26. Fu, Q., Li, H., Moorjani, P., Jay, F., Slepchenko, S.M., Bondarev, A.A., Johnson, P.L., Aximu-Petri, A., Prüfer, K., de Filippo, C., et al. (2014). Genome sequence of a 45,000-year-old modern human from western Siberia. Nature *514*, 445–449.
27. Delaneau, O., Marchini, J., and Zagury, J.F. (2011). A linear complexity phasing method for thousands of genomes. Nat. Methods *9*, 179–181.
28. Lawson, D.J., Hellenthal, G., Myers, S., and Falush, D. (2012). Inference of population structure using dense haplotype data. PLoS Genet. *8*, e1002453.
29. van Dorp, L., Balding, D., Myers, S., Pagani, L., Tyler-Smith, C., Bekele, E., Tarekegn, A., Thomas, M.G., Bradman, N., and Hellenthal, G. (2015). Evidence for a common origin of blacksmiths and cultivators in the Ethiopian Ari within the last 4500 years: lessons for clustering-based inference. PLoS Genet. *11*, e1005397.
30. Leslie, S., Winney, B., Hellenthal, G., Davison, D., Boumertit, A., Day, T., Hutnik, K., Royrvik, E.C., Cunliffe, B., Lawson, D.J., et al.; Wellcome Trust Case Control Consortium 2; and International Multiple Sclerosis Genetics Consortium (2015). The

fine-scale genetic structure of the British population. Nature *519*, 309–314.
31. Hellenthal, G., Busby, G.B.J., Band, G., Wilson, J.F., Capelli, C., Falush, D., and Myers, S. (2014). A genetic atlas of human admixture history. Science *343*, 747–751.
32. Patterson, N., Moorjani, P., Luo, Y., Mallick, S., Rohland, N., Zhan, Y., Genschoreck, T., Webster, T., and Reich, D. (2012). Ancient admixture in human history. Genetics *192*, 1065–1093.
33. Weir, B.S., and Cockerham, C.C. (1984). Estimating F-statistics for the analysis of population structure. Evolution *38*, 1358–1370.
34. Browning, B.L., and Browning, S.R. (2011). A fast, powerful method for detecting identity by descent. Am. J. Hum. Genet. *88*, 173–182.
35. Pickrell, J.K., and Pritchard, J.K. (2012). Inference of population splits and mixtures from genome-wide allele frequency data. PLoS Genet. *8*, e1002967.
36. Sabeti, P.C., Varilly, P., Fry, B., Lohmueller, J., Hostetter, E., Cotsapas, C., Xie, X., Byrne, E.H., McCarroll, S.A., Gaudet, R., et al.; International HapMap Consortium (2007). Genome-wide detection and characterization of positive selection in human populations. Nature *449*, 913–918.
37. Pickrell, J.K., Coop, G., Novembre, J., Kudaravalli, S., Li, J.Z., Absher, D., Srinivasan, B.S., Barsh, G.S., Myers, R.M., Feldman, M.W., and Pritchard, J.K. (2009). Signals of recent positive selection in a worldwide sample of human populations. Genome Res. *19*, 826–837.
38. Szpiech, Z.A., and Hernandez, R.D. (2014). selscan: an efficient multithreaded program to perform EHH-based scans for positive selection. Mol. Biol. Evol. *31*, 2824–2827.
39. Wang, K., Li, M., and Hakonarson, H. (2010). ANNOVAR: functional annotation of genetic variants from high-throughput sequencing data. Nucleic Acids Res. *38*, e164.
40. Thomas, M.G., Bradman, N., and Flinn, H.M. (1999). High throughput analysis of 10 microsatellite and 11 diallelic polymorphisms on the human Y-chromosome. Hum. Genet. *105*, 577–581.
41. Rosser, Z.H., Zerjal, T., Hurles, M.E., Adojaan, M., Alavantic, D., Amorim, A., Amos, W., Armenteros, M., Arroyo, E., Barbujani, G., et al. (2000). Y-chromosomal diversity in Europe is clinal and influenced primarily by geography, rather than by language. Am. J. Hum. Genet. *67*, 1526–1543.
42. Kayser, M., Caglià, A., Corach, D., Fretwell, N., Gehrig, C., Graziosi, G., Heidorn, F., Herrmann, S., Herzog, B., Hidding, M., et al. (1997). Evaluation of Y-chromosomal STRs: a multicenter study. Int. J. Legal Med. *110*, 125–133, 141–149.
43. Thomas, M.G., Weale, M.E., Jones, A.L., Richards, M., Smith, A., Redhead, N., Torroni, A., Scozzari, R., Gratrix, F., Tarekegn, A., et al. (2002). Founding mothers of Jewish communities: geographically separated Jewish groups were independently founded by very few female ancestors. Am. J. Hum. Genet. *70*, 1411–1420.
44. Anderson, S., Bankier, A.T., Barrell, B.G., de Bruijn, M.H., Coulson, A.R., Drouin, J., Eperon, I.C., Nierlich, D.P., Roe, B.A., Sanger, F., et al. (1981). Sequence and organization of the human mitochondrial genome. Nature *290*, 457–465.
45. Kloss-Brandstätter, A., Pacher, D., Schönherr, S., Weissensteiner, H., Binna, R., Specht, G., and Kronenberg, F. (2011). HaploGrep: a fast and reliable algorithm for automatic classification of mitochondrial DNA haplogroups. Hum. Mutat. *32*, 25–32.
46. Nei, M. (1987). Molecular Evolutionary Genetics (Columbia University Press).
47. Reynolds, J., Weir, B.S., and Cockerham, C.C. (1983). Estimation of the coancestry coefficient: basis for a short-term genetic distance. Genetics *105*, 767–779.
48. Michalakis, Y., and Excoffier, L. (1996). A generic estimation of population subdivision using distances between alleles with special reference for microsatellite loci. Genetics *142*, 1061–1064.
49. Raymond, M., and Rousset, F. (1995). An exact test of population differentiation. Evolution *49*, 1280–1283.
50. Chikhi, L., Bruford, M.W., and Beaumont, M.A. (2001). Estimation of admixture proportions: a likelihood-based approach using Markov chain Monte Carlo. Genetics *158*, 1347–1362.
51. Slatkin, M. (1995). A measure of population subdivision based on microsatellite allele frequencies. Genetics *139*, 457–462.
52. Goldstein, D.B., Ruiz Linares, A., Cavalli-Sforza, L.L., and Feldman, M.W. (1995). An evaluation of genetic distances for use with microsatellite loci. Genetics *139*, 463–471.
53. Thomas, M.G., Skorecki, K., Ben-Ami, H., Parfitt, T., Bradman, N., and Goldstein, D.B. (1998). Origins of Old Testament priests. Nature *394*, 138–140.
54. Bianchi, N.O., Catanesi, C.I., Bailliet, G., Martinez-Marignac, V.L., Bravi, C.M., Vidal-Rioja, L.B., Herrera, R.J., and López-Camelo, J.S. (1998). Characterization of ancestral and derived Y-chromosome haplotypes of New World native populations. Am. J. Hum. Genet. *63*, 1862–1871.
55. Heyer, E., Puymirat, J., Dieltjes, P., Bakker, E., and de Knijff, P. (1997). Estimating Y chromosome specific microsatellite mutation frequencies using deep rooting pedigrees. Hum. Mol. Genet. *6*, 799–803.
56. Kayser, M., Roewer, L., Hedman, M., Henke, L., Henke, J., Brauer, S., Krüger, C., Krawczak, M., Nagy, M., Dobosz, T., et al. (2000). Characteristics and frequency of germline mutations at microsatellite loci from the human Y chromosome, as revealed by direct observation in father/son pairs. Am. J. Hum. Genet. *66*, 1580–1588.
57. Vidyarthi, L.P., and Upadhyay, V.S. (1980). The Kharia: Then and Now: A Comparative Study of Hill, Dhelki, and Dudh Kharia of the Central-eastern region of India (Concept).
58. Reich, D., Thangaraj, K., Patterson, N., Price, A.L., and Singh, L. (2009). Reconstructing Indian population history. Nature *461*, 489–494.
59. Loh, P.R., Lipson, M., Patterson, N., Moorjani, P., Pickrell, J.K., Reich, D., and Berger, B. (2013). Inferring admixture histories of human populations using linkage disequilibrium. Genetics *193*, 1233–1254.
60. Langella, O., Chikhi, L., and Beaumont, M.A. (2001). LEA (Likelihood-based estimation of admixture): a program to simultaneously estimate admixture and the time since admixture. Mol. Ecol. Resour. *1*, 357–358.
61. Hojyo, S., Miyai, T., Fujishiro, H., Kawamura, M., Yasuda, T., Hijikata, A., Bin, B.H., Irié, T., Tanaka, J., Atsumi, T., et al. (2014). Zinc transporter SLC39A10/ZIP10 controls humoral immunity by modulating B-cell receptor signal strength. Proc. Natl. Acad. Sci. USA *111*, 11786–11791.
62. Schiffmann, S.N., Cheron, G., Lohof, A., d'Alcantara, P., Meyer, M., Parmentier, M., and Schurmans, S. (1999). Impaired motor coordination and Purkinje cell excitability in mice lacking calretinin. Proc. Natl. Acad. Sci. USA *96*, 5257–5262.

63. Abdel-Salam, G., Thoenes, M., Afifi, H.H., Körber, F., Swan, D., and Bolz, H.J. (2014). The supposed tumor suppressor gene WWOX is mutated in an early lethal microcephaly syndrome with epilepsy, growth retardation and retinal degeneration. Orphanet J. Rare Dis. *9*, 12.
64. Waldman, Y.Y., Biddanda, A., Dubrovsky, M., Campbell, C.L., Oddoux, C., Friedman, E., Atzmon, G., Halperin, E., Ostrer, H., and Keinan, A. (2016). The genetic history of Cochin Jews from India. Hum. Genet. *135*, 1127–1143.
65. Waldman, Y.Y., Biddanda, A., Davidson, N.R., Billing-Ross, P., Dubrovsky, M., Campbell, C.L., Oddoux, C., Friedman, E., Atzmon, G., Halperin, E., et al. (2016). The genetics of Bene Israel from India reveals both substantial Jewish and Indian ancestry. PLoS ONE *11*, e0152056.
66. Chaubey, G., Singh, M., Rai, N., Kariappa, M., Singh, K., Singh, A., Pratap Singh, D., Tamang, R., Selvi Rani, D., Reddy, A.G., et al. (2016). Genetic affinities of the Jewish populations of India. Sci. Rep. *6*, 19166.
67. McElreavey, K., and Quintana-Murci, L. (2005). A population genetics perspective of the Indus Valley through uniparentally-inherited markers. Ann. Hum. Biol. *32*, 154–162.
68. Akram, A.I. (2009). The Muslim Conquest of Persia (Maktabah Publications).
69. Jackson, P. (2002). Review: the history of the Seljuq Turkmens. J. Islamic Stud. *13*, 75–76.
70. Subtelny, M. (2007). Timurids in Transition: Turko-Persian Politics an Acculturation in Medieval Iran (BRILL).
71. Newman, A.J. (2006). Safavid Iran: Rebirth of a Persian Empire (I. B. Tauris).
72. Khalilzadeh, S., Afkhami-Ardekani, M., and Afrand, M. (2015). High prevalence of type 2 diabetes and pre-diabetes in adult Zoroastrians in Yazd, Iran: a cross-sectional study. Electron. Physician *7*, 998–1004.
73. Bharucha, N.E., Bharucha, E.P., Bharucha, A.E., Bhise, A.V., and Schoenberg, B.S. (1988). Prevalence of Parkinson's disease in the Parsi community of Bombay, India. Arch. Neurol. *45*, 1321–1323.
74. Jussawalla, D.J., and Gangadharan, P. (1977). Cancer of the colon: 32 years of experience in Bombay, India. J. Surg. Oncol. *9*, 607–622.
75. Baxi, A.J., Balakrishnan, V., Undevia, J.V., and Sanghvi, L.D. (1963). Glucose-6-phosphate dehydrogenase deficiency in the Parsee community, Bombay. Indian J. Med. Sci. *17*, 493–500.
76. Goodman, R.M., and Motulsky, A.G. (1979). Genetic Diseases Among Ashkenazi Jews (New York: Raven Press).
77. Bray, S.M., Mulle, J.G., Dodd, A.F., Pulver, A.E., Wooding, S., and Warren, S.T. (2010). Signatures of founder effects, admixture, and selection in the Ashkenazi Jewish population. Proc. Natl. Acad. Sci. USA *107*, 16222–16227.

The American Journal of Human Genetics, Volume *101*

Supplemental Data

The Genetic Legacy of Zoroastrianism in Iran and India: Insights into Population Structure, Gene Flow, and Selection

Saioa López, Mark G. Thomas, Lucy van Dorp, Naser Ansari-Pour, Sarah Stewart, Abigail L. Jones, Erik Jelinek, Lounès Chikhi, Tudor Parfitt, Neil Bradman, Michael E. Weale, and Garrett Hellenthal

Supplemental Figures

Figure S1. FineSTRUCTURE heatmap and tree. Inferred proportion of genome-wide DNA that each of the clusters inferred by fineSTRUCTURE (columns) copy from each of these clusters (rows), displayed as a heatmap. The tree at top shows hierarchical merging of the clusters inferred by fineSTRUCTURE. The green lines show how the 207 clusters at the finest level of the tree were classified into 50 groups (a separate cluster we generated that contains only the Neolithic Iranian farmer sample "WC1" is not shown in this heatmap). The populations analysed here include the novel samples generated in this work (Indian_Zoroastrian, Indian Hindu, Iranian_Zoroastrian, Iranian_Fars) plus the populations originally labelled in the Lazaridis dataset as: Onge, Mala, Tiwari, Kharia, Lodhi, Vishwabrahmin, GujaratiD_GIH, GujaratiB_GIH, GujaratiA_GIH, GujaratiC_GIH, Cochin_Jew, Iranian, Iranian_Bandari, Iranian_GM, Iranian_Shi, Iranian_Lor, Iranian_Jew, Brahui, Balochi, Hazara, Makrani, Sindhi, Pathan, Kalash, Burusho, Punjabi_Lahore_PJL, Druze, BedouinB, BedouinA, Palestinian, Syrian, Lebanese, Jordanian, Yemen, Georgian_Megrels, Abkhasian, Armenian, Lebanese_Christian, Lebanese_Muslim, Assyrian, Yemenite_Jew, Turkish_Jew, Turkish_Kayseri, Turkish_Balikesir, Turkish, Turkish_Istanbul, Turkish_Adana, Turkish_Trabzon, Turkish_Aydin, Iraqi_Jew, Georgian_Jew, AltaiNea, DenisovaPinky, and additional ancient DNA (aDNA) samples we label here as UstIshim, Mota, KK1, LBK, Loschbour, NE1, Bar8, WC1).

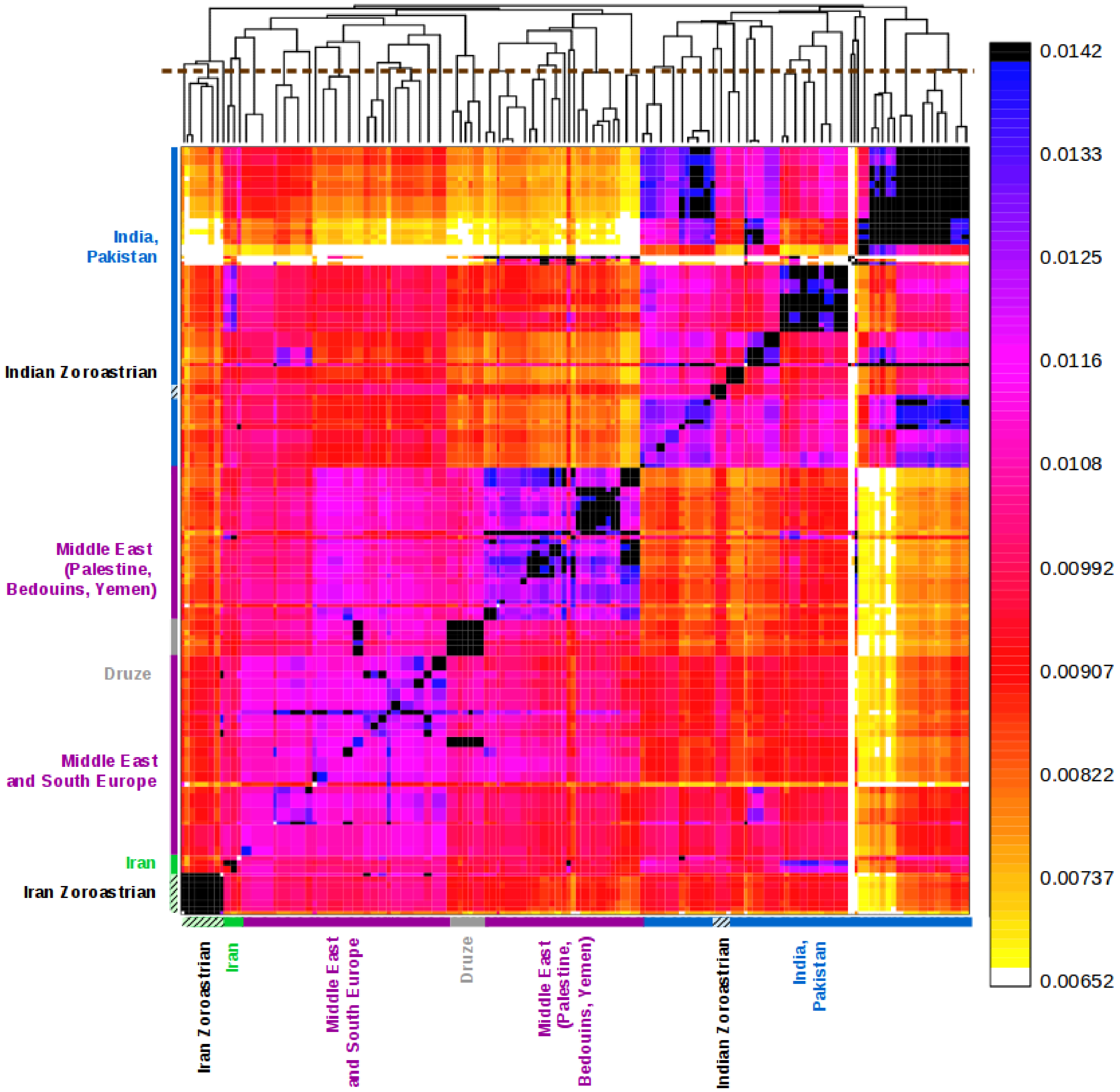

India,
Pakistan
Indian Zoroastrian
Middle East
(Palestine,
Bedouins, Yemen)
Druze
Middle East
and South Europe
Iran
Iran Zoroastrian
Iran Zoroastrian
Iran
Middle East
and South Europe
Druze
Middle East
(Palestine,
Bedouins, Yemen)
Indian Zoroastrian
India,
Pakistan
0.0142
0.0133
0.0125
0.0116
0.0108
0.00992
0.00907
0.00822
0.00737
0.00652

Figure S2: Comparison of the pairwise F_{XY} based on the "all donors painting" (upper left triangle) and F_{XY} based on the "non Iranian/Parsi donors painting" (lower right triangle) for the Iranian groups. Note that Iranian Zoroastrians are not very strongly differentiated from other Iranian groups in the bottom triangle, indicating isolation effects rather than admixture from outside groups are likely driving differences in the top left triangle.

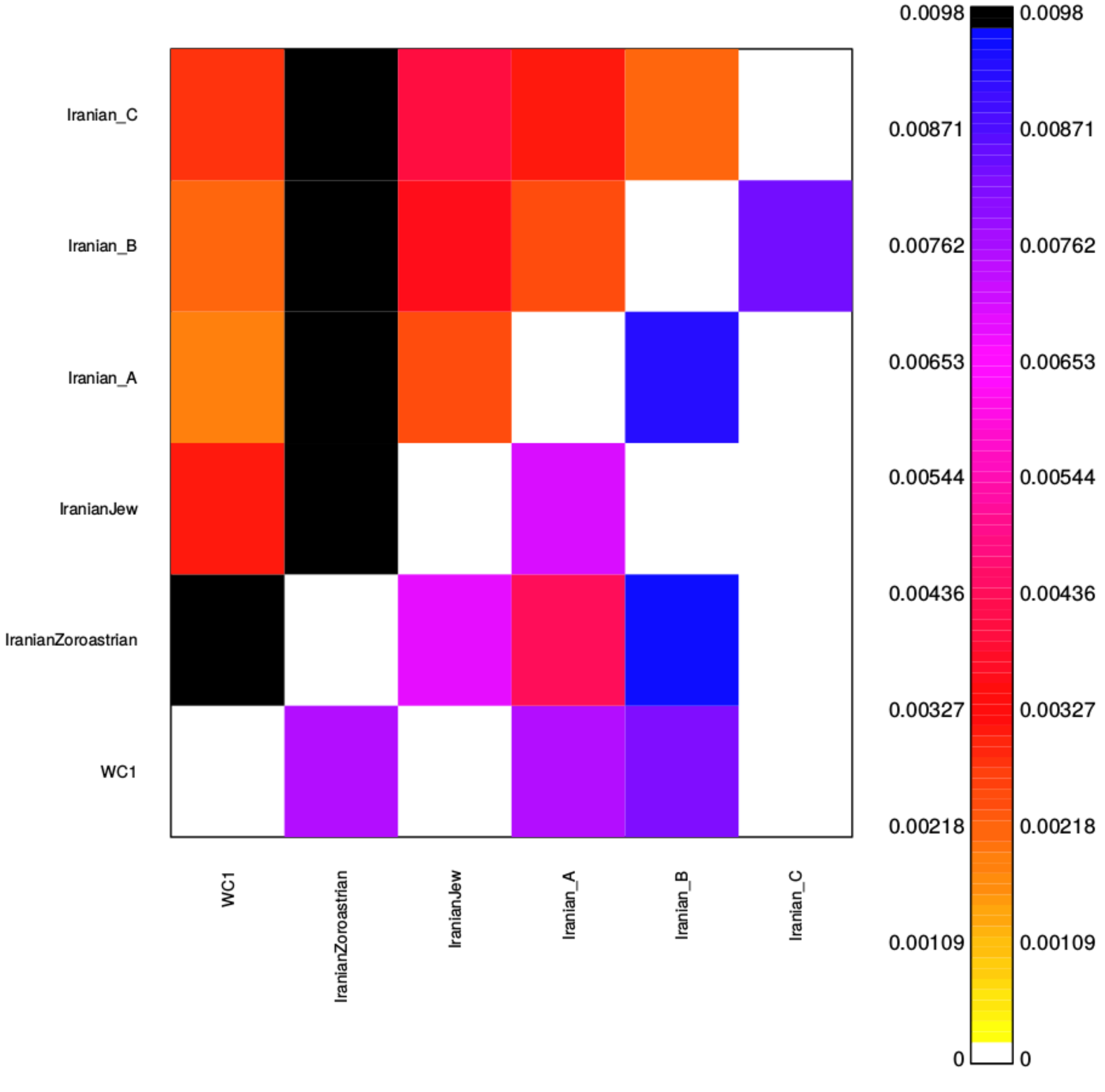

Figure S3: Comparison of the pairwise F_{XY} based on the "all donors painting" (upper left triangle) and F_{XY} based on the "non Indian donors painting" (lower right triangle) for the Indian groups.

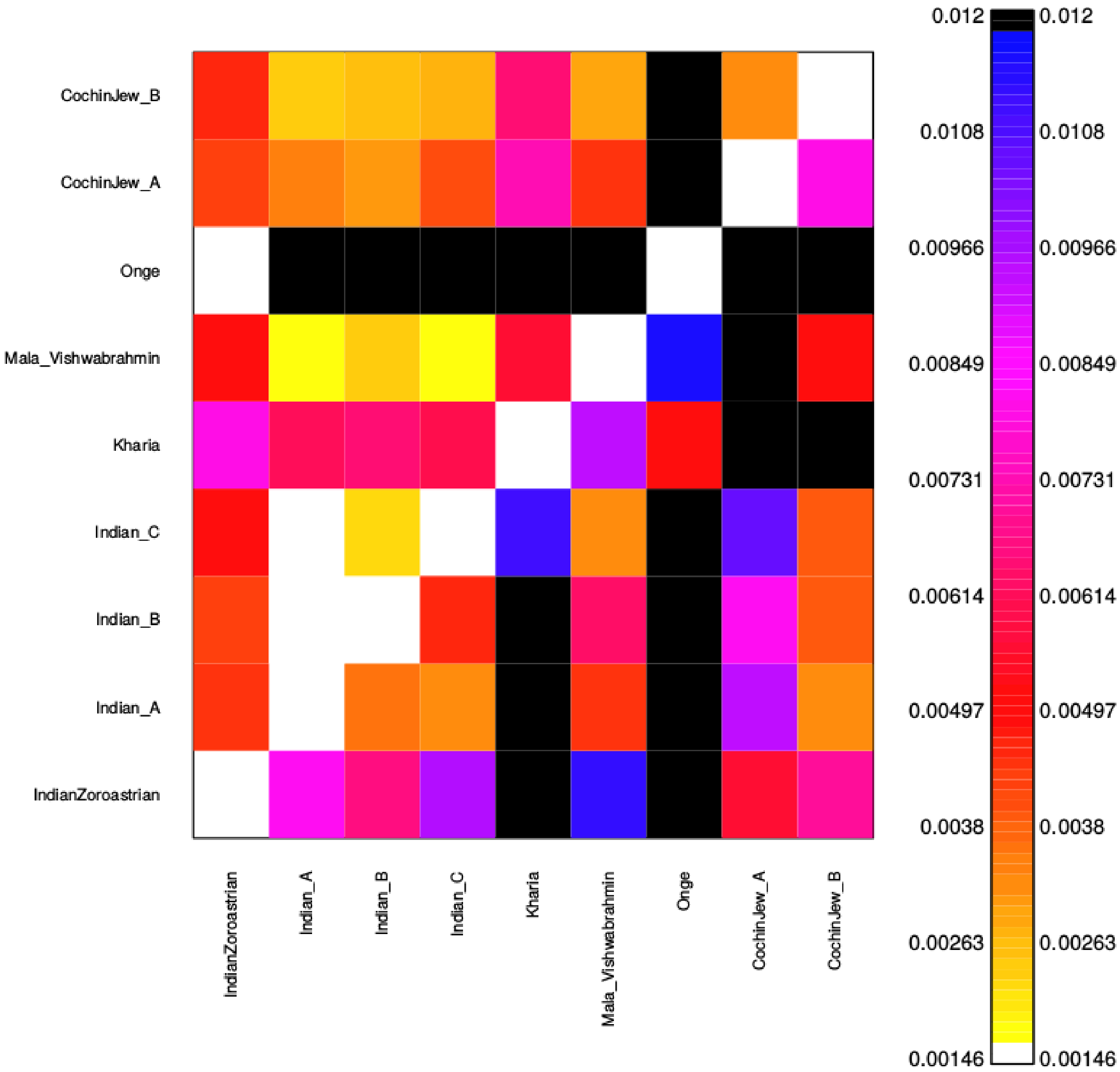

Figure S4. Principal Component Analysis (PCA) of the South-West Eurasian populations included in the merge. Iranian and Indian group labels are highlighted in green and blue, respectively.

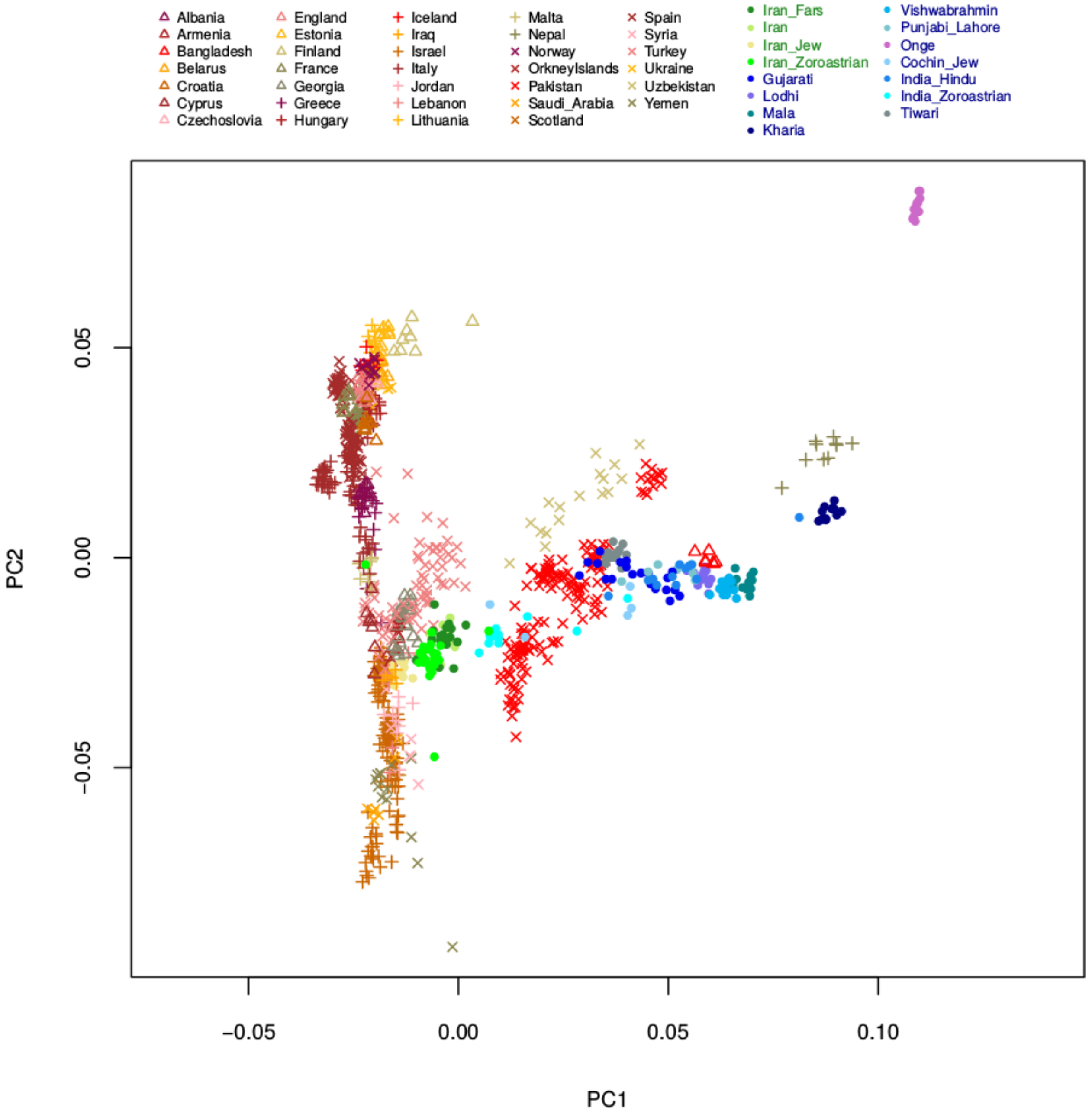

Figure S5. Proportion of haplotypes each Iranian, Indian, Pakistani and Armenian individual (columns) shares with modern groups (rows) from different geographic regions (y-axis labels). The right heatmap shows these proportions for (left to right): the ancient Iranian farmer WC1, a labeled Iranian Zoroastrian (YZ020) that clusters with non-Iranian Zoroastrians, averaged across all sampled Bandari, averaged across all sampled Iranian Fars excluding the outliers, averaged across all sampled Iranian Zoroastrians excluding the outliers, a labeled Iranian Fars individual (IREJ-T053) that clusters with the Iranian Zoroastrians, averaged across all sampled Iranian Jews and a labeled Iranian Zoroastrian (YZ024) that clusters with other non-Iranian individuals (most strongly with Sephardi Jews sampled from Turkey). Green rectangles enclose samples that cluster together using fineSTRUCTURE.

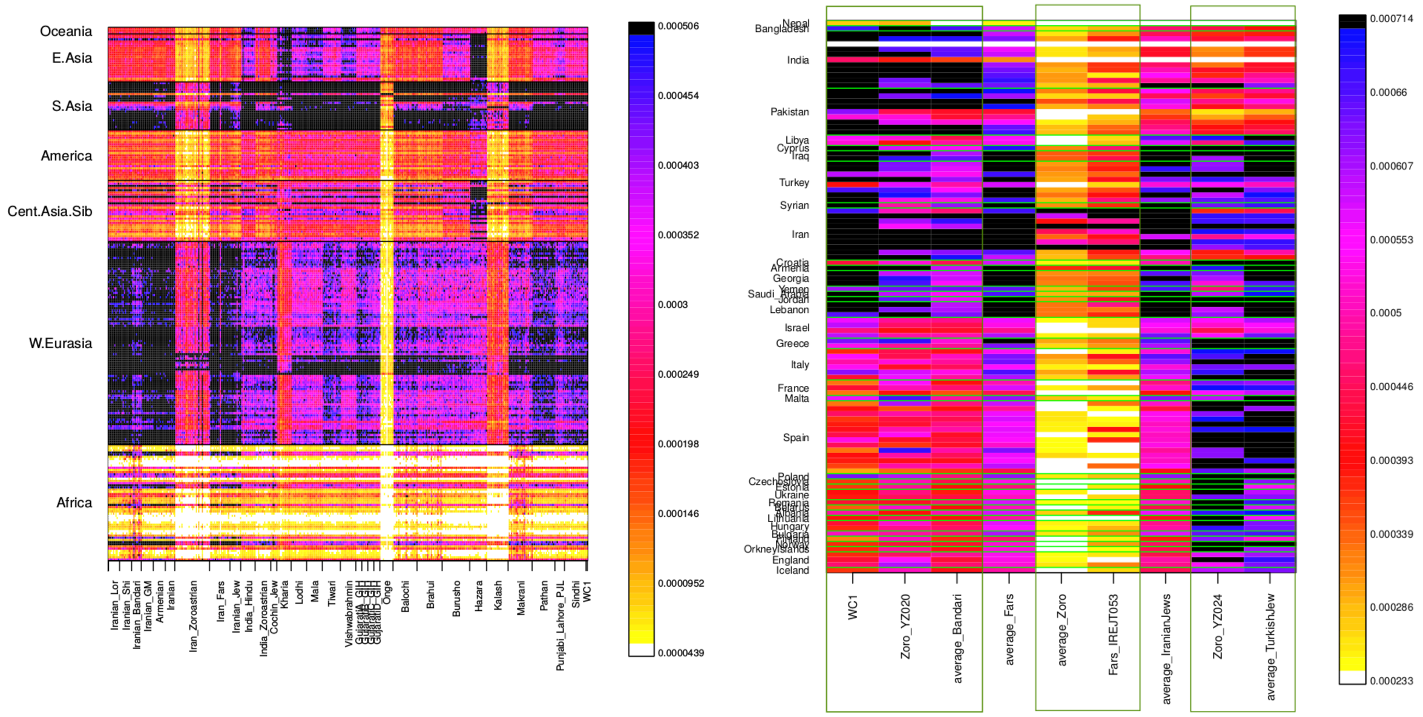

Figure S6: Pairwise comparison of the three methods used to assess genetic homogeneity: CHROMOPAINTER's inferred average haplotype segment sizes (in cM) versus PI_HAT values inferred by PLINK v1.9 and fastIBD inferred IBD coefficient (FIBD). Median and 95% empirical quantile values across all individuals (segment size) or across all pairwise comparisons of individuals (PI_HAT and FIBD estimates) are shown.

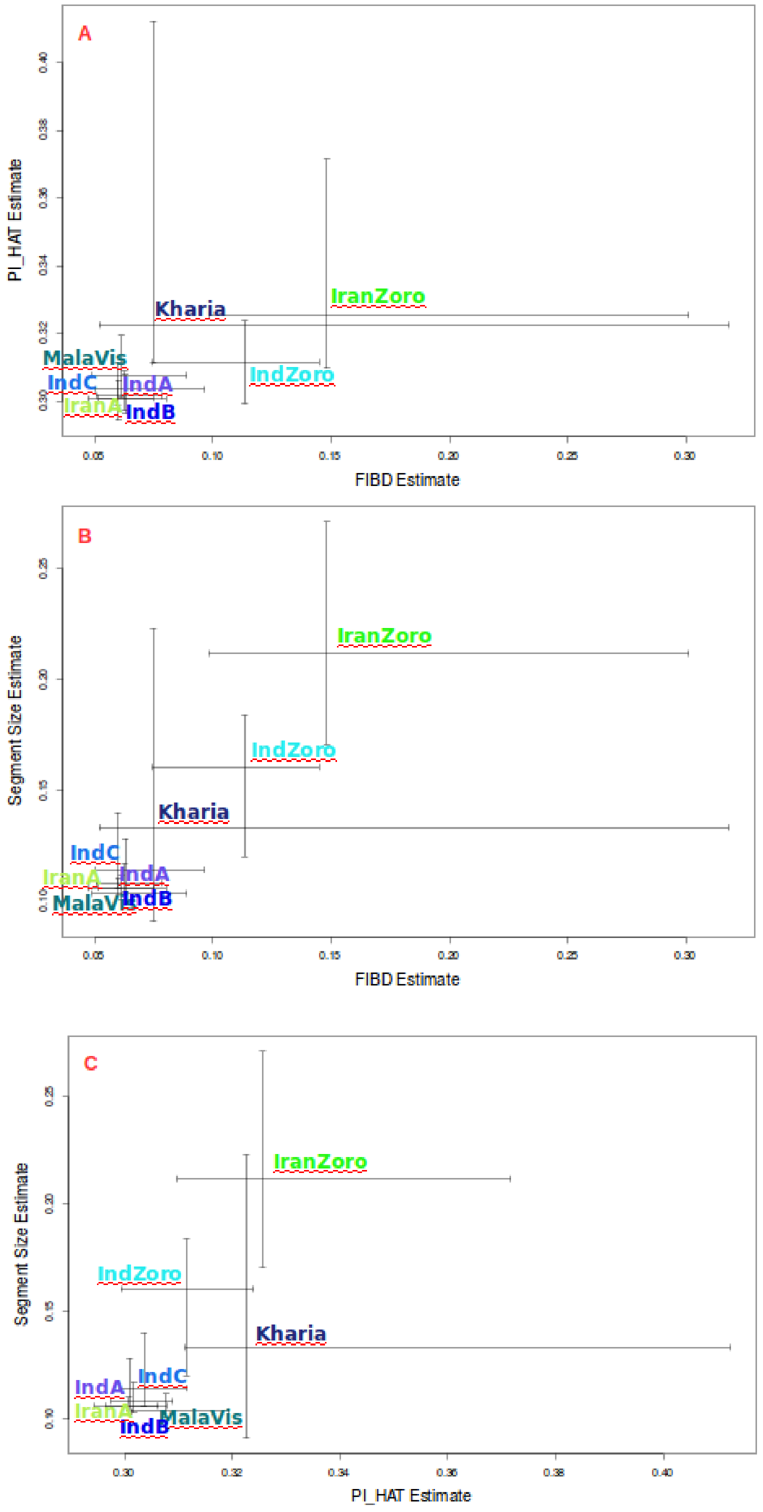

Figure S7: Inferred r**ecent admixture in India and Iran when excluding Iranian and Indian populations as donors in the painting profiles (i.e. "non-Indian/Iranian donors painting"),** using surrogates from Europe (brown), Middle East (orange; Yemen in dark orange), Africa (light green), Pakistan (red), Bangladesh (pink), Cambodia (cyan), Iran (dark green) and India (blue) and of Jewish heritage (purple), plus the ancient samples WC1 (yellow), Ust'Ishim (dark grey) and Bar8 (grey). Proportions of ancestry inferred from each surrogate group are represented in the pie graphs, with all contributing groups highlighted in non-grey in the map in the left bottom corner. Dates of admixture and 95% confidence intervals inferred by GLOBETROTTER are shown on the top right, colored according to the surrogate that best reflects the minor contributing admixture source. GLOBETROTTER coancestry curves, illustrating the weighted probability that DNA segments separated by distance x (in cM) match to the two admixture surrogates given in the title, for Parsis (Iranian Zoroastrians vs Indian_C) and Iranian Zoroastrians (Indian Zoroastrians vs Greek) in the bottom right.

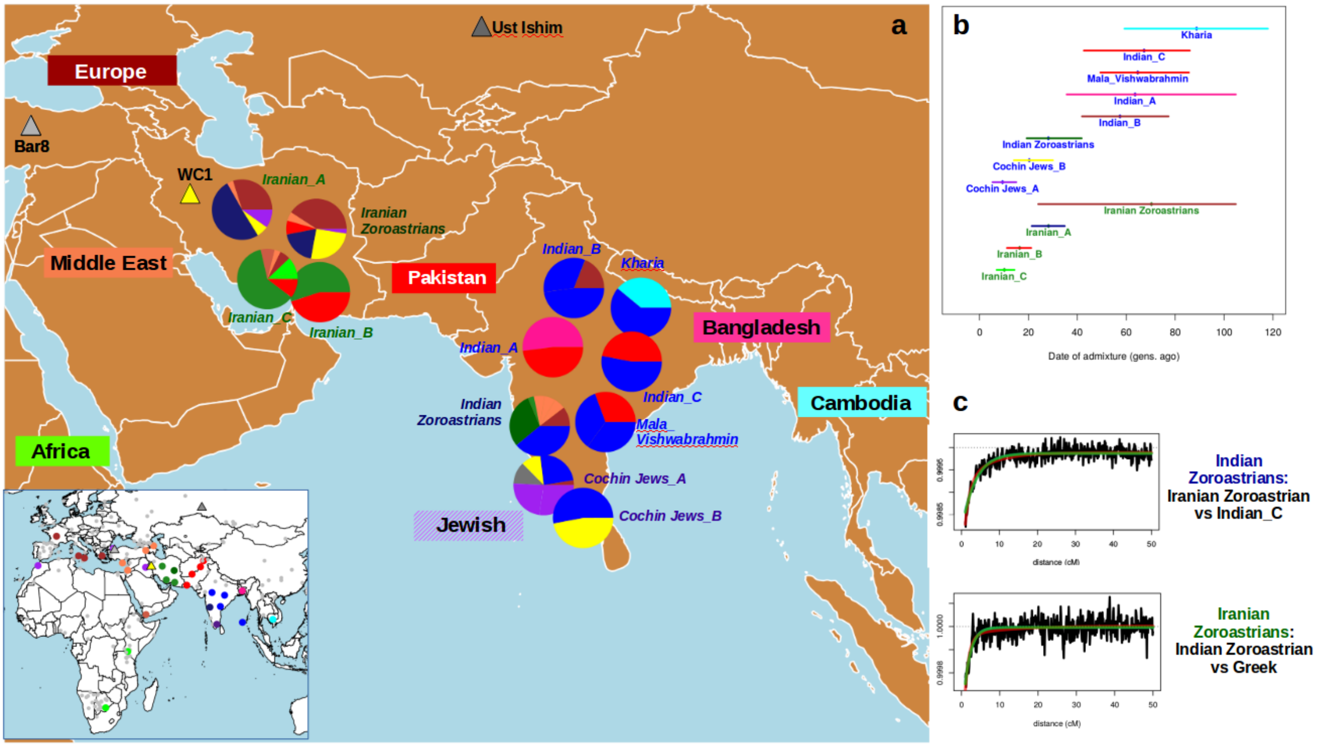

Figure S8: Maximum likelihood trees constructed with TreeMix between Iranian and Indian clusters (Indian_C, Indian Zoroastrians, Iranian_A and Iranian Zoroastrians) using Yoruba as an outgroup, for 0-1 (a-b) migration events. Edges show the direction of gene flow between populations.

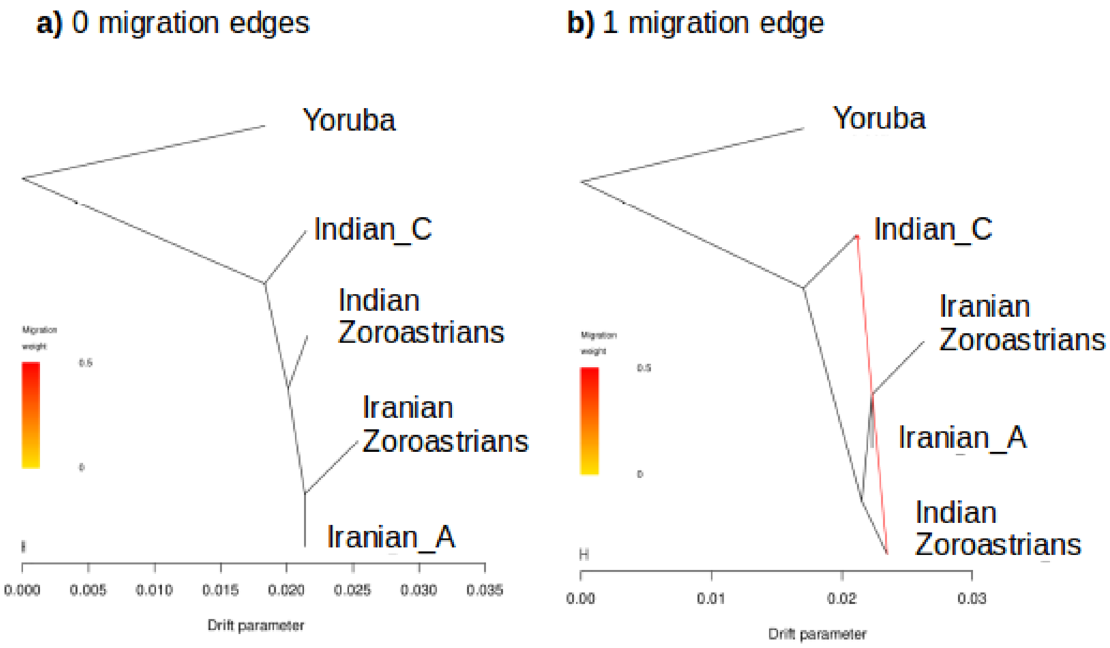

Figure S9: Residuals for the maximum likelihood trees constructed with TreeMix between Iranian and Indian clusters (Indian_C, Indian Zoroastrians, Iranian_A and Iranian Zoroastrians) using Yoruba as an outgroup, for 0-1 (a-b) migration events. Positive residuals indicate candidate populations for admixture events, as they are more closely related to each other in the data than predicted by the best-fitting tree.

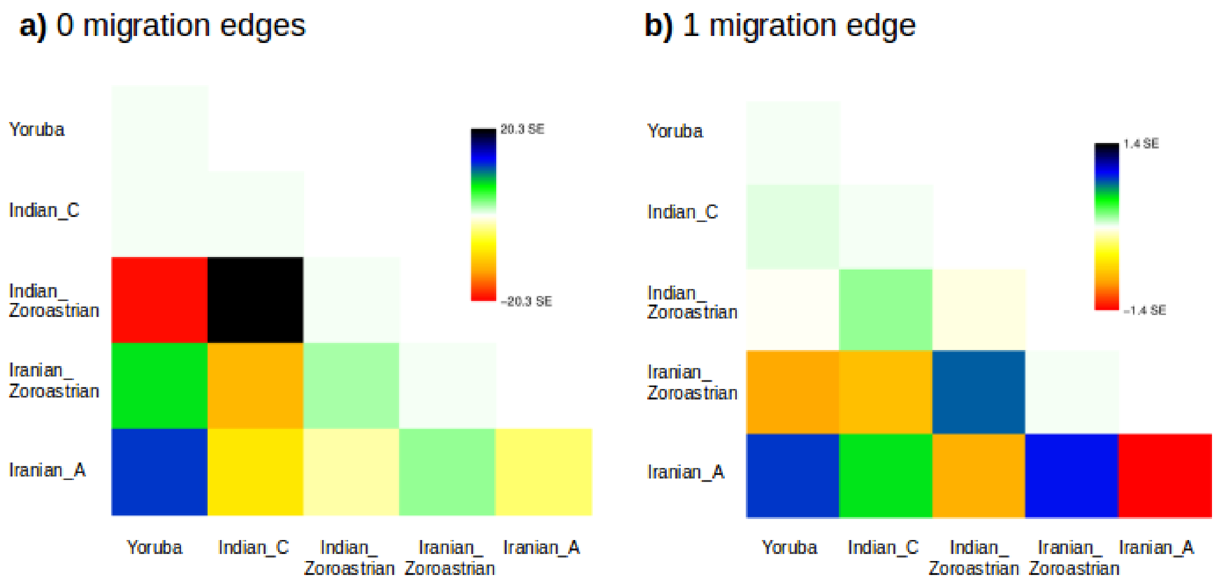

Figure S10: PCO plots of the 6 populations based on pairwise F_{ST} values of Yhg (above) and iMhg (below) frequencies, which summarise 88.5% and 93.2% of the variation, respectively.

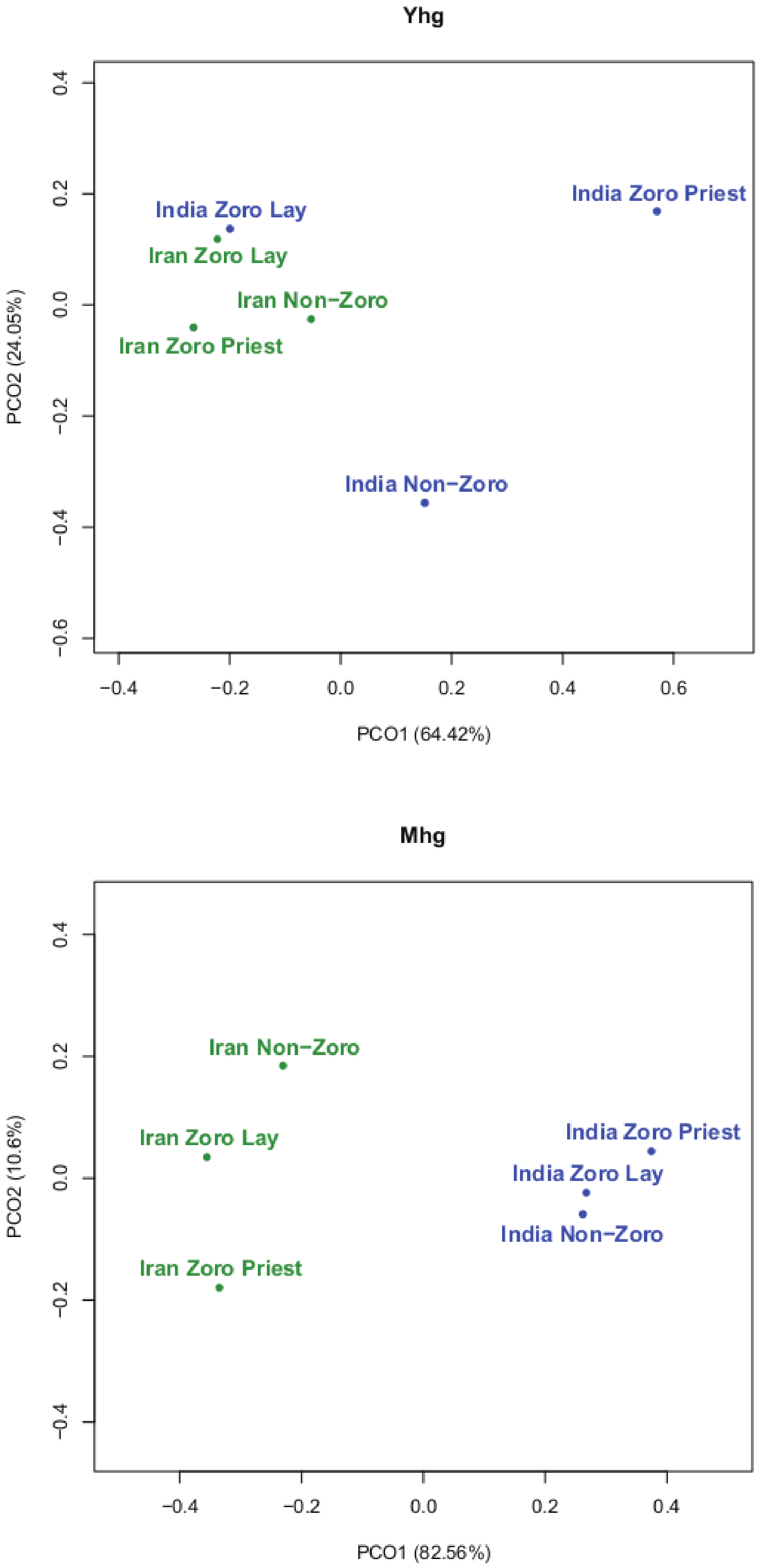

Figure S11: Y-chromosome haplogroups (Yhg), denoted according to the Y-chromosome consortium and the most recent nomenclature of ISOGG defined by the 12 UEP biallelic loci. Each node represents a Yhg; text on each branch indicates the mutation that defines the given nodes.

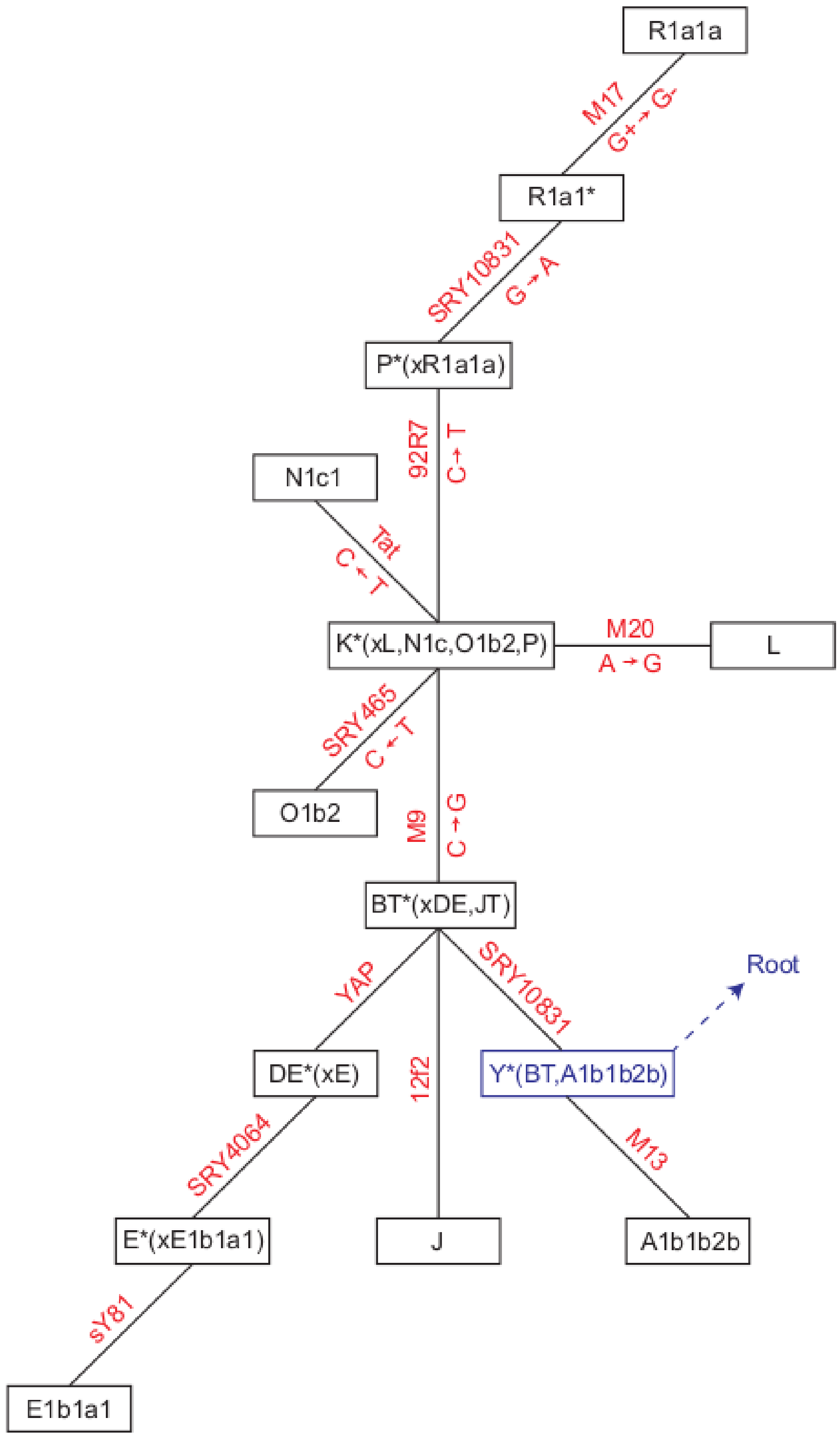

Figure S12. XP-EHH scores in Parsis (top) and Iranian Zoroastrians (bottom). Non-Zoroastrian Indian and Iranian populations were used as reference populations, respectively, for the XP-EHH test. The blue lines show the significance threshold values of 0.01% estimated from an empirical distribution of XP-EHH values (see Methods).

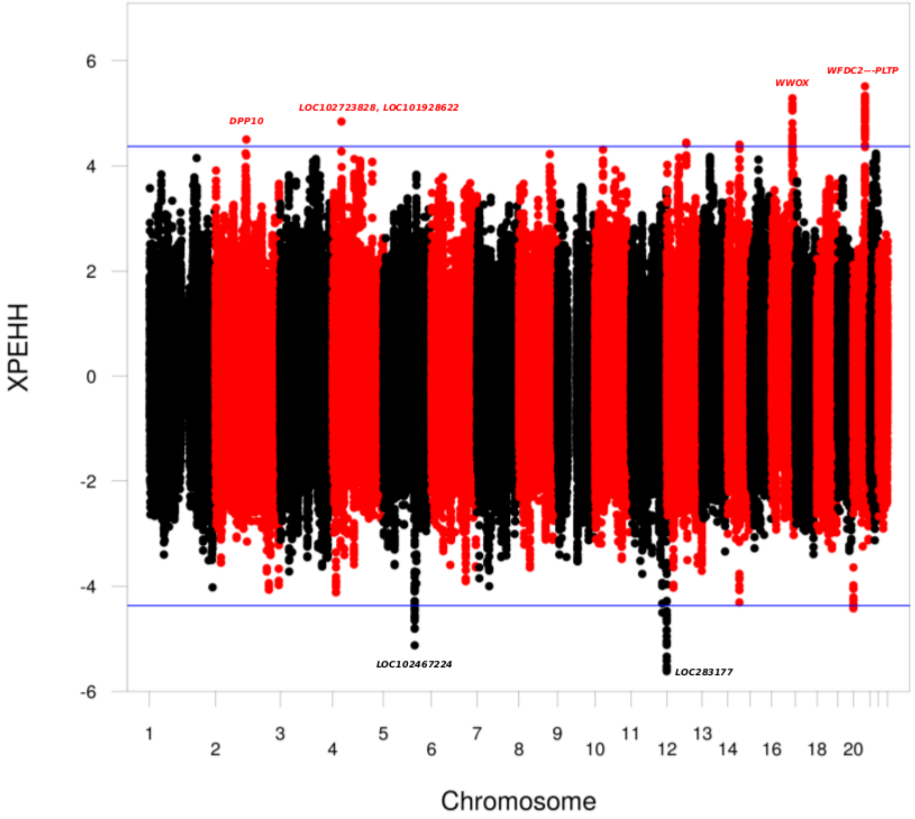

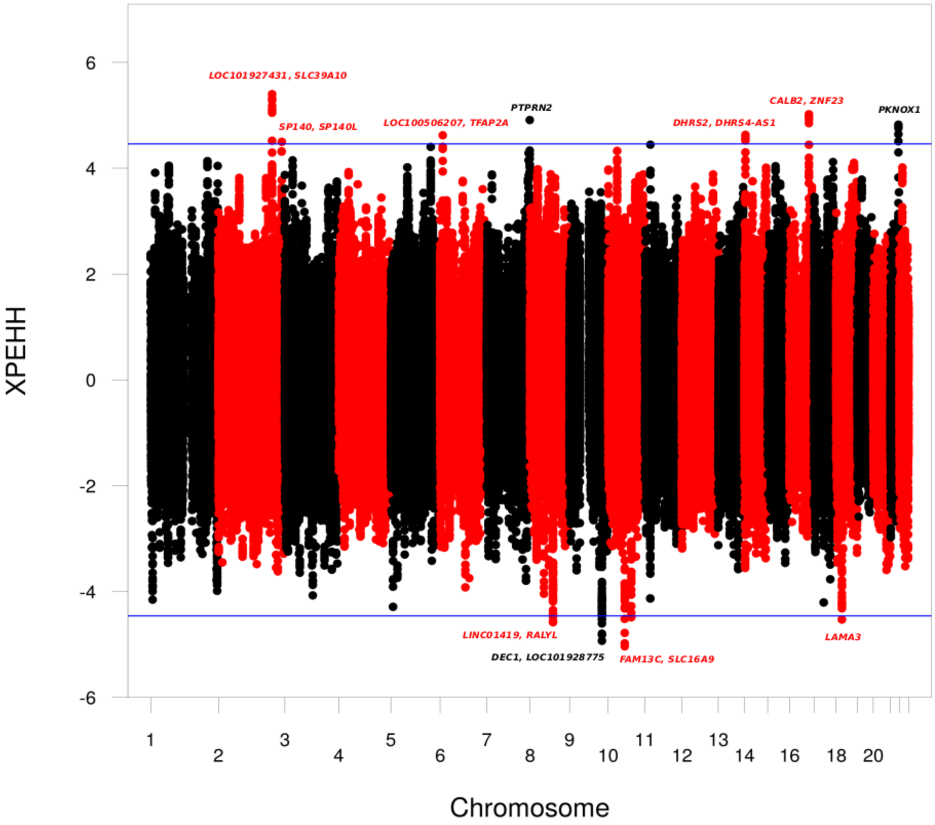

Figure S13: Empirical qqplots based on 100 permutations (x-axis), showing the mean values across permutations (x-axis) versus the real values (y-axis) observed for the Indian and Iranian populations.

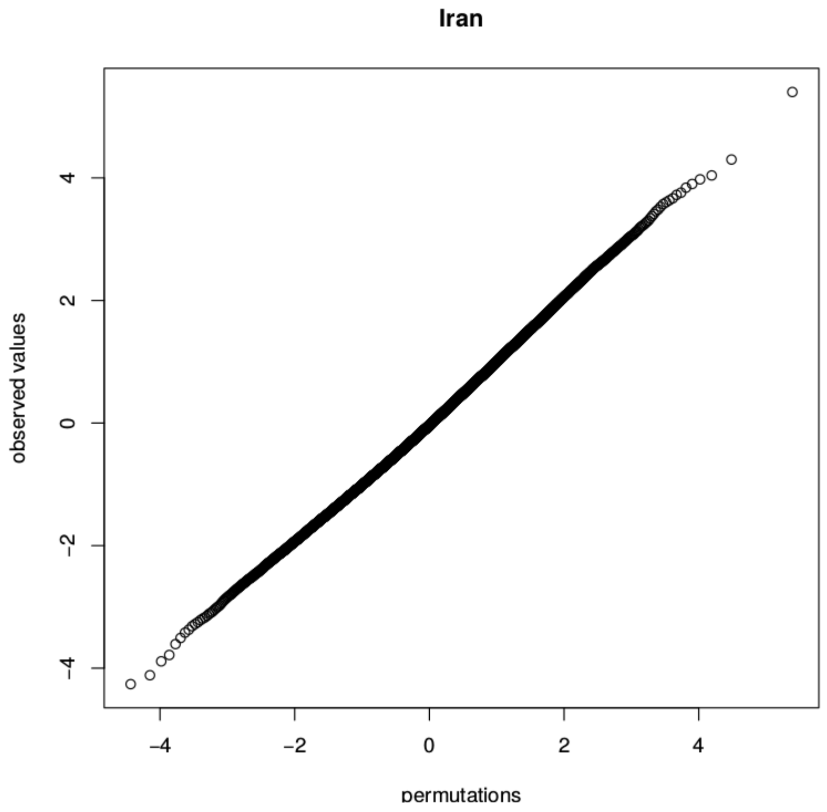

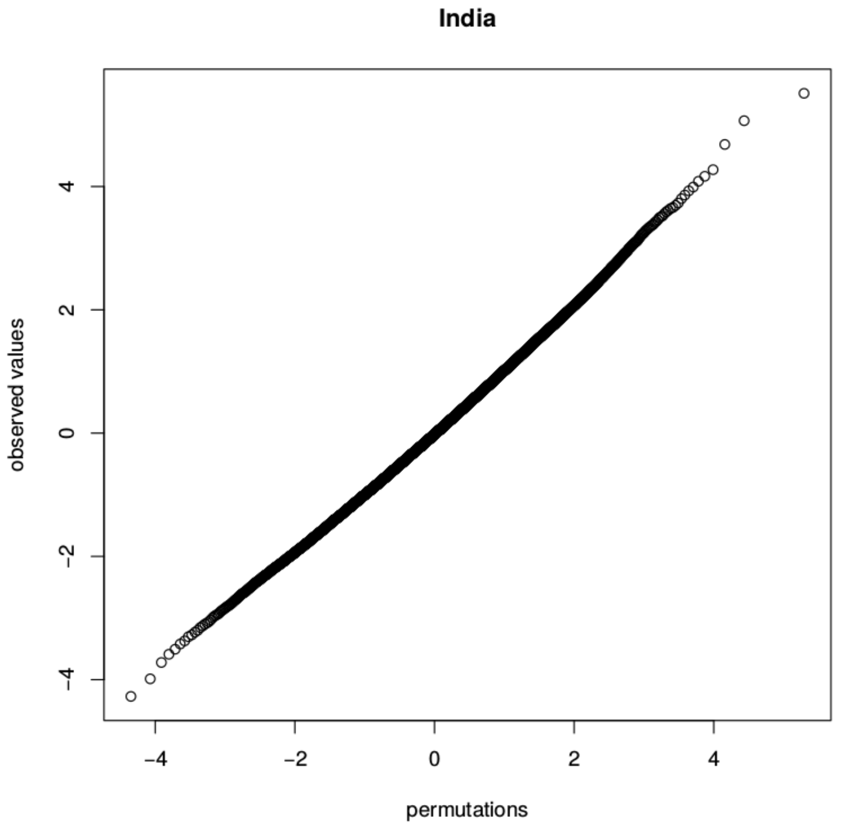

Supplemental Tables

Table S1. Description of the samples collected for this work and number of individuals included in each analysis.

Population	Collection place	Total samples	ChrY	mtDNA	Human Origins autosomal data
**Non-Zoroastrian from India**	-	49	41	46	12
**Non-Zoroastrian from Iran**	-	193	172	193	17
**Lay-Zoroastrian from India**	Bombay	56	54	53	7
	Dubai	8	8	8	0
	Navsari	37	37	37	4
	Surat	11	11	11	2
	UK	8	8	8	0
	Udwada	4	4	4	0
	Total	124	122	121	13
**Zoroastrian priests from India**	Bombay	13	12	12	0
	Dubai	10	10	10	0
	Navsari	20	20	20	0
	Surat	9	9	9	0
	UK	4	4	4	0
	Udwada	16	16	16	0
	Total	72	71	71	0
**Lay-Zoroastrian from Iran**	Isfahan	2	2	2	2
	Shiraz	8	7	8	6
	Tehran	19	18	18	9
	UK	1	0	1	0
	Yazd	50	49	50	12
	Total	80	76	79	29
**Zoroastrian priests from Iran**	Shiraz	1	1	1	0
	Tehran	3	3	3	0
	Yazd	4	4	4	0
	Total	8	8	8	0
**TOTAL**		526	490	518	71

Table S2. Number of individuals from each population label included in all the clusters inferred by fineSTRUCTURE.

Separate file: Table_S2.xls

Table S3. F_{ST}, TVD and F_{XY} between the Iranian, Indian, Pakistani and Armenian clusters

Separate file: Table_S3.xls

Table S4. f3 statistics estimated with ADMIXTOOLS, when using Parsis as a target group. Only |Z| scores >2 are shown.

Source1	Source2	Target	F_3	std. Err	Z
LBK	Vishwabrahmin	India_Zoroastrian	-0.002923	0.000733	-3.987
LBK	Mala	India_Zoroastrian	-0.002549	0.000761	-3.35
Lebanese_Christian	Kharia	India_Zoroastrian	-0.001434	0.000454	-3.161
Armenian	Kharia	India_Zoroastrian	-0.001397	0.000455	-3.073
NE1	Mala	India_Zoroastrian	-0.002263	0.00074	-3.06
Georgian_Megrels	Kharia	India_Zoroastrian	-0.00131	0.000455	-2.877
Bar8	Mala	India_Zoroastrian	-0.001909	0.000705	-2.708
NE1	Vishwabrahmin	India_Zoroastrian	-0.002017	0.000754	-2.675
LBK	Kharia	India_Zoroastrian	-0.002253	0.000914	-2.466
Turkish_Trabzon	Kharia	India_Zoroastrian	-0.001073	0.00046	-2.331
Assyrian	Kharia	India_Zoroastrian	-0.001037	0.000445	-2.33
Kharia	Druze	India_Zoroastrian	-0.00093	0.000403	-2.306
Iraqi_Jew	Kharia	India_Zoroastrian	-0.001088	0.000511	-2.131

Table S5. Admixture events inferred by GLOBETROTTER using modern populations (fineSTRUCTURE clusters) as surrogates under the "all donors painting". AdmDate=number of generations (date in years in brackets, using the formula 1950-28*(g+1) that assumes 28 years per generation g of admixture) since the admixture event occurred; S=source number; "prop" the proportion contributed by each source; "Source composition" indicates the single sampled group that best matches the inferred genetic make-up for the given source, plus in curly brackets a more detailed inference of the genetic make-up of the source (excluding groups inferred to contribute <=5% to this make-up).

**Target**	**AdmDate**	**AdmDate (min-max)**	**S**	**prop**	**Source composition**
Iranian_A	25 (1222 CE)	20-32 (1026 CE-1362 CE)	S1	0.49	Indian_Pakistani *{Turkish:0.1201 Kumyk:0.1226 Indian_Pakistani:0.2473}*
			S2	0.51	Lebanese_TurkishJew *{Armenian_Assyrian_Turkish:0.0565 Lebanese_TurkishJew:0.4535}*
Iranian_B	18 (1418 CE)	13-24 (1250 CE-558 CE)	S1	0.38	Pakistani *{Iranian_A:0.0656 Pakistani:0.3144}*
			S2	0.62	Iranian_C *{Iranian_C:0.62}*
Iranian_C	10 (1642 CE)	6-13 (1558 CE-1754 CE)	S1	0.14	Kikuyu *{Kikuyu:0.0908 Pathan:0.0838 Lebanese_TurkishJew:0.0996}*
			S2	0.86	Iranian_B *{Iranian_B:0.6468}*
Indian_Zoroastrian	27 (1166 CE)	17-38 (858 CE-1446 CE)	S1	0.36	Indian_A *{Indian_C:0.134 Indian_A:0.2054}*
			S2	0.64	Iranian_A *{Iranian_A:0.64}*
Indian_A	63 (158 CE)	40-107 (1074 BCE-802 CE)	S1	0.46	Mala_Vishwabrahmin *{Mala_Vishwabrahmin:0.46}*
			S2	0.54	Pathan*{Pathan:0.54}*
Indian_B	56 (354 CE)	40-72 (94 BCE-802 CE)	S1	0.28	Italian_Bergamo *{Tajik_Pomiri:0.0581 Italian_Bergamo:0.2219}*
			S2	0.72	Bengali_Bangladesh *{Indian_Pakistani:0.0898 Mala_Vishwabrahmin:0.1321 Indian_A:0.1566 Bengali_Bangladesh:0.3415}*
Indian_C	52 (466 CE)	30-82 (374 BCE-1082 CE)	S1	0.48	Mala_Vishwabrahmin *{Mala_Vishwabrahmin:0.48}*
			S2	0.52	Indian_Pakistani *{Indian_Pakistani:0.52}*
Kharia	84 (430 BCE)	60-107 (1074 BCE-242 CE)	S1	0.48	Cambodian *{Mala_Vishwabrahmin:0.0681 Cambodian:0.4104}*
			S2	0.52	Mala_Vishwabrahmin *{Indian_C:0.1818 Mala_Vishwabrahmin:0.3382}*
Mala_Vishwabrahmin	57 (326 CE)	42-80 (318 BCE-746 CE)	S1	0.45	Indian_Pakistani *{Indian_Pakistani:0.45}*
			S2	0.55	Bengali_Bangladesh *{Bengali_Bangladesh:0.55}*
CochinJew_A	26 (1194 CE)	10-39 (830 CE-1642 CE)	S1	0.47	Moroccan_Jew *{GeorgianJew_IraqiJew:0.1386 Moroccan_Jew:0.2063}*
			S2	0.53	CochinJew_B *{Moroccan_Jew:0.0532 GeorgianJew_IraqiJew:0.0619 CochinJew_B:0.354}*

CochinJew_B	12 (1586 CE)	6-22 (1306 CE-1754 CE)	S1	0.46	CochinJew_A *{CochinJew_A:0.46}*
			S2	0.54	Mala_Vishwabrahmin *{Bengali_Bangladesh:0.2617 Mala_Vishwabrahmin:0.2783}*
Indian_Pakistani	51 (494 CE)	33-64 (130 CE-998 CE)	S1	0.46	Mala_Vishwabrahmin *{Indian_A:0.0861 Mala_Vishwabrahmin:0.3297}*
			S2	0.54	Iranian_A *{Balochi:0.0818 Iranian_A:0.4582}*
Pathan	37 (886 CE)	24-53 (438 CE-1250 CE)	S1	0.46	Iranian_A *{Hungarian_Coriell:0.1124 Iranian_A:0.3476}*
			S2	0.54	Bengali_Bangladesh *{Indian_Pakistani:0.0859 Bengali_Bangladesh:0.4437}*
Kalash	64 (130 CE)	29-101 (906 BCE-1110 CE)	S1	0.34	Bengali_Bangladesh *{Bengali_Bangladesh:0.34}*
			S2	0.66	Tajik_Pomiri *{Pathan:0.1513 Tajik_Pomiri:0.5045}*
Hazara	22 (1306 CE)	20-25 (1222 CE-1362 CE)	S1	0.46	Pathan *{Pathan:0.46}*
			S2	0.54	Kalmyk *{Kalmyk:0.54}*
Burusho	47 (606 CE)	36-61 (214 CE-914 CE)	S1	0.49	Bengali_Bangladesh *{Bengali_Bangladesh:0.49}*
			S2	0.51	Tajik_Pomiri *{Tajik_Pomiri:0.51}*
Makrani	17 (1446 CE)	14-19 (1390 CE-1530 CE)	S1	0.05	Wambo
			S2	0.95	Pakistani *{Iranian_B:0.2814 Pakistani:0.6686}*
Pakistani	24 (1250 CE)	14-34 (970 CE-1530 CE)	S1	0.47	Makrani *{Makrani:0.47}*
			S2	0.53	Balochi *{Balochi:0.53}*
Balochi	14 (1530 CE)	5-20 (1362 CE-1782 CE)	S1	0.46	Pakistani *{Brahui:0.0818 Pakistani:0.3782}*
			S2	0.54	Indian_Pakistani *{Brahui:0.0574 Pathan:0.1773 Indian_Pakistani:0.2725}*
Brahui	18 (1418 CE)	11-24 (1250 CE-1614 CE)	S1	0.48	Balochi *{Balochi:0.48}*
			S2	0.52	Pakistani *{Pakistani:0.52}*
Armenian_Assyrian_ Turkish	29 (1110 CE)	20-39 (830 CE-1362 CE)	S1	0.47	Lebanese_TurkishJew *{Jordan_Palestinian_Syrian:0.0868 Lebanese_TurkishJew:0.3832}*
			S2	0.53	Turkish *{Turkish:0.4905}*

Table S6. Admixture events inferred by GLOBETROTTER using modern populations (fineSTRUCTURE clusters) and ancient samples as surrogates under the "all donors painting". AdmDate=number of generations (date in years in brackets, using the formula 1950-28*(g+1) that assumes 28 years per generation g of admixture) since the admixture event occurred; S=source number (E1 and E2 indicate two inferred admixture events occurring over approximately the same date interval); "prop" the proportion contributed by each source; "Source composition" indicates the single sampled group that best matches the inferred genetic make-up for the given source, plus in curly brackets a more detailed inference of the genetic make-up of the source (excluding groups inferred to contribute <=5% to this make-up).

**Target**	**AdmDate**	**AdmDate (min-max)**	**S**	**prop**	**Source composition**
Iranian_Zoroastrian	66 (740 CE)	42-89 (570 BCE-746 CE)	S1	0.33	Cypriot *{WC1:0.0542 Cypriot:0.1286 Croatian:0.1373}*
			S2	0.67	WC1 *{WC1:0.6338}*
Iranian_B	18 (1418 CE)	13-26 (1194 CE-1558 CE)	S1	0.39	Pakistani *{Czech:0.0695 Pakistani:0.3205}*
			S2	0.61	Iranian_C *{Iranian_C:0.5884}*
Iranian_C	10 (1642 CE)	6-12 (1586 CE-1754 CE)	S1	0.11	Luhya_Kenya *{Luhya_Kenya:0.0697}*
			S2	0.89	Iranian_B *{Yemeni_B:0.062 Bar8:0.0695 Pathan:0.095 Iranian_B:0.654}*
Indian_Zoroastrian	32 (1026 CE)	19-44 (690 CE-1390 CE)	S1	0.24	Indian_C *{Indian_C:0.24}*
			S2	0.76	WC1 *{Pathan:0.0658 WC1:0.6942}*
Indian_A	49 (550 CE)	29-77 (234 BCE-1110 CE)	S1	0.44	Mala_Vishwabrahmin *{Mala_Vishwabrahmin:0.44}*
			S2	0.56	Indian_Pakistani *{Indian_Pakistani:0.56}*
Indian_B	58 (298 CE)	41-72 (94 BCE-774 CE)	S1	0.24	Bulgarian *{Bulgarian:0.24}*
			S2	0.76	Bengali_Bangladesh *{Indian_C:0.0515 Indian_Pakistani:0.0957 Indian_A:0.1551 Bengali_Bangladesh:0.4577}*
Indian_C	44 (690 CE)	23-68 (18 CE-1278 CE)	S1	0.47	Mala_Vishwabrahmin *{Mala_Vishwabrahmin:0.47}*
			S2	0.53	Indian_B *{Indian_B:0.53}*
Kharia	84 (430 BCE)	60-108 (1102 BCE-242 CE)	S1	0.48	Cambodian *{Mala_Vishwabrahmin:0.0584 Cambodian:0.4216}*
			S2	0.52	Mala_Vishwabrahmin *{Mala_Vishwabrahmin:0.5177}*
Mala_Vishwabrahmin	70 (38 BCE)	43-104 (990 BCE-718 CE)	S1	0.31	UstIshim *{UstIshim:0.264}*
			S2	0.69	Indian_A *{Indian_C:0.1261 Indian_A:0.5639}*
CochinJew_A	29 (1110 CE)	7-40 (802 CE-1726 CE)	S1	0.47	CochinJew_B *{GeorgianJew_IraqiJew:0.0552 CochinJew_B:0.3585}*
			S2	0.53	Iranian_A *{Iranian_A:0.0942 GeorgianJew_IraqiJew:0.1612 Moroccan_Jew:0.2158}*
CochinJew_B	12 (1586CE)	6-21 (1334 CE-1754 CE)	S1	0.46	CochinJew_A *{CochinJew_A:0.46}*
			S2	0.54	Mala_Vishwabrahmin *{Bengali_Bangladesh:0.2617 Mala_Vishwabrahmin:0.2783}*

Indian_Pakistani	23 (1278 CE)	6-40 (802 CE-1754 CE)	S1	0.46	Bengali_Bangladesh *{Indian_B:0.0523 Indian_A:0.0752 Bengali_Bangladesh:0.3269}*
			S2	0.54	Iranian_B *{Balochi:0.1344 Iranian_B:0.1502 Pathan:0.2126}*
Pathan	47 (606 CE)	30-65 (102 CE-1082 CE)	S1	0.43	Turkish *{Turkish_36:0.4299}*
			S2	0.57	Indian_A *{Indian_Pakistani:0.0888 Indian_A:0.4026}*
Hazara	22 (1306 CE)	20-26 (1194 CE-1362 CE)	S1	0.46	Pathan *{Pathan:0.46}*
			S2	0.54	Kalmyk *{Kalmyk:0.54}*
Burusho	47 (606 CE)	35-60 (242 CE-942 CE)	S1	0.49	Bengali_Bangladesh *{Bengali_Bangladesh:0.49}*
			S2	0.51	Tajik_Pomiri *{Tajik_Pomiri:0.51}*
Makrani	16 (1474 CE)	13-20 (1362 CE-1558 CE)	S1	0.06	Luhya_Kenya *{Luhya_Kenya:0.06}*
			S2	0.94	Pakistani *{Iranian_C:0.1993 Pakistani:0.7407}*
Balochi	14 (1530 CE)	7-21 (1334 CE-1726 CE)	S1	0.46	Pakistani *{Brahui:0.0791 Pakistani:0.3807}*
			S2	0.54	Indian_Pakistani *{Brahui:0.0659 Pathan:0.1866 Indian_Pakistani:0.2874}*
Brahui	20 (1362 CE)	13-30 (1082 CE-1558 CE)	S1	0.45	Makrani *{Makrani:0.45}*
			S2	0.55	Balochi *{Balochi:0.55}*
Armenian_Assyrian_Turkish	61 (214 CE)	36-87 (514 BCE-914 CE)	S1	0.42	Iranian_A *{WC1:0.152 Iranian_A:0.2449}*
			S2	0.58	Lebanese_TurkishJew *{KK1:0.0608 Lebanese_TurkishJew:0.2265 Bar8:0.2639}*
Iranian_A	29 (1110 CE)	23-38 (858 CE-1278 CE)	E1.S1	0.48	WC1 *{Lebanese_TurkishJew:0.0692 WC1:0.379}*
			E1.S2	0.52	Turkish *{Lebanese_TurkishJew:0.0562 Turkish_36:0.4168}*
			E2.S1	0.48	Lebanese_TurkishJew *{Lebanese_TurkishJew:0.48}*
			E2.S2	0.52	Turkish *{WC1:0.094 Turkish:0.426}*
Pakistani	20 (1362 CE)	15-26 (1194 CE-1502 CE)	E1.S1	0.33	Indian_Pakistani *{Indian_Pakistani:0.315}*
			E1.S2	0.67	Makrani *{Brahui_19:0.2274 Makrani:0.4426}*
			E2.S1	0.43	Brahui *{Indian_Pakistani:0.0916 Brahui:0.3384}*
			E2.S2	0.57	Makrani *{Makrani:0.5335}*

Table S7. Admixture events for Indian and Iranian populations inferred by GLOBETROTTER using modern populations (fineSTRUCTURE clusters) and ancient samples as surrogates, under the "non Indian/Iranian donors painting". AdmDate=number of generations (date in years in brackets, using the formula 1950-28*(g+1) that assumes 28 years per generation g of admixture) since the admixture event occurred; S=source number; "prop" the proportion contributed by each source; "Source composition" indicates the single sampled group that best matches the inferred genetic make-up for the given source, plus in curly brackets a more detailed inference of the genetic make-up of the source (excluding groups inferred to contribute <=5% to this make-up).

Target	AdmDate	AdmDate (min-max)	S	Prop	Source composition
IranianZoroastrian	56 (354 CE)	16-94 (710 BCE-1474 CE)	S1	0.48	Greek_Coriell *{Greek_Coriell:0.4078}*
			S2	0.52	WC1 *{Pathan:0.0748 IndianZoroastrian:0.1909 WC1:0.254}*
Iranian_A	25 (1222 CE)	21-32 (1026 CE-1334 CE)	S1	0.43	Italian_EastSicilian *{Lebanese_TurkishJew:0.0984 Italian_EastSicilian:0.2987}*
			S2	0.57	IndianZoroastrian *{WC1:0.0634 IndianZoroastrian:0.5066}*
Iranian_B	18 (1418 CE)	13-25 (1222 CE-1558 CE)	S1	0.45	Balochi *{Balochi:0.45}*
			S2	0.55	Iranian_C *{Iranian_C:0.55}*
Iranian_C	10 (1642 CE)	7-13 (1558 CE-1726 CE)	S1	0.09	Tswana *{Tswana:0.0894}*
			S2	0.91	Iranian_B *{Italian_WestSicilian:0.0578 Yemeni:0.0746 Pathan:0.0972 Iranian_B:0.6112}*
IndianZoroastrian	30 (1082 CE)	21-45 (662 CE-1334 CE)	S1	0.39	Indian_C *{Indian_C:0.39}*
			S2	0.61	IranianZoroastrian *{Jordan_Palestinian_Syrian:0.0574 Cypriot:0.0589 Armenian_Assyrian_Turkish:0.0613 Greek_Coriell:0.104 IranianZoroastrian:0.2962}*
Indian_A	63 (158 CE)	38-89 (570 BCE-858 CE)	S1	0.48	Pathan *{Pathan:0.48}*
			S2	0.52	Bengali_Bangladesh_BEB *{Bengali_Bangladesh_BEB:0.52}*
Indian_B	58 (298 CE)	43-81 (346 BCE-718 CE)	S1	0.19	French *{French:0.1885}*
			S2	0.81	Indian_C *{Indian_A:0.3323 Indian_C:0.4777}*
Indian_C	69 (10 BCE)	51-89 (570 BCE-494 CE)	S1	0.47	Pathan *{Pathan:0.47}*
			S2	0.53	Mala_Vishwabrahmin *{Mala_Vishwabrahmin:0.53}*
CochinJew_A	9 (1670 CE)	5-15 (1502 CE-1782	S1	0.37	KuchinJew_B *{KuchinJew_B:0.0547 UstIshim:0.1107 Indian_A:0.1577}*

		CE)	S2	0.63	GeorgianJew_IraqiJew *{Indian_A:0.0712 WC1:0.0995 Moroccan_Jew:0.2227 GeorgianJew_IraqiJew_16:0.2234}*
CochinJew_B	19 (1390 CE)	12-33 (998 CE -1586 CE)	S1	0.47	WC1 *{WC1:0.47}*
			S2	0.53	Mala_Vishwabrahmin *{Mala_Vishwabrahmin:0.53}*
Mala_Vishwabrahmin	66 (74 CE)	43-82 (374 BCE-718 CE)	S1	0.32	Onge *{Onge_11:0.32}*
			S2	0.68	Indian_C *{KuchinJew_B:0.0864 Pathan:0.2798 Indian_C:0.3138}*
Kharia	89 (570 BCE)	67-122 (1484 BCE -46 CE)	S1	0.39	Cambodian *{Cambodian:0.39}*
			S2	0.61	Mala_Vishwabrahmin *{Mala_Vishwabrahmin:0.61}*

Table S8. Admixture events inferred by GLOBETROTTER for single outlier Iranian Zoroastrians using all modern populations (fineSTRUCTURE clusters) as surrogates under the "all donors painting", also including results for the groups they cluster with (i.e. Iranian_B, which includes YZ020 and Lebanese_TurkishJew, which includes YZ024). Only results for null.ind 1 analyses are shown. AdmDate=number of generations (date in years in brackets, using the formula 1950-28*(g+1) that assumes 28 years per generation g of admixture) since the admixture event occurred; S=source number; "prop" the proportion contributed by each source; "Source composition" indicates the single sampled group that best matches the inferred genetic make-up for the given source, plus in curly brackets a more detailed inference of the genetic make-up of the source (excluding groups inferred to contribute <=5% to this make-up).

**Target**	**AdmDate**	**S**	**prop**	**Source composition**
**Iranian_Zoroastrian_YZ020**	18 (1418 CE)	S1	0.23	Makrani *{Makrani:0.1117 Pakistani:0.1057}*
		S2	0.77	Iranian_A *{Iranian_A: 0.3157 Lebanese_TurkishJew: 0.1266 Brahui: 0.0540}*
**Iranian_B**	18	S1	0.38	Pakistani {*Iranian_A:0.0656 Pakistani:0.3144*}
	(1418 CE)		0.62	Iranian_C {*Iranian_C:0.62*}
**Iranian_Zoroastrian_YZ024**	88 (542 BCE)	S1	0.01	Mozabite
		S2	0.99	Ashkenazi_Jew *{Ashkenazi_Jew:0.989}*
**Lebanese_TurkishJew**	Date1: 13 (1558 CE)	S1	0.03	Yemeni_B_2
		S2	0.97	Turkish {*Ashkenazi_Jew: 0.065 Greek_Coriell: 0.078 Cypriot: 0.092 Jordan_Palestinian_Syrian: 0.103 Italian_EastSicilian: 0.115 Iranian_A:0.206*}
	Date2: 57 (326 CE)	S1	0.06	Masai_Ayodo
		S2	0.94	Turkish{*Armenian_Assyrian_Turkish:0.057435134674136 Cypriot:0.056 Italian_Bergamo: 0.056 Greek_Coriell: 0.069 Jordan_Palestinian_Syrian: 0.074 Turkish: 0.089 Iranian_A:0.248*}

Table S9. Y-chromosome haplogroup frequencies and gene diversities.

Haplogroup	Iranian Non Zoroastrian	Indian Non Zoroastrian	Zoroastrian - India		Zoroastrian - Iran	
			Lay	Priest	Lay	Priest
**P*(xR1a1a)**	0.169	0.122	0.238	0.310	0.171	0.125
**N1c1**	0.012	0.000	0.000	0.000	0.000	0.000
**BT*(xDE,JT)**	0.140	0.268	0.057	0.085	0.053	0.000
**E*(xE1b1a1)**	0.110	0.000	0.057	0.014	0.118	0.000
**K*(xL,N1c,O1b2,P)**	0.041	0.049	0.016	0.000	0.039	0.250
**L**	0.047	0.098	0.025	0.549	0.013	0.000
**R1a1a**	0.105	0.317	0.057	0.000	0.053	0.125
**J**	0.378	0.146	0.549	0.042	0.553	0.500
**Total N**	172	41	122	71	76	8
**H (SE)**	0.787 (0.020)	0.799 (0.032)	0.636 (0.038)	0.602 (0.042)	0.653 (0.051)	0.750 (0.139)

Table S10. MtDNA macrohaplogroup frequencies and gene diversities.

Macro-haplogroups	Iranian Non Zoroastrian	Indian Non Zoroastrian	Zoroastrian – India		Zoroastrian – Iran	
			Lay	Priest	Lay	Priest
**A**	0.005	0.000	0.000	0.000	0.000	0.000
**B**	0.026	0.000	0.000	0.000	0.000	0.000
**C**	0.005	0.000	0.000	0.000	0.000	0.000
**D**	0.005	0.022	0.008	0.028	0.000	0.000
**H**	0.290	0.196	0.223	0.197	0.291	0.250
**I**	0.031	0.000	0.000	0.000	0.000	0.000
**J**	0.130	0.022	0.008	0.000	0.000	0.000
**K**	0.078	0.000	0.000	0.000	0.000	0.000
**L**	0.000	0.043	0.083	0.056	0.013	0.000
**M**	0.026	0.391	0.430	0.549	0.013	0.000
**N**	0.021	0.087	0.008	0.000	0.051	0.125
**P**	0.010	0.000	0.000	0.000	0.000	0.000
**R**	0.026	0.065	0.066	0.042	0.000	0.000
**S**	0.005	0.000	0.000	0.000	0.000	0.000
**T**	0.073	0.000	0.050	0.014	0.532	0.625
**U**	0.202	0.109	0.107	0.113	0.051	0.000
**W**	0.036	0.065	0.000	0.000	0.000	0.000
**X**	0.026	0.000	0.017	0.000	0.051	0.000
**Y**	0.005	0.000	0.000	0.000	0.000	0.000
**Total N**	193	46	121	71	79	8
**H (SE)**	0.676 (0.059)	0.819 (0.022)	0.661 (0.036)	0.581 (0.054)	0.674 (0.044)	0.821 (0.1)

Table S11. XPEHH results for Indian Zoroastrians vs Indian non-Zoroastrians. SNPs below and above quantiles 0.0001 and 0.9999 of the empirical distribution respectively (see methods), including the genes within those regions, or the flanking genes in the case of intergenic SNPs.

**SNP**	**Chr**	**BP**	**Location**	**Gene**	**XPEHH**
rs4559034	chr5	117451628	ncRNA_intronic	*LOC102467224*	-4.41826
rs972264	chr5	117527579	ncRNA_intronic	*LOC102467224*	-4.41874
rs17432160	chr5	117528933	ncRNA_intronic	*LOC102467224*	-4.65247
rs2061883	chr5	117530755	ncRNA_intronic	*LOC102467224*	-4.59379
rs2061882	chr5	117531223	ncRNA_intronic	*LOC102467224*	-4.59379
rs28566849	chr5	117533566	ncRNA_intronic	*LOC102467224*	-4.59379
rs1382704	chr5	117534903	ncRNA_intronic	*LOC102467224*	-4.59415
rs1479180	chr5	117538740	ncRNA_intronic	*LOC102467224*	-4.59415
rs34206135	chr5	117549966	ncRNA_intronic	*LOC102467224*	-4.59174
rs6883098	chr5	117560263	ncRNA_intronic	*LOC102467224*	-4.81066
rs11955483	chr5	117561687	ncRNA_intronic	*LOC102467224*	-5.12559
rs11744859	chr5	117569374	ncRNA_intronic	*LOC102467224*	-4.79995
rs11748941	chr5	117570688	ncRNA_intronic	*LOC102467224*	-4.54282
rs61250898	chr5	117575717	ncRNA_intronic	*LOC102467224*	-4.46385
rs77543824	chr5	117579383	ncRNA_intronic	*LOC102467224*	-4.46385
rs11216547	chr11	117666413	intronic	*DSCAML1*	-4.50502
rs10894845	chr11	134448470	intergenic	*LOC283177*	-4.63119
rs9919607	chr11	134449247	intergenic	*LOC283177*	-4.60421
rs7926027	chr11	134466656	intergenic	*LOC283177*	-4.68149
rs3019685	chr11	134485352	intergenic	*LOC283177*	-5.61552
rs2000858	chr11	134485458	intergenic	*LOC283177*	-5.41945
rs2097112	chr11	134486859	intergenic	*LOC283177*	-5.34052
rs2187463	chr11	134493110	intergenic	*LOC283177*	-5.52537
rs3017983	chr11	134493977	intergenic	*LOC283177*	-5.57282
rs3019668	chr11	134495874	intergenic	*LOC283177*	-4.95463
rs1944878	chr11	134498120	intergenic	*LOC283177*	-4.8481
rs3017965	chr11	134506955	intergenic	*LOC283177*	-5.04921
rs7939984	chr11	134507055	intergenic	*LOC283177*	-5.11849
rs3017963	chr11	134508438	intergenic	*LOC283177*	-5.07565
rs3019652	chr11	134508966	intergenic	*LOC283177*	-4.83669
rs113043921	chr11	134512512	intergenic	*LOC283177*	-4.83669
rs3019659	chr11	134513448	intergenic	*LOC283177*	-4.64051
rs949107	chr11	134536000	intergenic	*LOC283177*	-4.47438
rs11601492	chr11	134539450	intergenic	*LOC283177*	-4.54736
rs3017995	chr11	134539716	intergenic	*LOC283177*	-4.56322
rs1939728	chr11	134547554	intergenic	*LOC283177*	-4.538
rs6086704	chr20	947150	intronic	*RSPO4*	-4.42287

rs2223962	chr20	952218	intronic	*RSPO4*	-4.38887
rs4297887	chr2	115887977	intronic	*DPP10*	4.5017
rs35003803	chr4	32955931	intergenic	*LOC102723828,LOC101928622*	4.84255
rs1400145	chr12	73306993	intergenic	*TRHDE,LOC101928137*	4.44321
rs11179461	chr12	73317554	intergenic	*TRHDE,LOC101928137*	4.41985
rs12306579	chr12	73317695	intergenic	*TRHDE,LOC101928137*	4.41985
rs113062692	chr12	73321006	intergenic	*TRHDE,LOC101928137*	4.41985
rs2057130	chr14	64961479	intronic	*ZBTB25*	4.40508
rs9939675	chr16	78398705	intronic	*WWOX*	4.81195
rs4888787	chr16	78399140	intronic	*WWOX*	4.68308
rs112927307	chr16	78399322	intronic	*WWOX*	4.68308
rs72796072	chr16	78416119	intronic	*WWOX*	4.46482
rs72796083	chr16	78419515	intronic	*WWOX*	4.46482
rs2667545	chr16	78502389	intronic	*WWOX*	4.51242
rs3115955	chr16	78503595	intronic	*WWOX*	4.51242
rs12598729	chr16	78504236	intronic	*WWOX*	4.54594
rs2738680	chr16	78505408	intronic	*WWOX*	4.58225
rs2738681	chr16	78505709	intronic	*WWOX*	4.55812
rs73574998	chr16	78506089	intronic	*WWOX*	4.55812
rs8051225	chr16	78512313	intronic	*WWOX*	5.28383
rs11643648	chr16	78514583	intronic	*WWOX*	5.28383
rs2667562	chr16	78515158	intronic	*WWOX*	5.28383
rs2667569	chr16	78517377	intronic	*WWOX*	5.28383
rs2667570	chr16	78517684	intronic	*WWOX*	5.28383
rs2345998	chr16	78519416	intronic	*WWOX*	5.28383
rs1540757	chr16	78519689	intronic	*WWOX*	5.12212
rs2667579	chr16	78521339	intronic	*WWOX*	5.12212
rs2738700	chr16	78521500	intronic	*WWOX*	5.12212
rs2738701	chr16	78521997	intronic	*WWOX*	5.12212
rs2738704	chr16	78522184	intronic	*WWOX*	5.12212
rs28505640	chr16	78525030	intronic	*WWOX*	5.12212
rs2738714	chr16	78525954	intronic	*WWOX*	5.12212
rs62036391	chr16	78526959	intronic	*WWOX*	5.12212
rs2738721	chr16	78528446	intronic	*WWOX*	5.05292
rs2738727	chr16	78530734	intronic	*WWOX*	5.05292
rs2667589	chr16	78530885	intronic	*WWOX*	5.05292
rs17639042	chr16	78533357	intronic	*WWOX*	4.52346
rs7205635	chr16	78536121	intronic	*WWOX*	4.52346
rs12443611	chr16	78537146	intronic	*WWOX*	5.18486
rs1877281	chr16	78539415	intronic	*WWOX*	4.57852
rs1882958	chr16	78541355	intronic	*WWOX*	4.57852
rs58211226	chr16	78542655	intronic	*WWOX*	4.57852

rs11645676	chr16	78563832	intronic	*WWOX*	4.45776
rs2738498	chr16	78568270	intronic	*WWOX*	4.38329
rs9635580	chr16	78572354	intronic	*WWOX*	4.39638
rs1883519	chr20	44130596	intergenic	*WFDC2,SPINT3*	4.67317
rs6032249	chr20	44131517	intergenic	*WFDC2,SPINT3*	4.6697
rs6032250	chr20	44131736	intergenic	*WFDC2,SPINT3*	4.6697
rs1546889	chr20	44132906	intergenic	*WFDC2,SPINT3*	4.6697
rs909878	chr20	44135521	intergenic	*WFDC2,SPINT3*	4.59175
rs8124864	chr20	44144478	upstream	*SPINT3*	4.67909
rs6017595	chr20	44154532	intergenic	*SPINT3,WFDC6*	4.69295
rs146198026	chr20	44160213	intergenic	*SPINT3,WFDC6*	4.69295
rs3746593	chr20	44162849	UTR3	*WFDC6*	4.69295
rs6032274	chr20	44163988	intronic	*WFDC6*	4.75832
rs6094159	chr20	44164091	intronic	*WFDC6*	4.5579
rs6032331	chr20	44196310	intronic	*WFDC8*	4.66286
rs6104229	chr20	44197259	intronic	*WFDC8*	4.39378
rs6017628	chr20	44204536	intronic	*WFDC8*	4.39378
rs3091718	chr20	44216791	intergenic	*WFDC8,WFDC9*	4.39378
rs3091929	chr20	44216911	intergenic	*WFDC8,WFDC9*	4.70231
rs4812922	chr20	44217726	intergenic	*WFDC8,WFDC9*	4.70231
rs75511654	chr20	44218160	intergenic	*WFDC8,WFDC9*	4.70231
rs2425707	chr20	44224978	intergenic	*WFDC8,WFDC9*	5.04173
rs6032368	chr20	44225010	intergenic	*WFDC8,WFDC9*	5.04173
rs2425708	chr20	44226454	intergenic	*WFDC8,WFDC9*	5.20786
rs2425710	chr20	44227493	intergenic	*WFDC8,WFDC9*	5.03559
rs112947150	chr20	44227993	intergenic	*WFDC8,WFDC9*	5.03559
rs73131022	chr20	44228451	intergenic	*WFDC8,WFDC9*	5.03559
rs2235600	chr20	44238299	intronic	*WFDC9*	4.84654
rs978778	chr20	44243376	intronic	*WFDC9*	4.84654
rs1487327	chr20	44247986	intronic	*WFDC9*	5.01467
rs76995892	chr20	44251340	intronic	*WFDC9*	5.01467
rs3091694	chr20	44251629	intronic	*WFDC9*	5.17645
rs1157672	chr20	44258743	intronic	*WFDC10A,WFDC9*	5.17645
rs2272961	chr20	44259549	exonic	*WFDC10A*	4.9251
rs78065887	chr20	44278320	intronic	*WFDC11*	4.9251
rs4810461	chr20	44278445	intronic	*WFDC11*	4.93388
rs1487318	chr20	44295811	intronic	*WFDC11*	4.96159
rs6073854	chr20	44309605	intergenic	*WFDC11,WFDC10B*	4.89063
rs1013562	chr20	44310744	intergenic	*WFDC11,WFDC10B*	4.85908
rs6065861	chr20	44311056	intergenic	*WFDC11,WFDC10B*	4.85908
rs4810465	chr20	44311354	intergenic	*WFDC11,WFDC10B*	4.85908
rs2281211	chr20	44312858	downstream	*WFDC10B*	4.85908

rs2072974	chr20	44313401	UTR3	*WFDC10B*	4.85908
rs67538107	chr20	44327802	intronic	*WFDC10B*	5.01164
rs6073862	chr20	44331705	intronic	*WFDC10B,WFDC13*	4.62491
rs386196	chr20	44332873	intronic	*WFDC10B,WFDC13*	4.62491
rs6032449	chr20	44336716	UTR3	*WFDC13*	4.73825
rs6017653	chr20	44336924	UTR3	*WFDC13*	4.92872
rs232263	chr20	44350728	upstream	*SPINT4*	4.87487
rs11697000	chr20	44350763	upstream	*SPINT4*	4.87487
rs1386505	chr20	44351421	intronic	*SPINT4*	5.31282
rs1386504	chr20	44351515	intronic	*SPINT4*	5.21524
rs2200586	chr20	44351554	intronic	*SPINT4*	5.51207
rs6017666	chr20	44352307	intronic	*SPINT4*	5.18131
rs73908195	chr20	44353900	intronic	*SPINT4*	5.18131
rs761810	chr20	44355180	downstream	*SPINT4*	5.18131
rs232258	chr20	44356194	intergenic	*SPINT4,WFDC3*	5.20808
rs462038	chr20	44360467	intergenic	*SPINT4,WFDC3*	5.07953
rs459548	chr20	44366500	intergenic	*SPINT4,WFDC3*	5.00341
rs463793	chr20	44368325	intergenic	*SPINT4,WFDC3*	5.00341
rs455451	chr20	44373893	intergenic	*SPINT4,WFDC3*	5.00341
rs382515	chr20	44380316	intergenic	*SPINT4,WFDC3*	5.00341
rs405247	chr20	44380347	intergenic	*SPINT4,WFDC3*	5.00341
rs454874	chr20	44380471	intergenic	*SPINT4,WFDC3*	5.00341
rs17365711	chr20	44395664	intergenic	*SPINT4,WFDC3*	5.00341
rs2664529	chr20	44402869	UTR3	*WFDC3*	5.06458
rs6130930	chr20	44410837	intronic	*WFDC3*	5.24862
rs7263437	chr20	44410887	intronic	*WFDC3*	5.24862
rs567348	chr20	44410933	intronic	*WFDC3*	5.23038
rs3746493	chr20	44418564	exonic	*WFDC3*	5.21809
rs12480813	chr20	44425776	intronic	*DNTTIP1*	5.21809
rs4812964	chr20	44435334	intronic	*DNTTIP1*	5.21809
rs399672	chr20	44438299	intronic	*DNTTIP1*	5.25915
rs6104355	chr20	44445676	downstream	*UBE2C*	5.23641
rs145338460	chr20	44446276	downstream	*UBE2C*	5.23641
rs80177750	chr20	44448315	intergenic	*UBE2C,TNNC2*	5.23641
rs4629	chr20	44452697	exonic	*TNNC2*	5.09931
rs437122	chr20	44454978	intronic	*TNNC2*	5.32993
rs116367119	chr20	44455890	UTR5	*TNNC2*	5.32993
rs58751125	chr20	44466211	intronic	*SNX21*	5.32993
rs58334571	chr20	44468894	intronic	*SNX21*	5.32993
rs1057275	chr20	44471276	UTR3	*SNX21*	5.32993
rs73291275	chr20	44477528	intronic	*ACOT8*	5.32993
rs3746495	chr20	44479794	intronic	*ACOT8*	5.32993

rs6124749	chr20	44481871	intronic	*ACOT8*	5.10242
rs1967656	chr20	44488199	intronic	*ZSWIM3*	5.10242
rs6104374	chr20	44498391	intronic	*ZSWIM3*	5.08311
rs78044163	chr20	44503454	intronic	*ZSWIM3*	5.08311
rs2903808	chr20	44505973	exonic	*ZSWIM3*	5.06791
rs3746525	chr20	44507385	UTR3	*ZSWIM3*	5.06791
rs3746524	chr20	44507502	UTR3	*ZSWIM3*	5.06791
rs742034	chr20	44522005	intronic	*CTSA*	5.09269
rs4810476	chr20	44522594	intronic	*CTSA*	5.01164
rs115172363	chr20	44527765	intronic	*PLTP*	5.01164
rs394643	chr20	44540178	intronic	*PLTP*	4.52057
rs4810479	chr20	44545048	intergenic	*PLTP,PCIF1*	4.96894

Table S12. XPEHH results for Iranian Zoroastrians vs Iranian non-Zoroastrians. SNPs below and above quantiles 0.0001 and 0.9999 of the empirical distribution respectively (see methods), including the genes within those regions, or the flanking genes in the case of intergenic SNPs.

SNP	Chr	BP	Location	Gene	XPEHH
rs3857976	chr8	84341097	intergenic	*LINC01419,RALYL*	-4.57514
rs2053744	chr8	84374321	intergenic	*LINC01419,RALYL*	-4.47158
rs73282932	chr8	84382174	intergenic	*LINC01419,RALYL*	-4.55186
rs13253167	chr8	84425132	intergenic	*LINC01419,RALYL*	-4.58144
rs10817815	chr9	118451020	intergenic	*DEC1,LOC101928775*	-4.93139
rs72764742	chr9	118452085	intergenic	*DEC1,LOC101928775*	-4.79559
rs73654588	chr9	118453824	intergenic	*DEC1,LOC101928775*	-4.81785
rs7863684	chr9	118455827	intergenic	*DEC1,LOC101928775*	-4.6041
rs10982901	chr9	118499474	intergenic	*DEC1,LOC101928775*	-4.52498
rs4978653	chr9	118500105	intergenic	*DEC1,LOC101928775*	-4.52498
rs384626	chr10	61136215	intergenic	*FAM13C,SLC16A9*	-4.51628
rs78451775	chr10	61136963	intergenic	*FAM13C,SLC16A9*	-4.51628
rs11006458	chr10	61139869	intergenic	*FAM13C,SLC16A9*	-5.03629
rs513817	chr10	61140376	intergenic	*FAM13C,SLC16A9*	-4.97856
rs397014	chr10	61142485	intergenic	*FAM13C,SLC16A9*	-4.78198
rs7095696	chr10	85076518	intergenic	*NRG3,GHITM*	-4.47023
rs10886214	chr10	85127739	intergenic	*NRG3,GHITM*	-4.4687
rs10749261	chr10	85131723	intergenic	*NRG3,GHITM*	-4.48617
rs76706594	chr10	85134722	intergenic	*NRG3,GHITM*	-4.48617
rs141563527	chr10	85135411	intergenic	*NRG3,GHITM*	-4.48617
rs4800511	chr18	21360740	intronic	*LAMA3*	-4.52881
rs1112378	chr18	21384324	intronic	*LAMA3*	-4.52881
rs10497759	chr2	196277506	intergenic	*LOC101927431,SLC39A10*	4.51925
rs11692599	chr2	196278883	intergenic	*LOC101927431,SLC39A10*	4.51925
rs7559362	chr2	196319300	intergenic	*LOC101927431,SLC39A10*	5.04777
rs4850618	chr2	196321390	intergenic	*LOC101927431,SLC39A10*	5.06592
rs12693770	chr2	196326146	intergenic	*LOC101927431,SLC39A10*	5.32369
rs10497775	chr2	196326799	intergenic	*LOC101927431,SLC39A10*	5.27907
rs1500604	chr2	196330222	intergenic	*LOC101927431,SLC39A10*	5.40105
rs74532088	chr2	196332769	intergenic	*LOC101927431,SLC39A10*	5.17855
rs4850619	chr2	196333358	intergenic	*LOC101927431,SLC39A10*	5.11385
rs6434780	chr2	196335282	intergenic	*LOC101927431,SLC39A10*	5.08859
rs2720162	chr2	231629360	intronic	*CAB39*	4.49913
rs192998154	chr2	231631709	intronic	*CAB39*	4.49913
rs1322826	chr6	10082402	intergenic	*LOC100506207,TFAP2A*	4.62267

rs10277232	chr7	157959620	intronic	*PTPRN2*	4.91094
rs8020506	chr14	24345479	intergenic	*DHRS2,DHRS4-AS1*	4.63184
rs10139869	chr14	24346429	intergenic	*DHRS2,DHRS4-AS1*	4.54983
rs111706690	chr14	24352813	intergenic	*DHRS2,DHRS4-AS1*	4.54983
rs74036713	chr14	24354181	intergenic	*DHRS2,DHRS4-AS1*	4.54983
rs11158422	chr14	24370428	intergenic	*DHRS2,DHRS4-AS1*	4.58538
rs1957684	chr14	24375437	intergenic	*DHRS2,DHRS4-AS1*	4.52319
rs12444419	chr16	71408475	intronic	*CALB2*	4.97967
rs8046337	chr16	71408527	intronic	*CALB2*	4.97967
rs6499515	chr16	71408940	intronic	*CALB2*	4.98128
rs80317611	chr16	71412682	intronic	*CALB2*	4.85094
rs9940982	chr16	71415585	intronic	*CALB2*	4.9219
rs7200566	chr16	71426563	intergenic	*CALB2,ZNF23*	4.9219
rs35326792	chr16	71427061	intergenic	*CALB2,ZNF23*	5.01915
rs12445377	chr16	71428601	intergenic	*CALB2,ZNF23*	5.01915
rs891131	chr16	71432417	intergenic	*CALB2,ZNF23*	5.01915
rs2839619	chr21	44436177	intronic	*PKNOX1*	4.50955
rs3737434	chr21	44437142	intronic	*PKNOX1*	4.64333
rs2839624	chr21	44446030	intronic	*PKNOX1*	4.75923
rs2839625	chr21	44447347	intronic	*PKNOX1*	4.82339
rs2839626	chr21	44448384	intronic	*PKNOX1*	4.80037
rs2839627	chr21	44448718	intronic	*PKNOX1*	4.77703
rs234729	chr21	44449095	intronic	*PKNOX1*	4.77703
